# Receptor-Defined Subtypes of Breast Cancer in Indigenous Populations in Africa: A Systematic Review and Meta-Analysis

**DOI:** 10.1371/journal.pmed.1001720

**Published:** 2014-09-09

**Authors:** Amanda Eng, Valerie McCormack, Isabel dos-Santos-Silva

**Affiliations:** 1 Department of Non-Communicable Disease Epidemiology, London School of Hygiene & Tropical Medicine, London, United Kingdom; 2 Centre for Public Health Research, Massey University, Wellington, New Zealand; 3 Section of Environment and Radiation, International Agency for Research on Cancer, Lyon, France; Harvard School of Public Health, United States of America

## Abstract

**Background:**

Breast cancer is the most common female cancer in Africa. Receptor-defined subtypes are a major determinant of treatment options and disease outcomes but there is considerable uncertainty regarding the frequency of poor prognosis estrogen receptor (ER) negative subtypes in Africa. We systematically reviewed publications reporting on the frequency of breast cancer receptor-defined subtypes in indigenous populations in Africa.

**Methods and Findings:**

Medline, Embase, and Global Health were searched for studies published between 1st January 1980 and 15th April 2014. Reported proportions of ER positive (ER+), progesterone receptor positive (PR+), and human epidermal growth factor receptor-2 positive (HER2+) disease were extracted and 95% CI calculated. Random effects meta-analyses were used to pool estimates. Fifty-four studies from North Africa (*n* = 12,284 women with breast cancer) and 26 from sub-Saharan Africa (*n* = 4,737) were eligible. There was marked between-study heterogeneity in the ER+ estimates in both regions (I^2^>90%), with the majority reporting proportions between 0.40 and 0.80 in North Africa and between 0.20 and 0.70 in sub-Saharan Africa. Similarly, large between-study heterogeneity was observed for PR+ and HER2+ estimates (I^2^>80%, in all instances). Meta-regression analyses showed that the proportion of ER+ disease was 10% (4%–17%) lower for studies based on archived tumor blocks rather than prospectively collected specimens, and 9% (2%–17%) lower for those with ≥40% versus those with <40% grade 3 tumors. For prospectively collected samples, the pooled proportions for ER+ and triple negative tumors were 0.59 (0.56–0.62) and 0.21 (0.17–0.25), respectively, regardless of region. Limitations of the study include the lack of standardized procedures across the various studies; the low methodological quality of many studies in terms of the representativeness of their case series and the quality of the procedures for collection, fixation, and receptor testing; and the possibility that women with breast cancer may have contributed to more than one study.

**Conclusions:**

The published data from the more appropriate prospectively measured specimens are consistent with the majority of breast cancers in Africa being ER+. As no single subtype dominates in the continent availability of receptor testing should be a priority, especially for young women with early stage disease where appropriate receptor-specific treatment modalities offer the greatest potential for reducing years of life lost.

*Please see later in the article for the Editors' Summary*

## Abstract 2

In a systematic review and meta-analysis, Isabel dos Santos Silva and colleagues estimate the prevalence of receptor-defined subtypes of breast cancer in North Africa and sub-Saharan Africa.

*Please see later in the article for the Editors' Summary*

## Editors' Summary

**Background:**

Breast cancer is the commonest female tumor in Africa and death rates from the disease in some African countries are among the highest in the world. Breast cancer begins when cells in the breast acquire genetic changes that allow them to grow uncontrollably and to move around the body. When a breast lump is found (by mammography or manual examination), a few cells are collected from the lump (a biopsy) to look for abnormal cells and to test for the presence of estrogen receptor (ER), progesterone receptor (PR), and human epidermal growth factor receptor-2 (HER2) on the cells. The hormones estrogen and progesterone promote the growth of normal breast cells and of ER+ and PR+ breast cancer cells. HER2 also controls the growth of breast cells. The receptor status of breast cancer is a major determinant of treatment options and prognosis (likely outcome). ER+ tumors, for example, are more receptive to hormonal therapy and have a better prognosis than ER− tumors, whereas HER2+ tumors, which make large amounts of HER2, are more aggressive than HER2− tumors. Breast cancer is treated by surgically removing the lump or the whole breast (mastectomy) if the tumor has already spread, before killing any remaining cancer cells with chemotherapy or radiotherapy. In addition, ER+, PR+, and HER2+ tumors are treated with drugs that block these receptors (including tamoxifen and trastuzumab), thereby slowing breast cancer growth.

**Why Was This Study Done?:**

ER+ tumors predominate in white women but the proportion of ER+ tumors among US-born black women is slightly lower. The frequency of different receptor-defined subtypes of breast cancer in indigenous populations in Africa is currently unclear but policy makers need this information to help them decide whether routine receptor status testing should be introduced across Africa. Because receptor status is a major determination of treatment options and outcomes, it would be more important to introduce receptor testing if all subtypes are present in breast cancers in indigenous African women and if no one subtype dominates than if most breast cancers in these women are ER+. In this systematic review (a study that uses pre-defined criteria to identify all the research on a given topic) and meta-analysis (a statistical approach that combines the results of several studies), the researchers examine the distribution of receptor-defined breast cancer subtypes in indigenous populations in Africa.

**What Did the Researchers Do and Find?:**

The researchers identified 54 relevant studies from North Africa involving 12,284 women with breast cancer (mainly living in Egypt or Tunisia) and 26 studies from sub-Saharan Africa involving 4,737 women with breast cancer (mainly living in Nigeria or South Africa) and used the data from these studies to calculate the proportions of ER+, PR+, and HER2+ tumors (the number of receptor-positive tumors divided by the number of tumors with known receptor status) across Africa. The proportion of ER+ tumors varied markedly between studies, ranging between 0.40 and 0.80 in North Africa and between 0.20 and 0.70 in sub-Saharan Africa. Among prospectively collected samples (samples collected specifically for receptor-status testing; studies that determined the receptor status of breast cancers using stored samples reported a lower proportion of ER+ disease than studies that used prospectively collected samples), the overall pooled proportions of ER+ and triple negative tumors were 0.59 and 0.21, respectively.

**What Do These Findings Mean?:**

Although these findings highlight the scarcity of data on hormone receptor and HER2 status in breast cancers in indigenous African populations, they provide new information about the distribution of breast cancer subtypes in Africa. Specifically, these findings suggest that although slightly more than half of breast cancers in Africa are ER+, no single subtype dominates. They also suggest that the distribution of receptor-defined breast cancer subtypes in Africa is similar to that found in Western populations. The accuracy of these findings is likely to be affected by the low methodological quality of many of the studies and the lack of standardized procedures. Thus, large well-designed studies are still needed to accurately quantify the distribution of various breast cancer subtypes across Africa. In the meantime, the current findings support the introduction of routine receptor testing across Africa, especially for young women with early stage breast cancer in whom the potential to improve survival and reduce the years of life lost by knowing the receptor status of an individual's tumor is greatest.

**Additional Information:**

Please access these websites via the online version of this summary at http://dx.doi.org/10.1371/journal.pmed.1001720.

- This study is further discussed in a *PLOS Medicine* Perspective by Sulma i Mohammed
- The US National Cancer Institute (NCI) provides comprehensive information about cancer (in English and Spanish), including detailed information for patients and professionals about breast cancer including an online booklet for patients
- Cancer Research UK, a not-for profit organization, provides information about cancer; its detailed information about breast cancer includes sections on tests for hormone receptors and HER2 and on treatments that target hormone receptors and treatments that target HER2
- Breastcancer.org is a not-for-profit organization that provides up-to-date information about breast cancer (in English and Spanish), including information on hormone receptor status and HER2 status
- The UK National Health Service Choices website has information and personal stories about breast cancer; the not-for profit organization Healthtalkonline also provides personal stories about dealing with breast cancer

## Introduction

**Figure 1:**
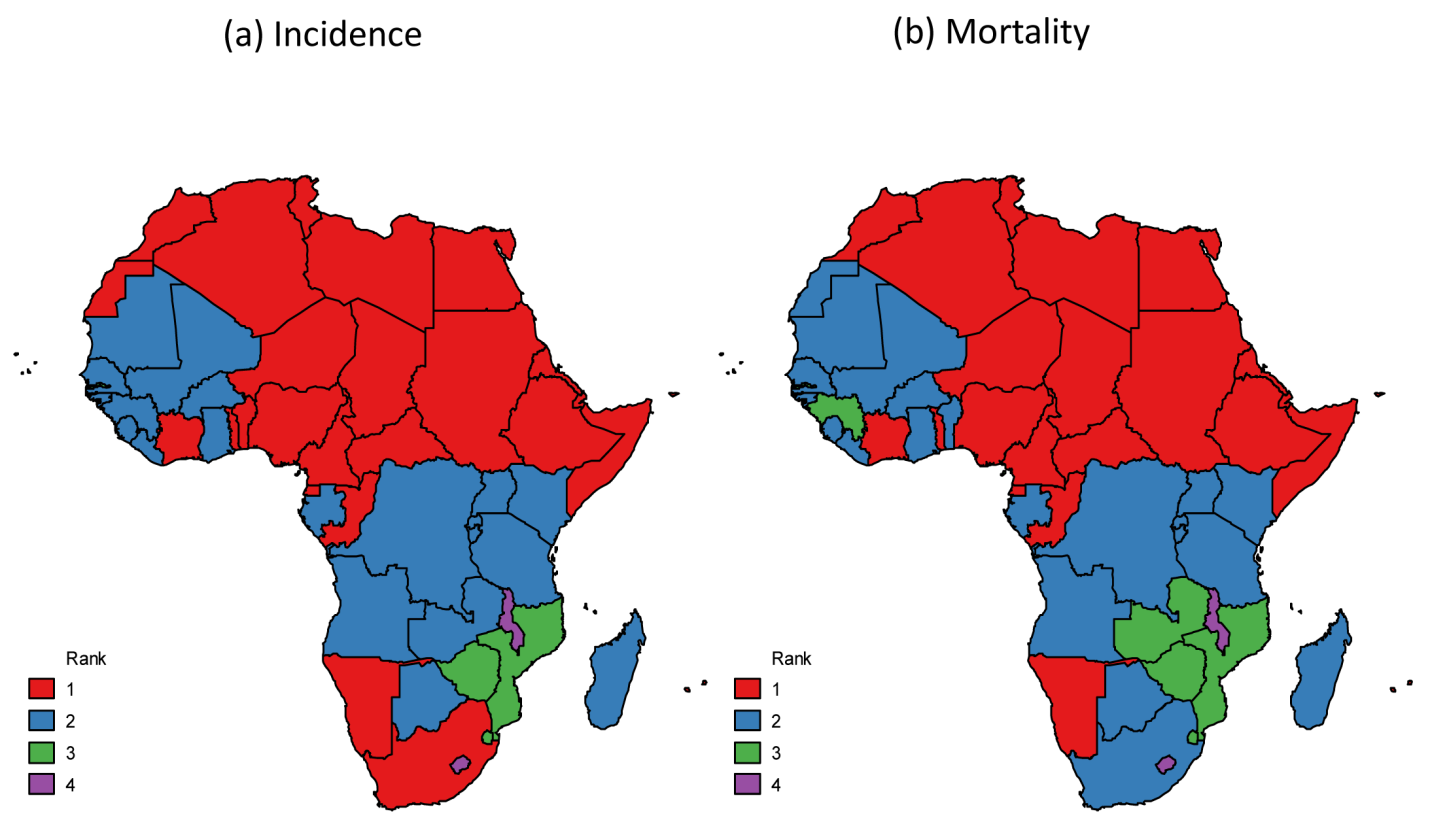
Breast cancer ranking among women for (a) incidence and (b) mortality, Africa, 2012 [1].

Breast cancer is the most common female malignancy in Africa, being the cancer with the first or second highest incidence and/or mortality in most African countries (Figure 1). Although breast cancer incidence rates are lower in Africa than in the rest of the world, mortality rates in certain African countries (e.g., Nigeria, Egypt, Ethiopia) are among the highest worldwide [1], reflecting the relatively poor survival from the disease in the continent. Different breast cancer subtypes are classified in the clinical setting by estrogen (ER), progesterone (PR), and human epidermal growth factor-2 (HER2) receptor status. These receptors are a fundamental characteristic of the epidemiology of this malignancy [2], as its aetiology and incidence trends are receptor-status specific, and they are also a major determinant of treatment options, disease outcomes, and survival [3].

ER-positive (ER+) tumors typically have a better prognosis and are more receptive to hormonal treatment [4]. In white (i.e., European ancestry) women, ER+ tumors predominate, with 79% of breast tumors in US-born white women being ER+ (calculated amongst women with known ER-status) [5]. The proportion of ER+ tumors is lower among US-born black (i.e., of African ancestry) women (61% are ER+, all ages combined) [5],[6], but the extent to which this is also reflected in Africa is not well-established. Some studies [7],[8] have reported a markedly higher proportion of ER-negative (ER−) or basal-like breast cancers in indigenous populations in Africa, which may contribute to the poor survival from this malignancy, but others suggest that the relative frequency of the different subtypes in the continent may not differ substantially to that seen elsewhere [9],[10].

Knowledge of the relative frequency of breast cancer subtypes in Africa would be of relevance for several reasons. Firstly, if the distribution of receptor status is greatly different in Africa than elsewhere, the differing contribution of genetic and environmental risk factors to such a difference would need to be investigated, as is debated for ethnic differences in the US [11]. Secondly, where tumor receptor status is not routinely ascertained, the need for introducing it would be more urgent if one subtype does not greatly dominate and all subtypes are present. The latter scenario would call for the introduction of receptor testing to be prioritised, especially for patients who would have the prospect of good survival if given the appropriate treatment. Knowledge of the distribution of tumor receptor subtypes in Africa would also be of relevance globally as the continent would provide a better setting to study any subtypes that are rare elsewhere, but may be common there.

In the absence of large standardized multi-country studies of breast cancer subtypes in Africa, a rigorous systematic review of previously published studies will provide the timeliest answer to the debate on the receptor status distribution in Africa. Herein, we systematically review all studies that have reported receptor status of breast cancer in indigenous African populations and assess sources of between-study heterogeneity in prevalence estimates based on more than 17,000 women with breast cancer.

## Methods

### Search Methodology

The PRISMA guidelines (Text S1) were used to develop the study protocol (Text S2). We conducted a search of Medline, Embase, and Global Health [12] of studies published between 1st January 1980 and 15th April 2014. After an initial search using specific keywords, the search was broadened to “breast cancer” in “Africa” (with each country individually named; Text S3) in order to capture the studies where receptor status was not the focus of the paper but likely to be reported under patients' characteristics. No language restrictions were imposed. In addition, we searched African Journals Online (AJO) and the Breast Health Global Initiative – INCTR Breast Cancer Control Library [13].

The titles and abstracts were reviewed by one author (AE) twice independently. Abstracts were excluded if the studies did not focus on breast cancer (e.g., studies of “all cancers”) or did not include women with breast cancer (e.g., surveys of attitudes towards breast screening); if they exclusively focused on: males, African-American women, metastatic breast cancer, pregnant women, or specific treatment groups; or if the total number of women with breast cancer included was <50. The latter were predominantly clinical reports or unrepresentative small case series of women with breast cancer who had been selected because of their unusual clinical or pathological characteristics (e.g., high-risk familial cases, BRCA1/2 carriers, bilateral cases, gestational breast cancers), and were also more likely to have arisen from settings where there was less quality control in laboratory procedures for fixation and immunohistochemistry (IHC). Studies were also excluded if they focused exclusively on non-black populations (e.g., white or coloured women in South Africa). Reviews and conference proceedings were not included, but their references were cross-checked. A random sample of 80 titles/abstracts was also reviewed independently by another author (IdSS); this review revealed high between-reviewer reproducibility with no disagreements on which papers to select for full text review. The full text was retrieved for all potentially relevant papers and reviewed by the same author (AE) for reporting of receptor status. If there were multiple papers from the same study the paper with the most information on receptor status was selected for inclusion.

### Data Extraction

The data extraction from each eligible paper was carried out independently by two reviewers (AE and IdSS or VM and IdSS) using a specifically developed and pre-tested computerised data extraction form (Text S2). Data were extracted on the number of women with breast cancer with available receptor status information, and the number of those with positive and negative tumors, as classified in the original article regardless of the criteria used to define positivity (Tables 1 and 2), for ER (ER+/ER−), PR (PR+/PR−), and HER2 (HER2+/HER2−) and, where available, for combined subtypes: luminal A (ER+ and/or PR+; HER2−), luminal B (ER+ and/or PR+; HER2+), HER2+-enriched (ER−; PR−; HER2+), and triple negative (ER−; PR−; HER2−). Information was also extracted on type of study, including study design (e.g., population-based, case series based on consecutive women diagnosed with breast cancer over a defined time period, or collection based on convenience [opportunistic] samples), source of the breast cancer patients (e.g., hospital/clinic or cancer registry), sample size and study period; tumor characteristics (e.g., histological type; tumor size, stage, and grade); collection and storage conditions of the tumor specimens (e.g., fresh-frozen, formalin-fixed paraffin-embedded [FFPE] blocks); receptor testing (e.g., timing, type of assay, positivity criteria); and on demographic and reproductive-related variables (e.g., ethnicity, age, and menopausal status at diagnosis) where available. Many studies had limited information on how women with breast cancer were selected, or on the time period from tumor specimen collection to receptor testing, and the details provided in their methods section were used to obtain as informed a description as was possible. We did not attempt to contact the authors because most of the missing information was from studies published in the early years, making it difficult to establish contact and unlikely that the missing information would still be available. A few studies included a small number of men with breast cancer; these were included in the review as the papers did not provide enough information to allow their exclusion. Disagreements were discussed by both reviewers and a consensus reached.

**Table 1:** Characteristics of the participating studies: North Africa (54 studies).

First Author, Year [Ref]	Country	Study Design	Sample Size	Method For ER And/Or PR	Criteria For ER/PR Positivity	HER2Testing: Y/N	Method(S) For HER2	Criteria For HER2+Positivity	ER Quality Score	PR Quality Score	HER2Quality Score
Ismaili 2014-IBC [68]	Morocco	Consecutive case series	64	NK	NK	Y	NK	NK	15	15	13
Tazzite 2013 [44]	Morocco	Convenient case series	570	NK (ER and PR extracted from MR)	NK	N	n/a	n/a	12	12	
Bennis 2012 [34]	Morocco	Convenient case series	366	IHC (ER: Immunotech, clone 1D5; PR: Immunotech, clone 10A9)	≥10% nuclei staining	Y	IHC (Dako, clone A0485)	Score 3+; score 2+ and FISH+	17	17	17
Boufettal et al, 2010 [26]	Morocco	Convenient case series	451	NK (ER and PR extracted from MR)	NK	N^a^	n/a	n/a	13	13	
Bouzid 2013 [64]	Tunisia	Consecutive case series	99	NK; HR^b^ reported	NK	N	n/a	n/a	15	15	
Ben Gacem 2012 [21]	Tunisia	Convenient case series	94	IHC (ER and PR, NOS)	≥10% nuclei staining	Y	IHC (NOS)	Score ≥2+	18	18	17
Karray-Chouayekh 2011 [32]	Tunisia	Convenient case series	80	IHC: ER (Dako, clone 1D5, 1∶25), PR (Dako, clone PgR636, 1∶50)	>5% nuclei staining	Y	IHC (Dako, clone 124, 1∶100)	Score 3+ (intense and complete membrane staining in >30% cells)	13	13	12
Hamrita 2011 [23]	Tunisia	Convenient case series	287	NK (ER and PR extracted from MR)	NK	N	n/a	n/a	12	12	
Karray-Chouayekh 2010 [41]	Tunisia	Convenient case series	78	IHC (ER: Dako, clone 1D5, 1∶25; PR: Dako, clone PgR636, 1∶50)	>5% nuclei staining	Y	IHC (Dako, clone 124, 1∶100)	Score 3+ (intense and complete membrane staining in >30% cells)	17	17	16
Loueslati 2010 [46]	Tunisia	Convenient case series	70	IHC (ER and PR, NOS)	NK	N	n/a	n/a	11	11	
Marrakchi 2010 [27]	Tunisia	Convenient case series	127	NK (ER and PR)	NK	N	NK	NK	15	15	
Abdelkrim 2010 [47]	Tunisia	Convenient case series	194	IHC (ER and PR, NOS)	≥10% nuclei staining	Y	IHC (NOS)	Score 3+ (intense and complete membrane staining in >10% cells)	19	19	18
Snoussi 2010 [22]	Tunisia	Consecutive case series	297	NK (ER extracted from MR)	NK	N	n/a	n/a	14		
Kallel 2010 [53]	Tunisia	Convenient case series	133	IHC (ER: Dako, clone 1D5; PR: Dako, clone PgR636)	>5% nuclei staining	Y	IHC (ACRIS, clone BM5084)	>5% cell staining	18	18	15
Hachana 2008 [24]	Tunisia	Convenient case series	122	IHC (ER: Dako, clone 1D5, 1∶40; PR: Dako, clone PgR636. 1∶40)	≥10% nuclei staining	Y	IHC (Dako, polyclonal, 1∶1000)	Score ≥2+	16	16	15
Ben Hamida 2008 [28]	Tunisia/France (methods reported for Tunisia only)	Convenient case series	78	IHC (ER: Novocastra, clone 6F11.2, 1∶60; PR: Dako, clone PFR636, 1∶80)	≥1% nuclei staining	Y	IHC (Dako, clone AO485, 1∶500)	Score ≥2+	19	19	18
Ayadi 2008 [39]	Tunisia	Convenient case series	155	IHC (ER: Dako, clone 1D5, 1∶25; PR: Dako, clone PgR636, 1∶50)	Allred scoring method, NOS	Y	IHC (Dako, clone 124, 1∶100)	Score 3+ (intense and complete membrane staining in >30% cells)	16	16	15
Marrakchi 2008 [35]	Tunisia	Convenient case series	80	NK (ER and PR)	NK	N	n/a	n/a	15	15	
Maleej 2008 [37]	Tunisia	Population-based	938	NK (ER and PR extracted from MR)	NK	N	n/a	n/a	13	13	
Le 2005 [30]	Tunisia/France. (methods reported for Tunisia only)	Consecutive case series	172	NK (ER and PR)	NK	N	n/a	n/a	16	16	
Baccouche 2003 [36]	Tunisia	Convenient case series	50	IHC (ER: Dako, clone 1D5)	>20% nuclei staining	N	n/a	n/a	15		
McCarthy 2002 [19]	Tunisia	Convenient case series	66	IHC (ER: BioGenex, clone 6F11)	>10% nuclei staining	Y	IHC (Zymed, a mix of mouse TAB250 and PAD24881 rabbit serum	Score 3+ (intense staining in >10% cells)	16		15
Boder 2013 [63]	Libya	Convenient case series	130	NK; HR^b^ reported	NK	N	n/a	n/a	15	15	
Ermiah 2013 [57]	Libya	Convenient case series	170	IHC (ER and PR extracted from MR)	Allred scoring method, NOS	N	n/a	n/a	19	19	
Moona 2010 [42]	Libya	Convenient case series	78	ICH (ER and PR extracted from MR)	NK	Y	ICH and FISH	NK	11	11	11
Alieldin 2014 [62]	Egypt	Consecutive case series	617	IHC or enzyme immunoassay (ER and PR extracted from MR)	NK	N	n/a	n/a	15	15	
Hirko 2013 [72],[73]	Egypt	Population-based	3,060	IHC (monoclonal antibodes for ER and PR)	>1% nuclei staining	N	n/a	n/a	18	18	
Elesawy 2014 [65]	Egypt	Convenient case series	125	IHC (ER: Dako, clone 1D5, 1∶50; PR: Dako, clone PgR636, 1∶50)	≥1% nuclei staining	Y	IHC (CB11, Novocastra, 1∶50)	Score 3+; score 2+ and FISH+	16	16	16
Hagrass 2014 [67]	Egypt	Consecutive case series	120	IHC (ER: mouse monoclonal IgG, PR: rabbit polyclonal IgG, Santa Cruz)	NK	Y	IHC (Mousemonoclonal IgG,Santa Cruz	NK	20	20	20
Rashad 2014 [69]	Egypt	Convenient case series	80	IHC (ER and PR: monoclonal)	>10% nuclei staining	Y	IHC (mAb CB11)	Score 3+; score 2+ and FISH+	19	19	19
El-Shinawi 2013 [66]	Egypt	Convenient case series	77	IHC (ER and PR, NOS)	>10% nuclei staining	Y	IHC (NOS)	>10% membrane staining of tumor cells	16	16	15
Hussein 2013 [52]	Egypt	Convenient case series	263	IHC (ER: Lab Vision, clone SP1; PR: Lab Vision, clone Ab-2)^b^	>1% nuclei staining	Y	IHC (Lab Vision Ab-17, clone e2-4001+3B5)	>30% cells staining	17	17	16
Salama 2013 [71]	Egypt	Consecutive case series	99	IHC (primary ER and PR antibodies, Labvision, thermoscientific); HR^b^ reported	>1% nuclei staining	Y	IHC (Dako)	Score 3+	18	18	17
El-Hawary 2012 [33]	Egypt	Convenient case series	274	IHC (ER: Cell Marque, clone SP1; PR: Dako, clone PgR636)^c^	Allred scoring method, NOS	Y	IHC (Cell Marque, clone CB-11)	Guidelines of the American Society of Clinical Oncology, NOS	14	14	13
Salhia 2011 [49]	Egypt	Convenient case series	203	IHC (ER: Dako K1904; PR: Dako K1904)	≥1% nuclei staining	Y	IHC (Dako, clone A0485, 1∶100)	Scores ≥2+ (weak or intensecomplete staining of the membrane in >10% of cells	16	16	15
Abbas 2011 [29]	Egypt	Convenient case series	129	NK (HR^b^ extracted from MR)	NK	N	n/a	n/a	12	12	
Hussein 2011 [31]	Egypt	Convenient case series	96	NK (ER extracted from MR)	NK	N	n/a	n/a	13		
El Mongy 2010 [50]	Egypt	Consecutive case series	934	NK (ER and PR extracted from MR)	NK	N	n/a	n/a	16	16	
Hafez 2010 [59]	Egypt	Convenient case series	90	IHC^d^ (ER and PR, NOS)	NK	N	n/a	n/a	12	12	
El-Rehim 2009 [38]	Egypt	Convenient case series	65	IHC (ER: Dako, clone 1D5, 1∶80; PR: Dako, clone 636, 1∶100)	Histoscore based on intensity (1+ to 3+) and % of cells stained positive (0%–100%).Positive if expression rates >10%	N	n/a	n/a	17	17	
Zeeneldin 2009 [43]	Egypt	Consecutive case series	57	IHC (HR^b^, NOS)	>5% nuclei staining	Y	IHC (HercepTest)	Unclear if positive for scores ≥2+,or only for scores 3+	23	23	21
Ali-Labib 2009 [25]	Egypt	Convenient case series	50	NK (ER extracted from MR)	NK	N	n/a	n/a	16		
Marzouk 2009 [54]	Egypt	Consecutive case series	174	IHC (ER and PR, NOS)	NK	N	n/a	n/a	16	16	
Youssef 2008 [56]	Egypt	Convenient case series	65	NK (ER and PR extracted from MR)	NK	Y	NK (extracted from MR	NK	17	17	15
Rashed 2007 [45]	Egypt	Convenient case series	50	IHC (ER: Ventana, clone 6F11, 1∶40; PR:Ventana, 1α6, 1∶30)	>10% nuclei staining	Y	IHC (Ventana, clone DA485, 1∶1500)	Score 3+ (intense and complete membrane staining in >10% cells)	16	16	15
Mohammad 2006 [40]	Egypt	Convenient case series	64	IHC (ER: Dako, clone 1D5; PR: Dako, clone 1A6)	>5% nuclei staining	Y	IHC (Dako, HercepTest)	Score ≥2+	15	15	14
Swellam 2004 [60]	Egypt	Convenient case series	51	Abbott enzyme immunoassay (ER and PR)	>15 fmol/mg protein	N	n/a	n/a	19	19	
Asaad 2003 [58]	Egypt	Convenient case series	44	NK (ER and PR extracted from MR)	NK	N	n/a	n/a	13	13	
Abdel-Fattah 2001 [61]	Egypt	Consecutive case series	19	NK (ER and PR extracted from MR)	NK	N	n/a	n/a	11	11	
Abu-Bedair 2000 [51]	Egypt	Convenient case series	71	Radiorceptor assay (ER: ^125^I-radioreceptor assay kit, DSL)	Cytosols with saturable binding ≥10 fmol^125^I-17beta-estradiol per mg protein	N	n/a	n/a	16		
Bekkouche 2013 [70]	Algeria	Consecutive case series	120	IHC (ER: 1D5, Dako code 1575; PR: PgR636,Dako code 1630)	NK	Y	IHC (Polyclonal anti-human C-erbB2 Dako A0485	Score 3+	17	17	17
Chaher 2012 [55]	Algeria	Convenient case series	176	IHC (ER: Thermo Scientific, clone RB-9016, 1∶100; PR: Dako, clone PgR 636, 1∶100)	>10% nuclear staining	Y	IHC (Ventana, clone 4B5)	Score 3+; scores 1+ or 2+, and CISH+	19	19	19
Elgaili 2010 [20]	Sudan	Population-based	48	IHC (ER: Novocostra, clone ID5, 1∶25; PR: Novocastra, clone IA6, 1∶40)	% of epithelial cells with positive staining scored as 0 = no, 1 = weak, 2 = moderate, and 3 = strong staining. Unclear if scores>0 were taken as positive.	N	n/a	n/a	16	16	
Awadelkarim 2008 [48]	Sudan/Italy. (Methods presented for Sudan only)	Consecutive case series	114	IHC (ER: Dako, clone 1D5, 1∶100; PR: clone PgR 636, 1∶100)	>5% nuclei staining	Y	IHC (Dako, polyclonal antiserum, 1∶350)	Score 3+; score 2+ and FISH+	19	19	19

a HER2 testing was performed but not included in the review because it was not possible to estimate the standard error as the sample size was NK.

b Only HR+ (ER+ and/or PR+) estimates are provided.

c Only HR+ (defined as subtypes Luminal A + Luminal B) estimates are provided.

d Only the results from IHC (versus ICH) were included in the review.

AJCC, American Joint Committee on Cancer; BC, breast cancer; IBC, inflammatory breast cancer; IDC, invasive ductal carcinoma; ICH, immunocytochemistry; IgG, Immunoglobulin G; MR, medical records; n/a, not applicable; NK, not given in the paper; NOS, not otherwise specified.

**Table 2:** Characteristics of the participating studies: Sub-Saharan Africa (26 studies).

First Author, Year [Ref]	Country	Study Design	Sample Size	Method For ER And/Or PR	Criteria For ER/PR Positivity	HER2Testing: Y/N	Method(S) For HER2	Criteria For HER2+ Positivity	ER Quality Score	PR Quality Score	HER2Quality Score
Ly 2012 [84]	Mali	Consecutive case series	113	IHC (ER: Novocastra, clone 6F11 (NCL-ER 6F11), 1∶50; PR: Novocastra, clone 16 (NCL- PRG312), 1∶50)	>10% nuclei staining	Y	IHC (Dako A0485,polyclonal, 1∶1000)	Score 3+ (>30% cells staining); score 2+ and FISH+	19	19	19
Togo 2010 [94]	Mali	Consecutive case series	160	NK (ER and PR extracted from MR)	NK	N	n/a	n/a	14	14	
Ugiagbe 2012 [74]	Nigeria	Convenient case series	135	IHC (ER and PR: Dako, NOS)	>10% nuclei staining	N	n/a	n/a	16	16	
Agboola 2012 [76]	Nigeria/UK (Methods presented for Nigeria only)	Convenient case series	274	IHC (ER: Dako, clone 1D5, 1∶200; PR: Dako, clone PgR, 1∶150)	≥1% cells stained	Y	IHC (Dako, polyclonal, 1∶100)	Score 3+; score 2+ and CISH+	15	15	15
Huo 2009 [8]	Nigeria and Senegal (series 1 only)^a^	Consecutive case series	378	IHC (ER: NeoMarkers, SP1 clone, 1∶50; PR: Neomarkers, SP2 clone, 1∶50)	Unclear (semi-quantitative score using Reiner's 4-point scale based on intensity and % of IHC reaction)	Y	IHC (Dako, HercepTest)	“According to manufacturer's instructions (DAKO)” (sic)	18	18	16
Adebamowo 2008 [10]	Nigeria	Consecutive case series	177	IHC (ER: Zymed, clone 1D5; PR: Zymed, clone 2C5)	>10% nuclei staining	Y	IHC (Zymed, clone Z4881)	Score 3+ (intense and complete membrane staining in >10% of cells)	19	19	18
Iyare 2007 [75]	Nigeria	Convenient case series	102	IHC (ER and PR, NOS)	NK	Y	IHC (NOS)	NK	13	13	13
Gukas 2005 [79]	Nigeria	Consecutive case series	36	IHC (ER: Novocastra, clone ER6F11, 1∶15; PR: Dako, PGR636, 1∶50)	≥10% nuclei staining	Y	IHC (Dako, clone Polyclonal, 1∶1000)	≥5% cells staining	19	19	18
Ikpatt 2003 [77]	Nigeria	Convenient case series	129	IHC (ER: Novocastra, clone CC4-5 (NCL-ER− LH2), 1∶80; PR: Novocastra, clone 1A6 (NCL- PGR), 1∶20)	Total score: staining intensity score (0–3)+percentage of positive cell score (0–4).Positive: total score ≥2	N	n/a	n/a	19	19	
Ohene-Yeboah 2012 [87]	Ghana	Consecutive case series	68	IHC (ER and PR: commercially available kits, NOS)	≥10% nuclei staining	Y	IHC (commercially available kits, NOS)	Score 3+; Score 2+ and SISH+	17	17	17
Schwartz 2013 [80]	Ghana	Convenient case series	103	IHC (ER: Dako, clone ID5, 1∶50; PR: Dako, clone PgR636, 1∶50)	≥2% nuclei staining	Y	IHC (Dako, 1∶100)	Score 3+ (no samples scored 2+)	16	16	15
Stark 2010 [78]	Ghana/US (Methods reported for Ghana only)	Convenient case series	75	IHC (ER: Dako, clone ID5; PR: Dako, clone PgR636)	% of nuclei staining assessed semi-quantitatively as positive (including focal positive) or negative.	Y	IHC (Dako, HercepTest)	Score 3+ (strong complete membrane staining in >10% cells); score 1+ or 2+ and FISH+	14	12	14
Yarney 2008 [85]	Ghana	Convenient case series	74	IHC (ER and PR, NOS)	Quick score ≥3	Y	IHC (NOS)	Score 3+	13	13	12
Galukande 2013 [86]	Uganda	Consecutive case series	113	IHC (ER^b^: Cell Marquee, clone SP-I)	≥5% nuclei staining	N^b^	n/a	n/a	21		
Nalwoga 2010 [83]	Uganda	Population-based	183	IHC (ER: Dako, clone ID5, dilution 1∶50; PR: Dako, clone PgR 636, dilution 1∶150)	≥10% nuclei staining	Y	IHC (Dako, clone Polyclonal, 1∶500)	Scores 2+ and 3+ (>10% cells stained)	18	18	17
Bird 2008 [7]	Kenya	Consecutive case series	120	IHC (ER: Dako, clone 1D5, 1∶50; PR: Dako, clone MO A-HU, 1∶30)	IHC score ≥1	Y	IHC (Dako, clone AO48529, 1∶200)	Scores 2+ and 3+	19		16
Nyagol 2006 [82]	Kenya	Consecutive case series	158	IHC (ER: Dako, clone 1D5, 1∶50; PR: Dako, clone MO A-HU, 1∶30)	>10% nuclei staining	Y	IHC (Dako, clone AO48529, 1∶200)	Score 3+: (complete membrane staining in>10% cells); score 2+ and FISH+	18	18	18
Burson 2010 [88]	Tanzania	Convenient case series	65	IHC (ER: Dako, clone 1D5, 1∶100; PR: Dako, clone 636, 1∶200)	Total Allred score: staining intensity score (0–3)+percentage of positive cell score(0–5). Positive: total score >2	N	n/a	n/a	14	14	
Mbonde 2001 [81]	Tanzania	Convenient case series	60	IHC (ER and PR: Dako, NOS)	Total score: staining intensity score (0–3)+percentage of positive cell score (0–3). Positive: total score ≥3	N	n/a	n/a	15	15	
van Bogaert 2013 [92]	South Africa	Population-based	769	IHC (ER and PR, NOS)	NK	Y	IHC (NOS)	NK	12		12
McCormack 2013 [9]	South Africa. (Methods reported for black women only)	Consecutive case series	957	IHC (ER and PR: Ventana, NOS)	>1% nuclei staining	Y	IHC (Ventana, NOS)	Score 3+	25	25	24
Basro 2010 [93]	South Africa	Consecutive case series	118	IHC (ER and PR, NOS)	NK	Y	IHC (NOS)	NK	17	15	13
Winters 1988 [90]	South Africa	Consecutive case series	65	Radioligand binding assay (ER)	Oestradiol binding value >3 fmol/mg of cytosol protein.	N	n/a	n/a	17		
Savage 1981 [89]	South Africa. (Methods reported for black women only)	Convenient case series	170	Radioligand binding assay (ER: DCC method)	Positive if results showed a Scatchard plot, a K_d_ <5×10^10^ M and an oestradiol-binding value >3 fmol/mg protein	N	n/a	n/a	15		
Collings 1980 [91]	South Africa. (Methods reported for black women only)	Convenient case series	60	ERc assay (ER: DCC method)	Positive if results showed a Scatchard plot, a K_d_<5×10^−10^, an oestradiol-binding value >3 fmol/mg protein and a binding index of >12%	N	n/a	n/a	19		
Emile Hasiniatsy 2014 [95]	Madagascar	Consecutive case series	75	IHC (DAB revelation with Automate Ventana <~!?show=[sr]?>Benchmark with ER or PR antibodies)	>1% nuclei staining	N	n/a	n/a	17	17	

a Only Series 1 (*n* = 378) was included in the review. Series 2 is a replicate sample (*n* = 129) which was excluded because of a) potential overlap with other studies included in the review; and b) the number of ER+, PR+ and HER2+ known was not reported.

b Methods for PR and HER2 testing are provided in the paper but no estimates for these two receptors are given.

AJCC, American Joint Committee on Cancer; BC, Breast cancer; IBC, inflammatory breast cancer; IDC, invasive ductal carcinoma; ICH, immunocytochemistry; MR, medical records; n/a, not applicable; NK, not given in the paper; NOS, not otherwise specified.

### Study Quality

We adopted an approach similar to that used by the Cochrane Collaboration to develop a standardised quality assessment form for assessing the risk of bias in randomised studies [14]. We identified items within three quality domains to reflect the potential for selection bias, misclassification of receptor status, and availability of data on key correlates of receptor status. A list of items for each one of the three domains was developed. For each item, papers were allocated a score ranging from 0 (if it did not meet the criteria or if the information provided was unclear) to a maximum of 2 or 4, depending on the item, with more weight given to items in the selection bias and misclassification domains. Items in the selection bias domain included study design/case selection (score 0 if unclear; 2, if opportunistic case series; 4, if consecutive or population-based case series) and percentage of patients with known receptor status (score 0, if unclear; 2, if <70%; 4, if ≥70%). Items in the misclassification domain comprised timing of tumor specimen collection (score 0, if inferred that tumor samples were collected prior to the start of treatment but this is not clearly stated—studies stating that collection was done after treatment were excluded from the review; 2, if specified that collection was done prior to treatment onset); tumor tissue storage conditions (score 0, if unclear; 2, if FFPE; 4, if frozen); timing of receptor status testing (score 2, if retrospective based on archival samples; 4, if conducted at the time of diagnosis); assay method (score 0, if not given; 2, if method described); criteria used to ascertain receptor positivity (for ER and PR: score 0, if not given; 2, if criteria described; for HER2: score 0, if not given; 1, if criteria described but fluorescent in situ hybridization [FISH] [chromogenic in situ hybridization (CISH) or silver in situ hybridization (SISH)] not used; 2, if FISH [CISH or SISH] used). The domain on correlates of receptor status comprised availability of information on age and/or menopausal status, tumor grade, and tumor stage (all scored as 0 if missing, 1 if available). The overall quality of the study was expressed as the sum of its item-specific scores. The range of possible scores was from 0 (lowest) to 25 (highest); the higher the score the higher the methodological quality of the study and, hence, the lower the risk that its findings might have been affected by bias.

Two authors (AE and IdSS) reviewed the quality of individual studies and inconsistencies discussed to reach consensus. In the analysis, we opted for simply describing the distribution of scores for studies reporting on each specific receptor, rather than using an arbitrary cut-off to define high versus low quality studies, and for examining both the contribution of the overall quality score and of specific quality criteria to between-study heterogeneity in estimates.

### Statistical Methods

As previous studies suggested differential ER+ proportions in women of African, rather than Arabic origin, results are presented separately for North Africa (i.e., Algeria, Egypt, Libya, Morocco, Sudan, Tunisia, and Western Sahara) and sub-Saharan Africa (i.e., all remaining African countries) according to their predominant population groups as defined by the United Nations [15]. For each receptor, the proportion of receptor-positive breast cancers (*prop*) was the statistic of interest, calculated as (number of receptor−positive tumors)/(*n* = number of tumors with known receptor status). Wilson score 95% CIs for this binomial *prop* were calculated and, on the basis of these, meta analyses were conducted in STATA version 12 (StataCorp), using the *metaprop* command to estimate pooled proportions using random effects models. Between-study heterogeneity was assessed using I^2^ (with its 95% CI estimated by the method of Higgins and Thomson [16]) and the *p*-value for heterogeneity (Cochrane's Q statistic). The I^2^ statistic represents the percentage of between-study variation due to heterogeneity rather than chance [17]. To examine potential sources of heterogeneity, study-specific estimates were stratified according to *a priori* defined geographical (i.e., two *ad hoc* sub-regions within North Africa—North-Eastern and North-Western—and three sub-regions in sub-Saharan Africa—Eastern, Southern, and Western—as defined by the United Nations [15]; see Results section), clinical factors (e.g., age, year, and menopausal status at diagnosis, tumor stage, and grade) and methodologically relevant variables (e.g., study design, timing of receptor testing, specimen storage conditions, study quality). Few studies provided information on reproductive-related variables except menopausal status; if data on the latter variable were not available, women aged >50 years were classified as post-menopausal. Meta-regression analyses were conducted to identify independent sources of between-study heterogeneity. These analyses necessitated an assumption of a single standard error that was estimated as √{*prop*(1−*prop*)/n}. Funnel plots and the Egger test [18] were performed to examine whether small study bias could have affected the results.

## Results

### Characteristics of Included Studies

**Figure 2:**
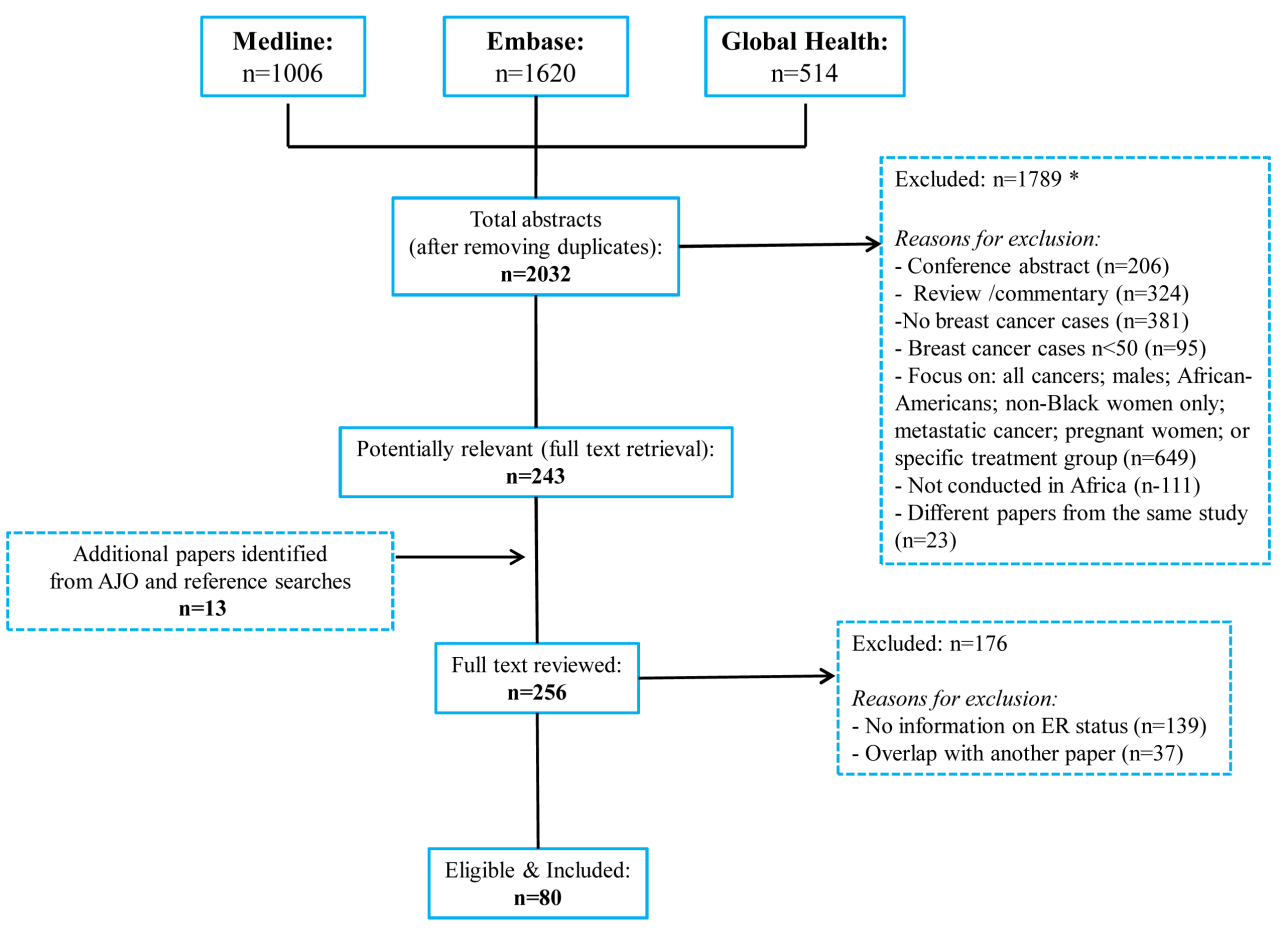
Flow diagram detailing study identification, screening, and eligibility. Many abstracts could fit into more than one exclusion category; these were allocated to the first eligible category in the order listed here.

**Figure 3:**
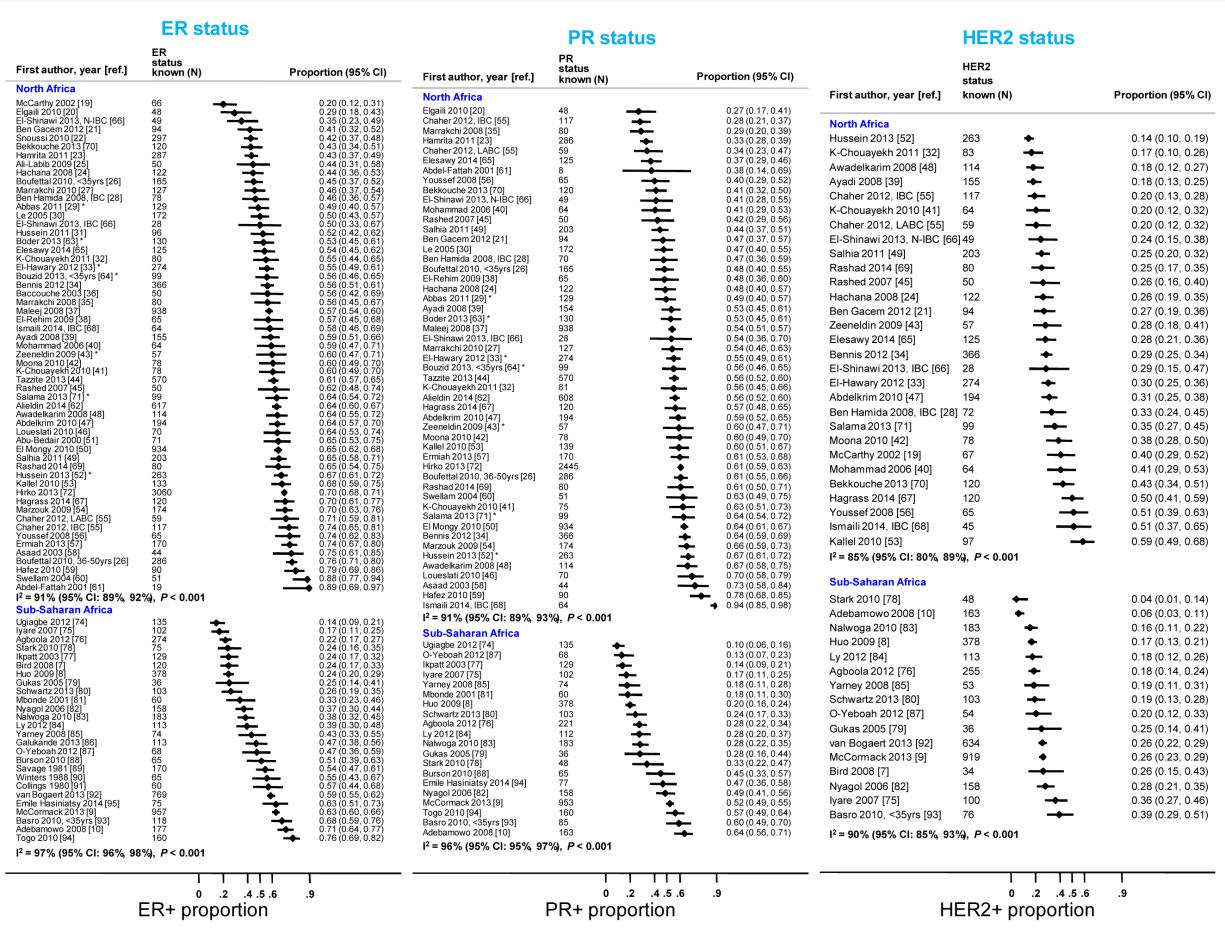
Proportion of ER+, PR+, and HER2+ disease (ranked by increasing magnitude), North and sub-Saharan Africa. IBC, inflammatory breast cancer; LABC, non-IBC locally advanced breast cancer; N-IBC, non-inflammatory breast cancer. *These studies provided only a combined HR estimate for tumors that were either ER+ or PR+ [33] or ER+ and/or PR+ ([29]; [43]; [52]; [63]; [64]; [71]). These HR estimates were included in both the ER+ and PR+ plots.

The systematic search in Medline, Embase, and Global Health produced 2,032 abstracts, of which 243 were identified as potentially relevant and the full text reviewed (Figure 2). A further 13 studies were identified from African Journals Online or hand-searches of bibliographic references. Eighty studies reported on ER status (no studies reported on PR or HER2 status without also reporting on ER status) and were therefore included in the review, involving a total of 17,021 women with breast cancer. Tables 1 and 2 present the characteristics of each one of the 80 participating studies. Fifty-four studies from North Africa [19]–[73] and 26 from sub-Saharan Africa [7]–[10],[74]–[95] reported on ER status, with fewer also reporting on PR or HER2 status (Figure 3; Tables 1, 2, and 3). Eighty percent of the North African studies, corresponding to 81% of all women with breast cancer from this region, were conducted in Egypt or Tunisia; 50% of the sub-Saharan African studies, corresponding to 71% of women with breast cancer from the region, were from South Africa or Nigeria (the distribution by country is given in Table 3). Most studies had sample sizes <300 patients with known receptor status. Only four studies [9],[37],[50],[72],[73] had >900 women with breast cancer, with the largest one (*n* = 3,060) also being one of the few to be based on a population-based cancer registry (an Egyptian study [72],[73]). The most common method for assessing receptor status was monoclonal assays (i.e., the quantitative enzyme immunoassay and, more often, the semi-quantitative IHC approach), but ER status was ascertained by ligand binding assays (e.g., dextran-coated charcoal [DCC] method) in some earlier studies (Tables 1 and 2) [51],[89]–[91]. FISH, CISH, or SISH to ascertain the HER2 status of specimens with an equivocal IHC score of 2+ was only performed in a few studies (Tables 1 and 2) [34],[42],[48],[55],[65],[69],[76],[78],[82],[84],[87].

**Table 3:** Summary of the characteristics of the 80 participating studies.

Variable	North Africa						Sub-Saharan Africa					
	ER Status		PR Status		HER2 Status		ER Status		PR Status		HER2 Status	
	Number of Studies	Number of *(%)* a Cases	Number of Studies	Number of *(%)* a Cases	Number of Studies	Number of *(%)* a Cases	Number of Studies	Number of *(%)* a Cases	Number of Studies	Number of *(%)* a Cases	Number of Studies	Number of *(%)* a Cases
**Total**	54	12,284 *(100)*	48	11,013 *(100)*	27	3,324 *(100)*	26	4,737 (100)	20	3,310 (100)	16	3,307 (100)
**Country**												
North Africa:												
Egypt	25	6,877 (56)	22	6,025 (55)	12	1,477 (44)						
Tunisia	18	3,120 (25)	15	2,701 (25)	9	948 (29)						
Others^b^	11	2,287 (19)	11	2,287 *(21)*	6	899 *(27)*						
Sub-Sahara Africa:												
South Africa							6	2,139 (45)	2	1,038 (31)	3	1,629 (49)
Nigeria^c^							7	1,231 (26)	7	1,164 (35)	5	932 (28)
Others^d^							13	1,367 (29)	11	1,108 *(33)*	8	746 *(23)*
**Study design**												
Population-based (e.g., cancer registry)	3	4,046 *(33)*	3	3,431 *(31)*	0	0 *(0)*	2	952 *(20)*	1	183 *(6)*	2	817 *(25)*
Consecutive case series	13	2,886 *(23)*	12	2,569 *(23)*	6	555 *(17)*	13	2,538 *(54)*	10	2,190 *(66)*	9	1,931 *(58)*
Convenience case series	38	5,352 *(44)*	33	5,013 *(46)*	21	2,769 (*83)*	11	1,247 *(26)*	9	937 *(28)*	5	559 *(17)*
**Year of diagnosis** e												
Before 2000	4	335 *(3)*	3	250 *(2)*	2	139 *(4)*	5	484 *(10)*	2	189 *(6)*	0	0 *(0)*
2001–2007	17	4,182 *(34)*	16	3,881 *(35)*	7	674 *(20)*	8	1,327 *(28)*	7	1,193 *(36)*	7	1,069 *(32)*
2008+	20	6,635 *(54)*	19	5,924 *(54)*	14	2,220 *(68)*	11	2,121 (*45)*	10	1,892 *(57)*	7	1,568 *(47)*
*Not known*	*13*	*1,132 (9)*	*10*	*958 (9)*	*4*	*291 (9)*	*2*	*805 (17)*	*1*	*36 (1)*	*2*	*670 (20)*
**Menopausal status at presentation** f												
<60% cases were postmenopausal	30	8,856 *(72)*	27	7,774 *(71)*	13	1,292 *(39)*	14	1,862 *(39)*	12	1,492 *(45)*	8	992 *(30)*
≥60% cases were postmenopausal	2	412 *(3)*	2	411 (4)	1	125 *(4)*	4	1,142 *(24)*	2	1,013 *(31)*	1	919 *(28)*
*Not known*	*22*	*3,016 (25)*	*19*	*2,828 (26)*	*13*	*1,907 (57)*	*8*	*1,733 (37)*	*6*	*805 (24)*	*7*	*1,396 (42)*
**Stage at presentation**												
<60% cases with stage 3 and 4	19	6,865 *(56)*	17	6,093 *(55)*	7	1,054 *(32)*	4	1,240 *(26)*	4	1,203 *(36)*	3	1,031 *(31)*
≥60% cases with stage 3 and 4	12	1,148 *(9)*	12	1,140 *(10)*	7	572 *(17)*	9	941 *(20)*	6	628 *(19)*	4	364 *(11)*
*Not known*	*23*	*4,271 (35)*	*19*	*3,780 (34)*	*13*	*1,698 (51)*	*13*	*2,556 (54)*	*10*	*1,479 (45)*	*9*	*1,912 (58)*
**Tumor grade**												
<40% cases with grade 3	30	6,675 *(54)*	28	6,593 *(60)*	18	2,415 *(73)*	3	302 *(6)*	2	223 *(7)*	1	163 *(5)*
≥40% cases with grade 3	15	1,861 *(15)*	12	1,448 *(13)*	7	831 *(25)*	14	2,686 *(57)*	12	2,388 *(72)*	11	2,102 *(64)*
*Not known*	*9*	*3,748 (31)*	*8*	*2,972 (27)*	*2*	*78 (2)*	*9*	*1,749 (37)*	*6*	*699 (21)*	*4*	*1,042 (32)*
**Storage conditions of Tumor tissue**												
Frozen	6	433 *(4)*	3	265 *(2)*	2	161 *(5)*	1	60 *(1)*	0	0 *(0)*	0	0 *(0)*
FFPE	25	5,974 *(49)*	24	5,284 *(48)*	19	2,369 *(71)*	15	2,059 *(43)*	13	1,747 *(53)*	10	1,408 *(43)*
Both	1	80 *(0.7)*	1	81 *(0.7)*	1	83 *(2)*	0	0 *(0)*	0	0 *(0)*	0	0 *(0)*
*Not known*	*22*	*5,797 (47)*	*20*	*5,383 (49)*	*5*	*711 (21)*	*10*	*2,618 (55)*	*7*	*1,563 (47)*	*6*	*1,899 (57)*
**Timing of Tumor tissue collection**												
Prospective	30	9,274 *(75)*	27	8,195 *(74)*	10	930 *(28)*	11	2,731 *(58)*	6	1,503 *(45)*	6	1,899 *(57)*
Retrospective (archival material)	17	2,546 *(21)*	16	2,477 *(22)*	14	2,183 *(66)*	15	2,006 *(42)*	14	1,807 *(55)*	10	1,408 *(43)*
*Not known*	*7*	*464 (4)*	*5*	*341 (3)*	*3*	*211 (6)*	*0*	*0 (0)*	*0*	*0 (0)*	*0*	*0 (0)*

a Percentage of the number given in the total row (percentages for each variable do not always add to 100 because of rounding errors).

b Includes four studies from Morocco (number of women with known ER, PR, and HER2 status: 1,451, 1,451, and 411, respectively), three from Libya (378, 378, 78, respectively), two from Sudan (162, 162, and 114, respectively), and two from Algeria (296, 296, and 296, respectively) (see Tables 1 and 2).

c Includes a multi-centric study [8] with several centres based in Nigeria and one in Senegal (number of women with known ER, PR, and HER2 status: 378, 378, and 378, respectively).

d Includes two studies from Mali (number of women with known ER, PR, and HER2 status: 273, 272, and 113, respectively); four from Ghana (320, 293, and 258, respectively); two from Uganda (296, 183 and 183, respectively), two from Kenya (278, 158 and 192, respectively), two from Tanzania (125, 125 and 0, respectively), and one from Madagascar (75 and 77, respectively) (see Tables 1 and 2).

e Defined according to the last year in which patient recruitment took place.

f If information on menopausal status was not available women aged >50 years at diagnosis were classified as postmenopausal.

Figure 3 shows study-specific reported proportions of ER+, PR+, and HER2+ tumors, ranked according to their magnitude, for North and sub-Saharan Africa. There was marked between-study heterogeneity in the ER+ estimates in both regions (I^2^>90%), with the majority reporting proportions between 0.40 and 0.80 in North Africa and between 0.20 and 0.70 in sub-Saharan Africa. Similarly, large between-study heterogeneity was observed for PR+ and HER2+ estimates (I^2^>80%, in all instances). There were no clear differences in the reported proportions of HER2+ tumors according to whether they were classified with a IHC cut-off score of 2+/3+ or 3+ as HER2+, or whether they were, or were not, further tested with FISH, CISH, or SISH.

### Between-Study Heterogeneity

#### Study design

**Figure 4:**
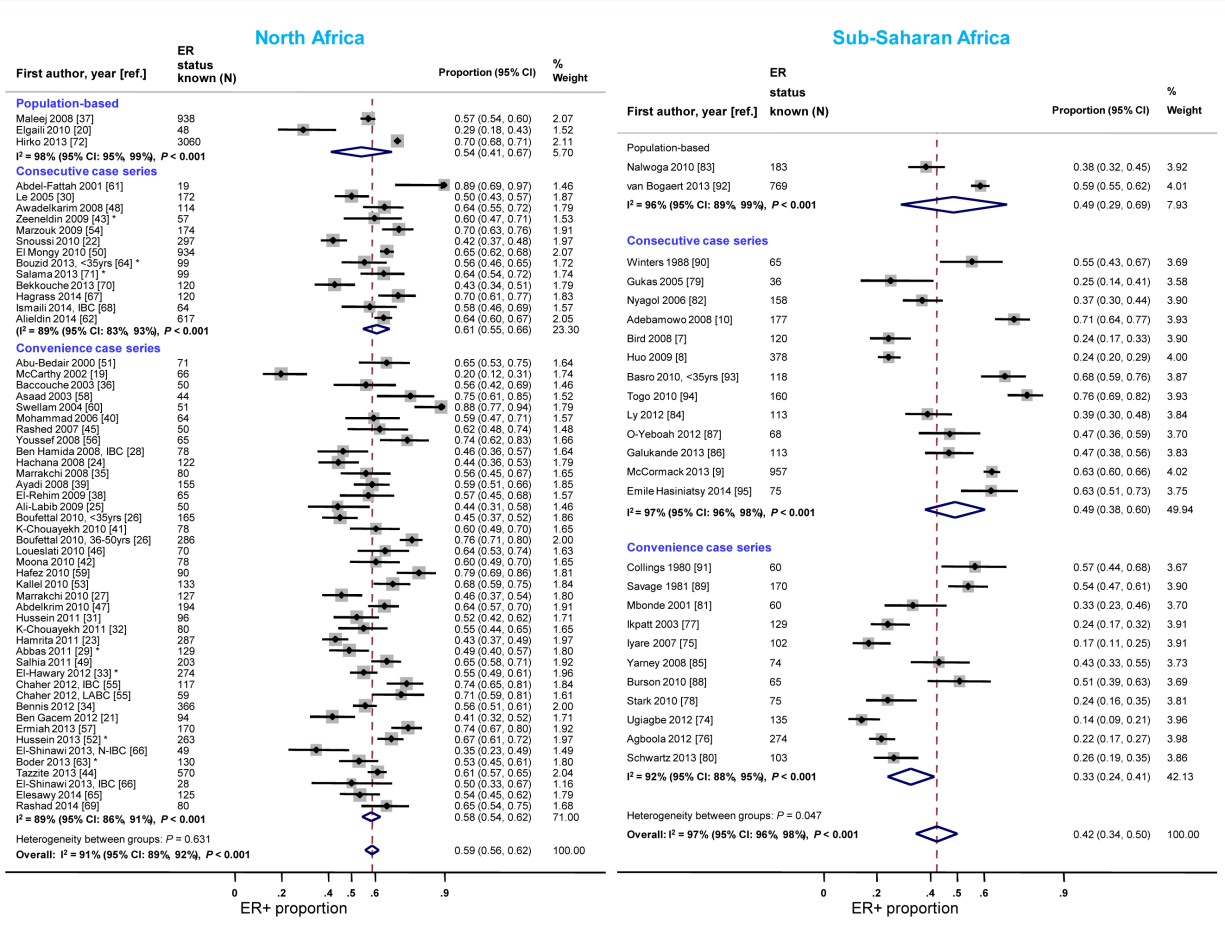
Proportion of ER+ disease by study design, North and sub-Saharan Africa. IBC, inflammatory breast cancer; LABC, non-IBC locally advanced breast cancer; N-IBC, non-inflammatory breast cancer. *These studies provided only a combined HR estimate for tumors that were either ER+ or PR+ [33] or ER+ and/or PR+ ([29]; [43]; [52]; [63]; [64]; [71]).

Case series based on convenience samples predominated in North Africa whereas roughly half of the case series in sub-Saharan Africa were consecutive (Table 3). For North African studies, there were no consistent differences in the ER+ proportion by study design; for sub-Saharan African studies, the studies that yielded the highest ER+ estimates tended to be those based on population-based or consecutive series rather than those based on convenience samples but there was still wide between-study variability among the former (Figure 4). A similar pattern was observed for PR receptor status (Figure S1). There were no clear differences by study design for HER2 status in North or sub-Saharan Africa (Figure S2).

#### Year of diagnosis

**Figure 5:**
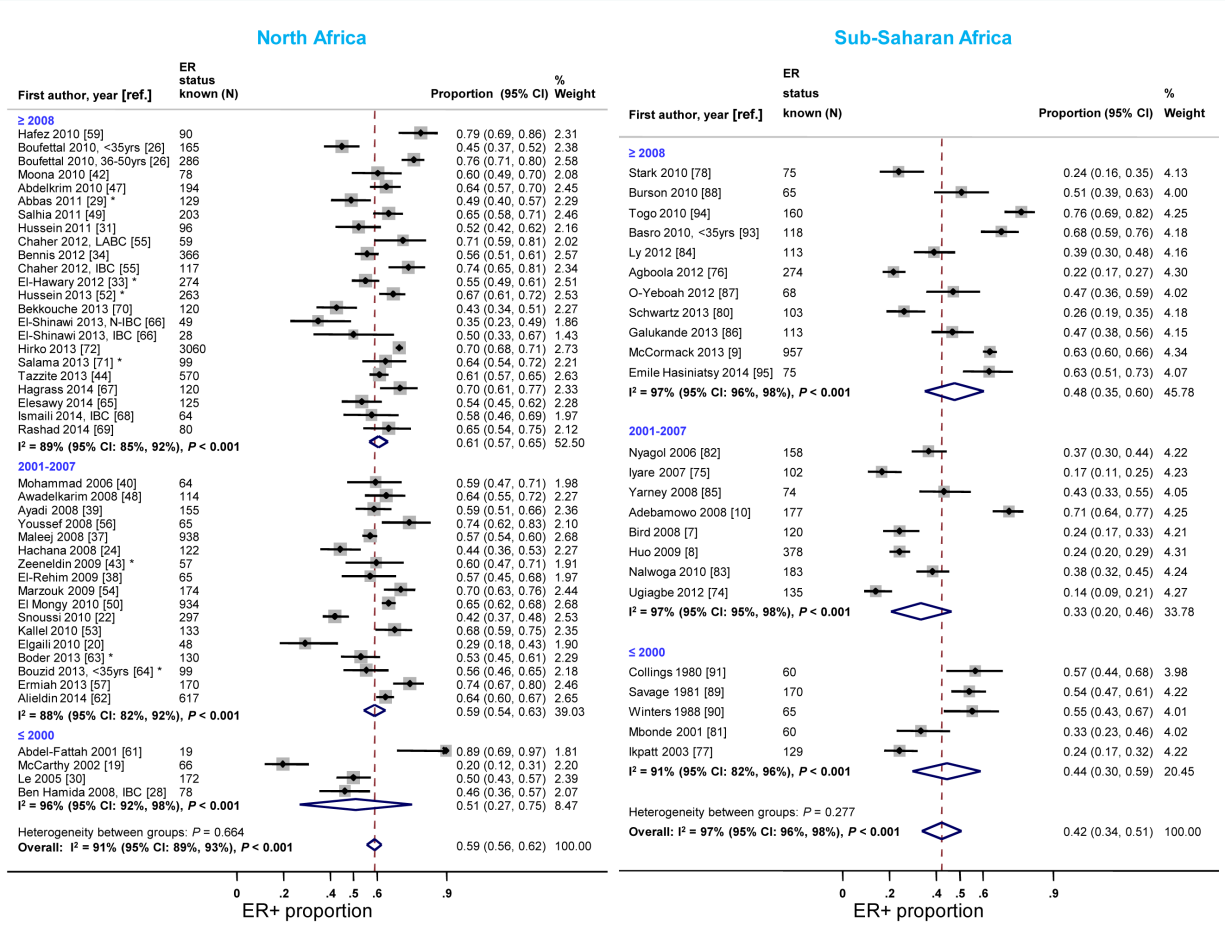
Proportion of ER+ disease by year of diagnosis, North and sub-Saharan Africa. IBC: inflammatory breast cancer; LABC: non-IBC locally advanced breast cancer; N-IBC: non-inflammatory breast cancer. *These studies provided only a combined HR estimate for tumors that were either ER+ or PR+ [33] or ER+ and/or PR+ ([29]; [43]; [52]; [63]; [64]; [71]).

The majority of studies in both North and sub-Saharan Africa comprised women diagnosed with breast cancer after 2001 (Table 3). In each region, the study-specific ER+ proportion tended to increase over time. In North Africa, the rise was particularly noticeable when studies conducted before 2001 were compared to those completed after 2007 (Figure 5). An exception to this trend in sub-Saharan Africa was the generally higher ER+ proportion for studies conducted prior to 2001, driven by estimates from three South African studies [89]–[91], than for those conducted between 2001 and 2007. Similar increases over time in the proportion of PR+ disease were observed (Figure S3). In contrast, there was a slight decrease over time in the reported study-specific HER2+ proportion in North Africa; no sub-Saharan African study conducted prior to 2001 reported on HER2 status (Figure S4).

#### Age and menopausal status at diagnosis

Study-specific proportions of ER+ disease tended to increase with increasing average (mean/median) age at breast cancer diagnosis in both North and sub-Saharan Africa (e.g., pooled ER+ *prop* [95% CI] for sub-Saharan studies with an average age at diagnosis of 31–46, 47–49.4, and 49.5+ years were 0.34 [0.24–0.44], 0.45 [0.28–0.62], and 0.49 [0.35–0.64]; I^2^>90%, *p*<0.01 for all). A similar age pattern was observed for the proportion of PR+ disease in both regions. No clear age trends were observed for HER2+ disease (e.g., pooled HER2+ *prop* [95% CI] for North African studies with an average age at diagnosis of 31–46, 47–49.4, and 49.5+ years were 0.31 [0.27–0.36], 0.32 [0.22–0.43], and 0.30 [0.24–0.36]; I^2^>70%, *p*≤0.01 for all except ages 31–46 for which I^2^ = 15%, *p* = 0.32). There were no clear differences in the frequency of ER+, PR+, and HER2+ disease by menopausal status, but few studies (two in North Africa; four in sub-Saharan Africa) were based on case series where ≥60% of the women were postmenopausal at breast cancer diagnosis (Table 3).

#### Tumor grade and stage

**Figure 6:**
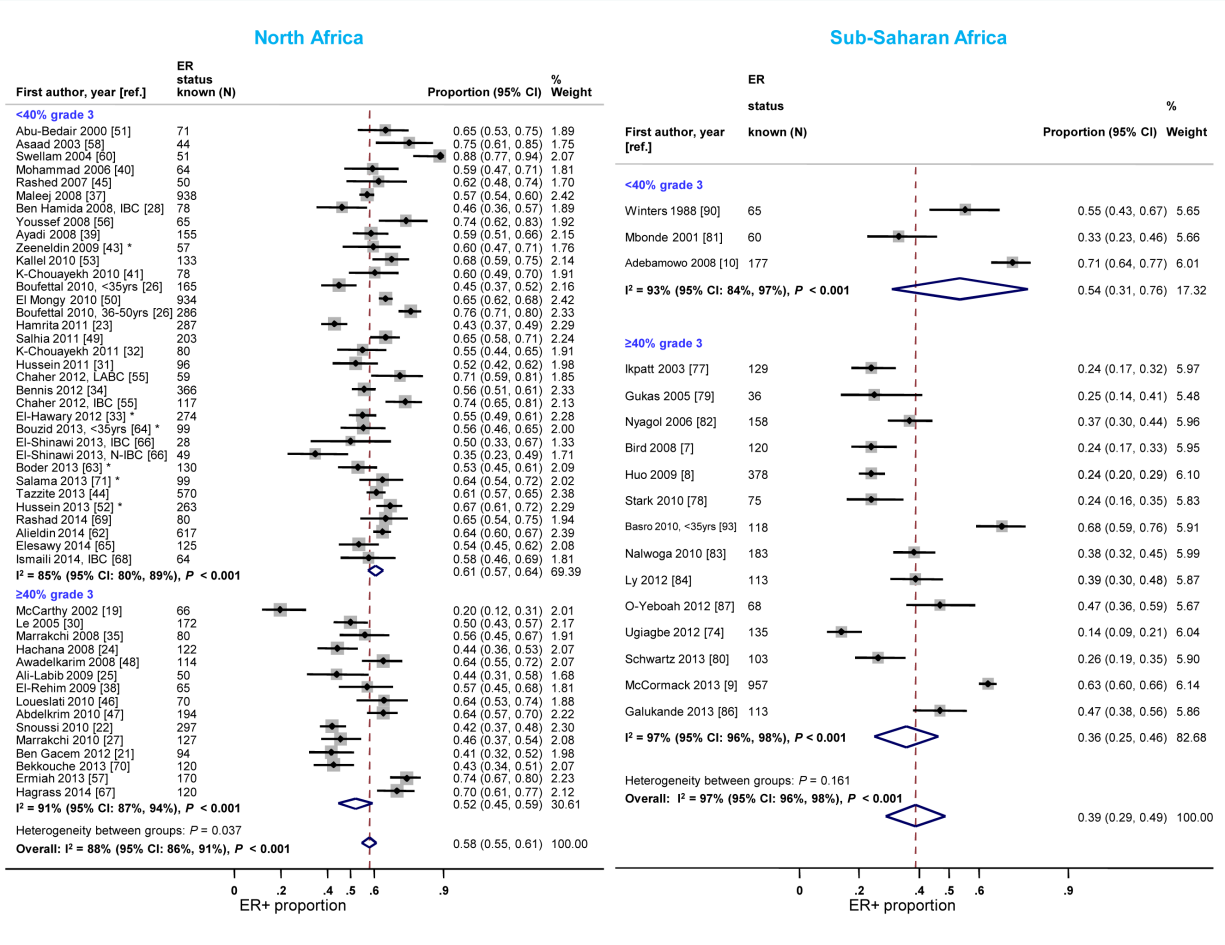
Proportion of ER+ disease by tumor grade, North and sub-Saharan Africa. IBC, inflammatory breast cancer; LABC, non-IBC locally advanced breast cancer; N-IBC: non-inflammatory breast cancer. *These studies provided only a combined HR estimate for tumors that were either ER+ or PR+ [33] or ER+ and/or PR+ ([43]; [52]; [63]; [64]; [71]).

North African studies with ≥40% grade 3 tumors reported a lower proportion of ER+ disease relative to those with <40% of such tumors (Figure 6). A similar gradient was observed in sub-Saharan Africa; however, only three studies had <40% grade 3 tumors (Figure 6; Table 3), reflecting perhaps their late presentation. Twelve studies [7],[9],[10],[33],[34],[39],[41],[51],[65],[77],[81],[86] provided grade-specific ER+ estimates and they all consistently showed decreasing ER+ proportions with increasing grade (Figure S5). There were no notable differences in the frequency of PR+ and HER2+ tumors by grade in North Africa; the paucity of studies with <40% of grade 3 tumors in sub-Saharan Africa precluded the examination of this variable (Figures S6 and S7). There were no consistent differences in receptor status by tumor stage.

#### Timing of receptor testing and specimen storage conditions

**Figure 7:**
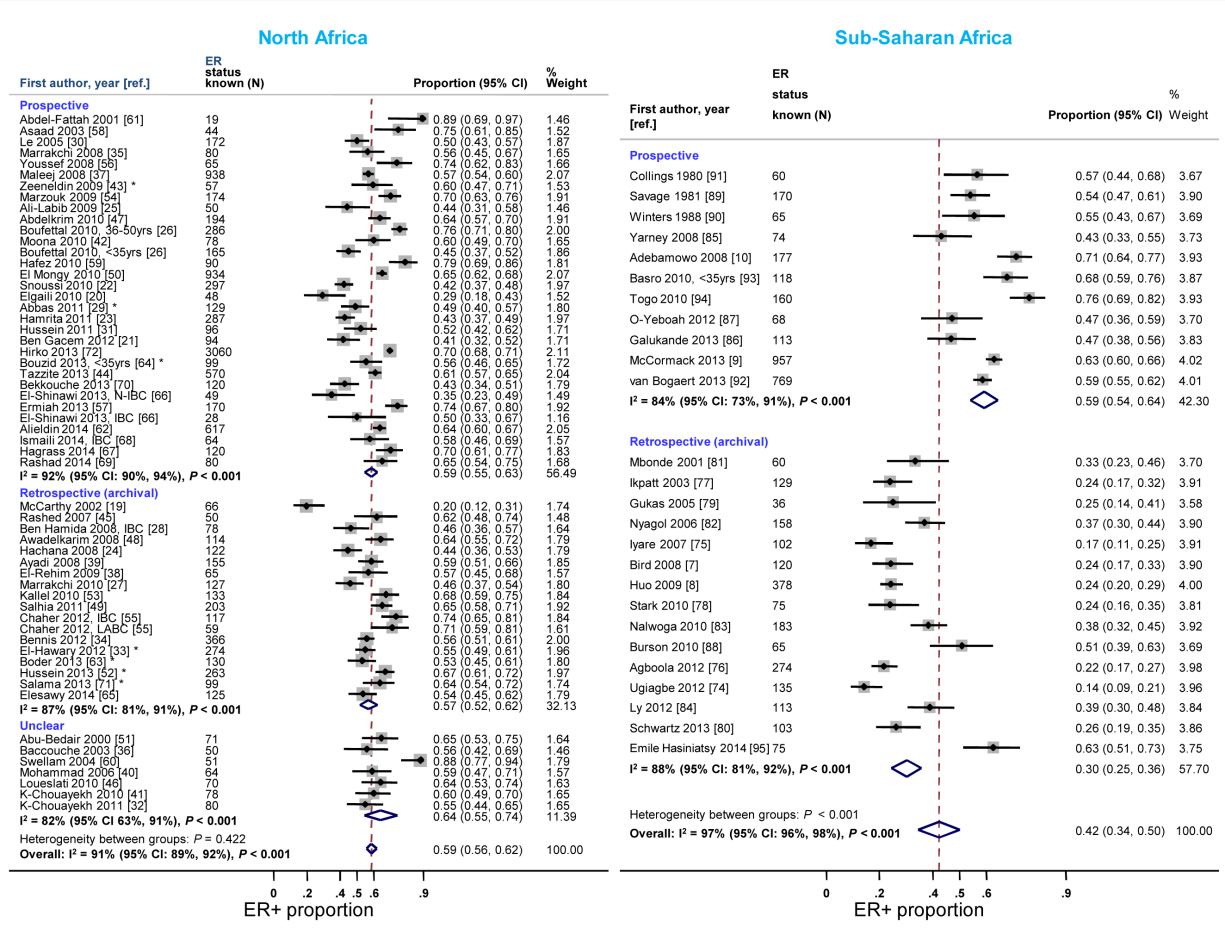
Proportion of ER+ disease by timing of receptor testing, North and sub-Saharan Africa. IBC, inflammatory breast cancer; LABC, non-IBC locally advanced breast cancer; N-IBC, non-inflammatory breast cancer. *These studies provided only a combined HR estimate for tumors that were either ER+ or PR+ [33] or ER+ and/or PR+ ([29]; [43]; [52]; [63]; [64]; [71]).

Reported proportions of ER+ and PR+ disease tended to be lower for studies where receptor status assays were conducted on retrospective (archival) tissue blocks than for those based on prospectively analysed specimens in sub-Saharan Africa, but not in North Africa (Figures 7 and S8). North African studies that used FFPE blocks tended to report lower ER+ (pooled prop = 0.57, 95% CI 0.52–0.62; I^2^ = 91%; *p*<0.01) and PR+ estimates (pooled *prop* = 0.51, 95% CI 0.46–0.55; I^2^ = 88%; *p*<0.01) than those based on frozen tissue samples (pooled ER+ prop = 0.64, 95% CI 0.52–0.76; I^2^ = 87%, *p*<0.01; pooled PR+ *prop* = 0.61; 95% CI 0.55–0.67; I^2^ = 0%; *p* = 0.88). Virtually all sub-Saharan African studies were based on FFPE tissue blocks (Table 3). No clear patterns in the frequency of HER2+ tumors by timing of receptor testing, or specimen storage conditions, were observed within each region (e.g., pooled *prop* [95% CI] for prospectively collected versus archival tissue: 0.36 [0.30–0.42] versus 0.28 [0.23–0.33] in North Africa; 0.22 [0.14–0.31] versus 0.20 [0.15–0.25] in sub-Saharan Africa [I^2^≥74% for all]; Figure S9).

#### Study quality

The median (inter-quartile range [IQR]) quality scores for studies reporting on ER, PR, and HER2 status for North Africa were 16 (14–17), 16 (15–18), and 15 (14–17), respectively (Table 1). The corresponding estimates for sub-Saharan Africa were 17 (15–19), 17 (15–19), and 16 (14–18) (Table 2). There were no clear differences in the frequency of ER+, PR+, and HER2+ disease by study quality scores, despite the differences observed for specific individual criteria (e.g., study tissue storage conditions, timing of receptor testing) described above.

#### Geographical sub-regions

**Figure 8:**
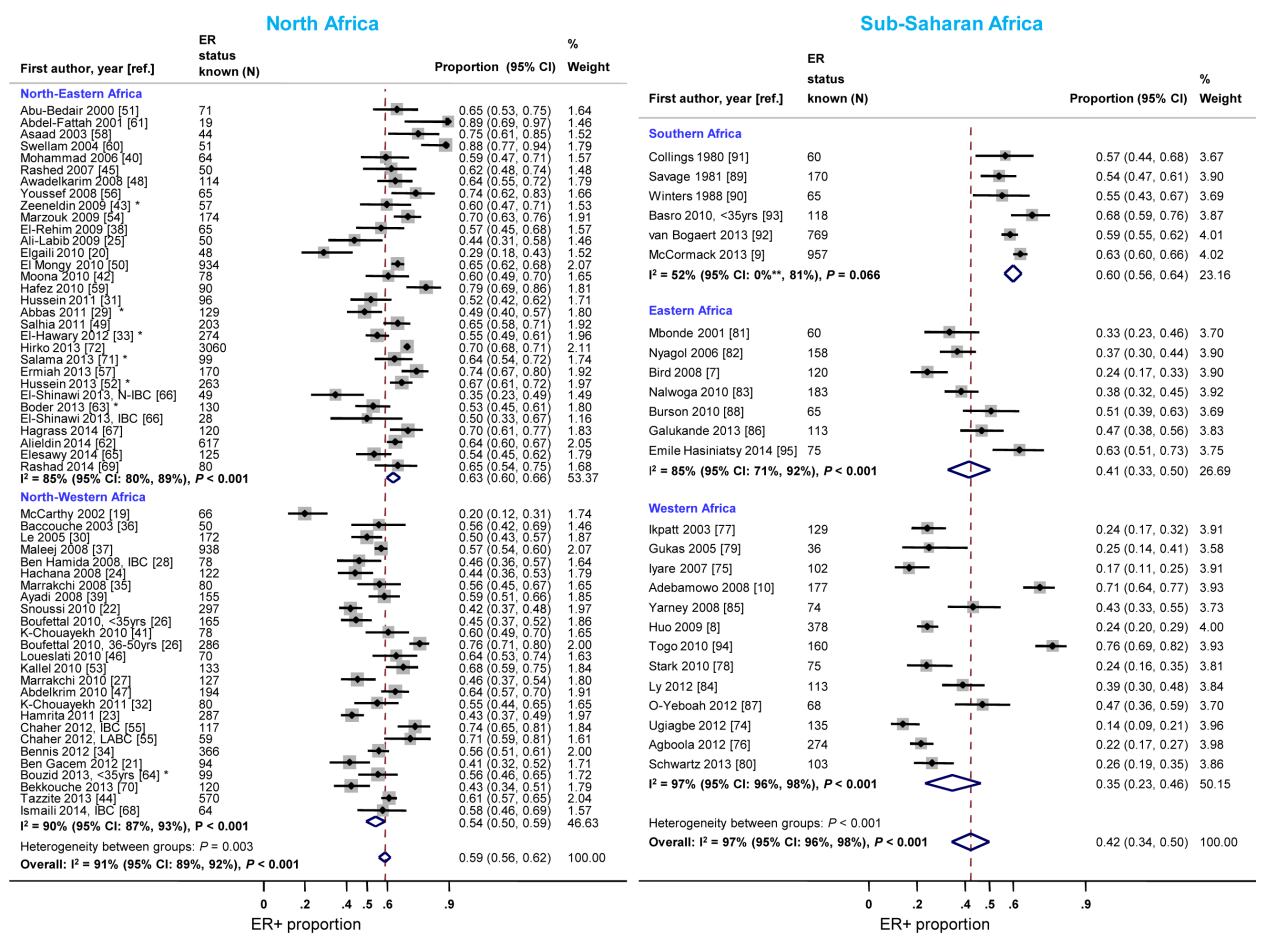
Proportion of ER+ disease by sub-regions within North and sub-Saharan Africa. IBC, inflammatory breast cancer; LABC, non-IBC locally advanced breast cancer; N-IBC, non-inflammatory breast cancer. North-Western Africa: Morocco, Algeria, and Tunisia; North-Eastern Africa: Egypt, Sudan, and Libya; Eastern Africa: Kenya, Uganda, Tanzania, and Madagascar; Western Africa: Ghana, Mali, Nigeria, and Senegal; Sothern Africa: South Africa. *These studies provided only a combined HR estimate for tumors that were either ER+ or PR+ [33] or ER+ and/or PR+ ([29]; [43]; [52]; [63]; [64]; [71]). **Lower limit of 95% confidence interval for I^2^ statistic truncated at 0.

Studies from North-Eastern Africa (i.e., Egypt, Sudan, and Libya) yielded higher ER+ proportions than those conducted in North-Western Africa (i.e., Morocco, Algeria, and Tunisia) (Figure 8). There was also a gradient within sub-Saharan Africa with the highest ER+ proportions being reported by studies from Southern Africa (i.e., South Africa) and the lowest by studies from Eastern Africa (i.e., Kenya, Uganda, Tanzania, and Madagascar) and Western Africa (i.e., Ghana, Mali, Nigeria, and Senegal) (Figure 8). Similar patterns by sub-region were observed for PR+ disease except that the gradient within North Africa was smaller (Figure S10). There was no variation in the frequency of HER2+ disease between the two North African sub-regions but, similarly to ER+ and PR+ disease, the proportion of HER2+ disease was highest for studies from Southern Africa and lowest for those from Western Africa (Figure S11).

#### Meta-regression analyses

Adjusted meta-regression analyses (Table 4) showed that the reported proportion of ER+ disease was 10% (95% CI 4%–17%) lower for studies based on archived tumor blocks versus those based on prospectively collected specimens, and 9% (2%–17%) lower for those with ≥40% versus those with <40% grade 3 tumors. The reported ER+ proportion was also higher for North African than sub-Saharan studies, but only among studies based on retrospective (archival) samples (*p* for interaction between region and time of receptor testing: <0.001). Similarly, further breakdown by sub-region showed that relative to North-Western Africa, the ER+ proportion was higher for North-Eastern (8.5%; 95% CI 1%–16%) and Southern Africa (5%; −8% to 18%), but lower for Western (−18%; −28% to −8%) and Eastern Africa (−11%; −24% to 1%). There was, however, an interaction with timing of receptor testing (*p* = 0.0001), with no differences in the ER+ proportion between sub-regions being observed among studies based on prospectively collected samples. There was a tendency for the proportion of ER+ disease to increase with increasing age and year at diagnosis. Similar patterns were observed for proportion of PR+ disease. The patterns for HER2+ were less clear but the reported proportions tended to be slightly higher for studies based on prospectively collected specimens, those conducted before 2001, and those from North Africa regardless of the timing of receptor testing (Table 4).

**Table 4:** Sources of between-study heterogeneity in the proportions of ER+, PR+, and HER2+ cases from meta-regression analyses.

Variable	ER+					PR+					HER2+				
	*N*	Crude		Adjusted^a^		*N*	Crude		Adjusted^a^		*N*	Crude		Adjusted^a^	
		Absolute Difference (%)	(95% CI)	Absolute Difference (%)	(95% CI)		Absolute Difference (%)	(95% CI)	Absolute Difference (%)	(95% CI)		Absolute Difference (%)	(95% CI)	Absolute Difference (%)	(95% CI)
**Year of diagnosis** b **^,^** c															
≤2000	9	−3.4	(−16.4 to 9.6)	−0.19	(−11.4 to 11.0)	5	−14.0	(−31.6 to 3.5)	−10.8	(−25.6 to 3.9)	2	9.4	(−9.1 to 28.0)	3.0	(−16.6 to 22.6)
2001–2007	25	0 (ref)		0 (ref)		23	0 (ref)		0 (ref)		14	0 (ref)		0 (ref)	
≥2008	34	5.8	(−3.0 to 14.5)	4.7	(−2.7 to 12.2)	32	4.1	(−5.2 to 13.4)	3.9	(−3.9 to 11.6)	23	0.3	(−7.7 to 8.3)	−2.7	(−10.5 to 5.0)
**Age at diagnosis (y)** b **^,^** d															
31–	21	0 (ref)		0 (ref)		18	0 (ref)		0 (ref)		11	0 (ref)		0 (ref)	
47–	24	9.7	(−0.0 to 19.4)	5.4	(−3.2 to 14.1)	22	8.4	(−2.6 to 19.5)	3.8	(−5.7 to 13.3)	10	−3.0	(−13.5 to 7.6)	−6.1	(−16.9 to 4.6)
49.5+	25	13.0	(3.4–22.6)	6.7	(−2.08 to 15.4)	22	9.1	(−1.9 to 20.0)	0.1	(−9.5 to 9.6)	18	2.0	(−7.1 to 11.1)	−4.7	(−15.4 to 6.0)
**Grade 3** b															
<40%	37	0 (ref)		0 (ref)		34	0 (ref)		0 (ref)		22	0 (ref)		0 (ref)	
≥40%	29	−15.9	(−23.4 to −8.3)	−9.1	(−16.6 to −1.5)	24	−12.2	(−21.1 to −3.4)	−2.5	(−11.2 to 6.3)	18	−2.0	(−9.6 to 5.6)	4.4	(−4.6 to 13.4)
**Timing of receptor testing**															
Prospective	43	0 (ref)		0 (ref)		35	0 (ref)		0 (ref)		17	0 (ref)		0 (ref)	
Retrospective	33	−13.6	(−20.6 to −6.6)	−10.4	(−17.3 to −3.6)	31	−12.3	(−20.4 to −4.3)	−5.6	(−13.1 to 2.0)	25	−6.0	(−13.3 to 1.3)	−8.3	(−15.7 to −0.9)
Unclear	7	5.7	(−7.0 to 18.3)	4.2	(−10.2 to 18.6)	5	5.9	(−10.0 to 21.8)	4.3	(−12.1 to 20.8)	3	−5.5	(−20.2 to 9.3)	−6.5	(−22.8 to 9.9)
**Region**															
Sub-Saharan Africa	26	0 (ref)		0 (ref)		20	0 (ref)		0 (ref)		16	0 (ref)		0 (ref)	
North Africa	57^e^	16.4	(9.3–23.4)	12.3	(5.1–19.6)	51 e	21.4	(13.8–29.0)	20.0	(11.5–28.4)	29 e	9.3	(2.6–16.0)	10.8	(2.4–19.1)
*Stratified by timing of receptor testing* f															
*Prospective*															
Sub-Saharan Africa	11	0 (ref)		0 (ref)		6	0 (ref)		0 (ref)		6	0 (ref)		0 (ref)	
North Africa	32	−0.1	(−9.3 to 9.1)	0.4	(−9.9 to 10.6)	29	10.5	(−3.9 to 24.9)	14.8	(−0.3 to 29.9)	11	13.6	(2.0–25.2)	12.2	(−1.9 to 26.3)
*Retrospective*															
Sub-Saharan Africa	15	0 (ref)		0 (ref)		14	0 (ref)		0 (ref)		10	0 (ref)		0 (ref)	
North Africa	18	26.7	(17.7–35.7)	23.6	(14.2–33.1)	17	24.1	(15.3–32.9)	22.3	(10.6–34.0)	15	7.5	(−1.1 to 16.0)	7.4	(−7.1 to 21.9)

a Adjusted for all other variables in the table except tumor grade (this variable was not included in the model because of potential for over-adjustment). The variable study design was not included in the final models because it was not associated with the frequency of ER+, PR+, or HER2+ in the crude or adjusted analyses.

b Missing values were included as separate categories.

c Defined according to the last year in which patient recruitment took place.

d Mean or median age of study cases at the time of breast cancer diagnosis; for studies that provided only age categories the mean was estimated from the mid-point and frequency of each category.

e These numbers are higher than the total number of North African studies included in the review (Table 3) because one study [26] presented separate ER+ and PR+ estimates for ages <35 y and 36–50 y and another [55] presented separate ER+, PR+, and HER2+ estimates for inflammatory (IBC) and non-IBC locally advanced breast cancer (LABC).

f *p*-values for interaction between region and timing of receptor testing for ER+: *p*<0.001 in the crude analysis, *p*<0.001 in the adjusted analysis; PR+: *p* = 0.10 in the crude analysis, *p* = 0.17 in the adjusted analysis; HER2+: *p* = 0.37 in the crude analysis, *p* = 0.52 in the adjusted analysis.

### Combined ER/PR/HER2 Tumor Subtypes

**Figure 9:**
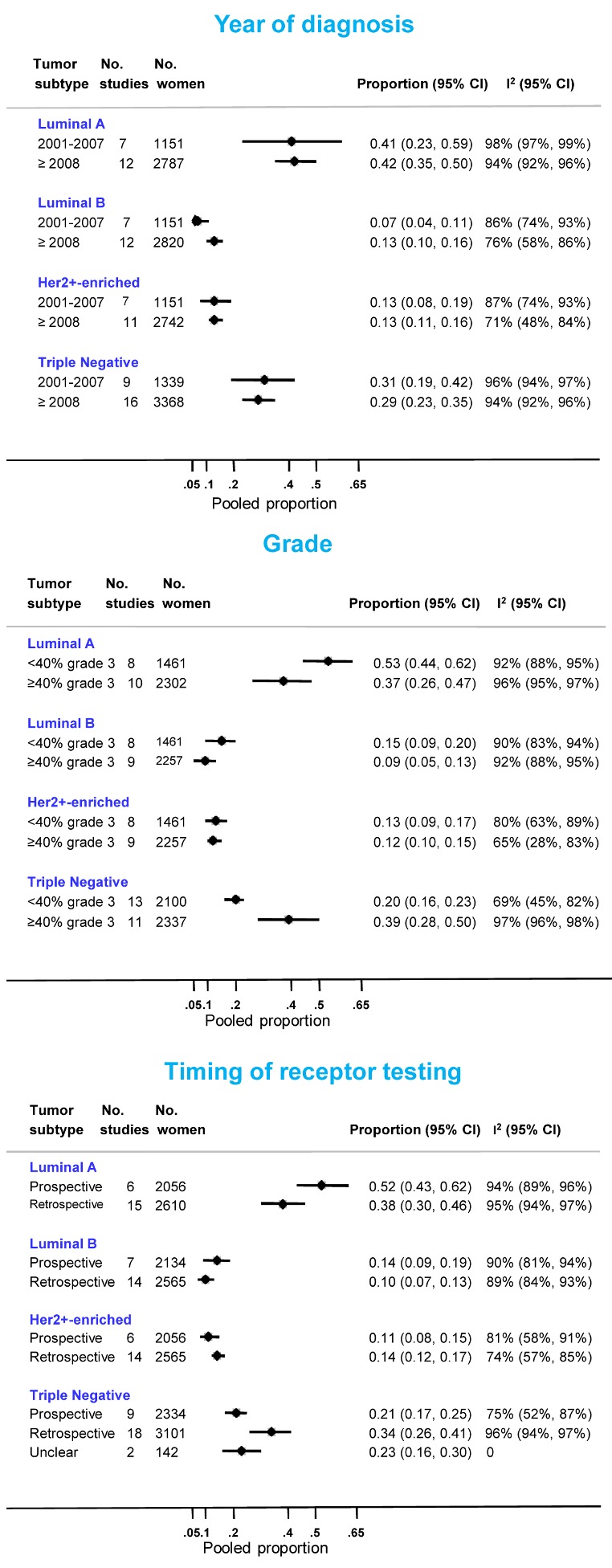
Frequency of tumor subtypes by year of diagnosis, grade, and timing of receptor testing.

Eighteen North African [21],[32]–[34],[39],[41],[42],[47]–[49],[52],[55],[56],[65],[69]–[71] and 12 sub-Saharan African studies [7]–[10],[75],[76],[78],[80],[82]–[84],[92] provided information on the frequency of one or more subtypes. Consistent with the findings reported above, the proportion of triple negative tumors was lower for studies based on prospectively collected samples and those with <40% grade 3 tumors (Figure 9). The opposite was true for luminal A and, to a lesser extent, luminal B tumors. In contrast, there was little variation in the frequency HER2+-enriched tumors according to these two variables. However, marked between-study heterogeneity was still present within each stratum (Figure 9).

### International and Ethnic Comparisons

**Figure 10:**
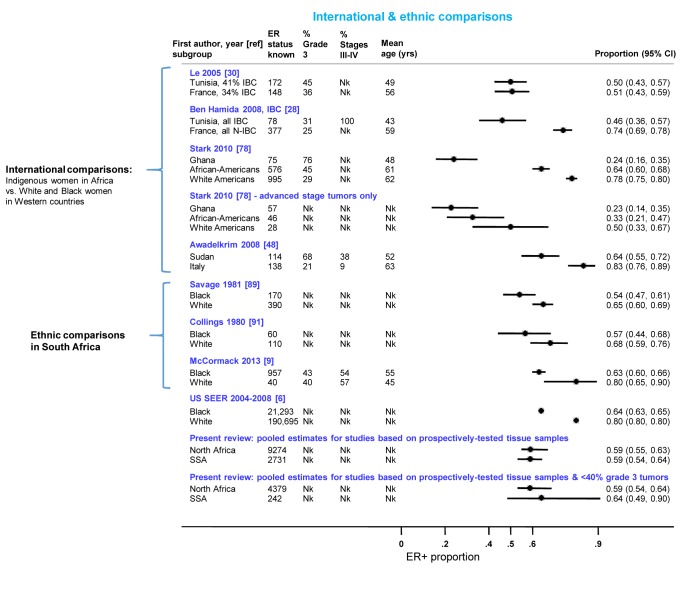
International and ethnic comparisons in the proportion of ER+ disease. IBC, inflammatory breast cancer; N-IBC, non-inflammatory breast cancer; Nk, information not given in the original paper; SEER, Surveillance, Epidemiology, and End Results (SEER) Program of the National Cancer Institute, US (data downloaded from [6] using the same methods as in [9]); SSA, sub-Saharan Africa.

Figure 10 presents the findings from studies that involved international or ethnic comparisons. The international comparisons highlighted the striking differences between indigenous African and Western white women with breast cancer, with the former showing a much younger age as well as larger tumor sizes and higher grade and stage, consistent with a more advanced disease at presentation. Despite these differences, Le and colleagues [30] reported similarly low proportions (∼0.50) of ER+ disease among both Tunisian and French women with breast cancer (the two series were selected to ensure they had broadly similar percentages of inflammatory breast cancers (T4d) Figure 10). In contrast, Ben Hamida and colleagues (Figure 10) [28] reported a higher proportion of ER+ disease among French (0.74) relative to Tunisian (0.46) patients; however, all Tunisian tumors, but none of the French ones, were inflammatory breast cancers. Stark and colleagues [78] reported large differences in the proportion of ER+ disease between Ghanaian (0.24), African-American (0.64), and white American (0.78) women; however, the differences were far less marked when the analysis was restricted to advanced stage disease (Figure 10). Awadelkrim and colleagues [48] reported a ER+ proportion of 0.64 among Sudanese women versus a proportion of 0.83 among Italian women, but the proportion of advanced tumors was much higher for the former (Figure 10). Three studies from South Africa [9],[89],[91], presented remarkably consistent between-ethnic differences despite covering a 30-year period, with all reporting smaller differences in the frequency of ER+ disease between black and white women than those described above (Figure 10), with this magnitude being broadly in line with the magnitude of the ethnic differences between black and white women in the Surveillance, Epidemiology, and End Results Program of the National Cancer Institute, US (SEER) data (data downloaded from [6] using the same methods as in [9]) (Figure 10). The pooled proportions of ER+ disease yielded by this review for North (0.59) and sub-Saharan studies (0.59) on the basis of the possibly better quality prospectively collected samples, were broadly similar to the ER+ proportion for US black women in the SEER data (0.64). Notably, when the analysis was further restricted to studies in this review with <40% grade 3 tumors, a case mix more akin to that seen in the US series, the pooled ER+ proportions for North (0.59; 95% CI 0.54–0.64) and sub-Saharan studies (0.64; 0.49–0.90; based on two studies) were similar to the ER+ proportion seen among US black women (0.64) (Figure 10).

### Small Study Bias

The funnel plots (Figure S12) and Egger's test for small study effects provide evidence of small study bias for North African studies only (*p*-values for studies reporting on ER, PR, and HER2 status: *p* = 0.004, 0.03, and 0.01, respectively).

## Discussion

### Main Findings

This systematic review aimed to characterize the distribution of receptor-defined subtypes of breast cancer in indigenous populations in Africa. It highlighted the extent to which data on these receptors, which are important prognostic markers of the disease, is scarce in the continent. Nevertheless, we identified 80 studies, comprising >17,000 women with breast cancer, with information on at least ER status, thus providing the largest synthesis so far to our knowledge of breast cancer subtypes in Africa. The review revealed large between-study heterogeneity in the reported frequency of ER+ tumors, ranging approximately from 1 in 4 to 3 in 4 tumors being ER+ within each region. This heterogeneity may have arisen as a result of regional and temporal differences in the prevalence of subtype-specific risk factors, differences in tumor characteristics (e.g., grade, stage) at presentation, or artefacts caused by unrepresentative case series and varying quality in the procedures used to collect, store, and analyse tumor specimens.

The review revealed a tendency for studies based on archival tissue and/or FFPE blocks to yield lower ER+ and PR+ frequency estimates, in line with archival samples being particularly susceptible to antigen degradation [96],[97]. Additionally, such archival samples tended to be from older studies where quality control on pre-analytical factors may have been suboptimal. More recent studies have demonstrated the vulnerability of hormone receptor (HR) testing to false negatives and the importance of pre-analytic factors, with errors introduced by delays, inadequate or prolonged fixation and variability in fixatives used, dehydration procedures, and quality of paraffin. The present review also found that the proportion of ER+ disease decreased with increasing tumor grade, reflecting perhaps the accelerated growth rate of ER− tumors, loss of estrogen expression in more advanced forms of the disease, and higher likelihood of false-negative results (due to difficulties in obtaining a biopsy of the original tumor). Although the observed increase in the frequency of ER+ disease over time may reflect improvements in methodology as well as the change in the tumor nuclei staining intensity score threshold for ER positivity from ≥10% to ≥1% (following the introduction of new guidelines in 2010 [98]), they may also represent a genuine rise in ER+ disease as African women became more westernised (as illustrated by declines in fertility [99] and rises in body mass index [100],[101] and, consequently, age at menarche in the continent).

A few studies in this review included international or ethnic comparisons in the distribution of ER status. None of the international studies appeared to have conducted centralized receptor status testing, with none reporting on cross-centre evaluation of comparability in measurements and quality control procedures, but each one of the three ethnic studies was conducted within a single institution and hence using the same procedures for all their participants. These comparisons consistently reported a lower frequency of ER+ tumours in indigenous women in Africa relative to Western white women, or in black relative to white women in South Africa, consistent with the well documented ethnic differences in the US. The existence of, and reasons for, the black-white differences in the US may shed light on the situation in Africa. Over age 35 years, a higher ER+ proportion among US white than black women with breast cancer is driven by the latter group's slightly higher absolute incidence rate of triple negative disease, in combination with their much lower incidence rate of better prognosis ER+/PR+ HER2− tumors [102]. However, the magnitude of the black-white difference in the ER+ proportion has changed somewhat over time and the reasons driving these differences are much debated [11]. As risk factors are subtype-specific, ethnic differences in the prevalence of hormonal-related risk factors may contribute to ethnic differences in the incidence of the various breast cancer subtypes. Pre-menopausal obesity and higher parity may be associated with raised risk of tripe-negative disease, in contrast to their protective effects on ER+ disease [103],[104], and oral contraceptive use may increase more markedly the risk of triple negative disease than the risk of other subtypes [105]. Equally, or in addition, ethnic differences may derive from genetic susceptibility to triple negative or ER-negative breast cancer in some African populations [106],[107].

In the present study, relative to breast cancer in Western white women, the disease in indigenous women in Africa was characterized by a younger age, an advanced stage, and a higher grade at presentation (Figure 10). Both young age and more advanced forms of the disease at presentation are associated with lower prevalence of ER+ tumors. Thus, the observed lower frequency of ER+ tumors in indigenous African women may simply reflect a much younger demographic structure of the indigenous African populations rather than a more intrinsic aggressive biology of the disease, as incidence rates at young ages are lower than among Western white women [1], as well as a tendency for late presentation due to lack of breast cancer awareness and screening activities, the unavailability of appropriate healthcare facilities, and the influence of socio-cultural and logistic factors that could limit access to health-care. In fact, our finding that the proportion of ER+ disease reported by African studies based on prospectively collected samples with predominantly low grade tumors was virtually the same as among US black women (all ∼64%) argues against breast cancer being a much more biologically aggressive disease in Africa than in the West.

Two subtypes are known to be associated with particularly poor breast cancer outcomes: triple negative and HER2+-enriched tumors. Few studies provided information on these subtypes and even fewer were based on prospectively collected samples. Nevertheless, the estimates based on the latter for triple negatives (pooled *prop* = 0.21; Figure 9) were slightly above the range of frequencies usually seen in white populations (10%–16%) [2],[108], but similar to that seen in US black women (e.g., 26% in [109]). The prevalence of HER2+-enriched tumors (pooled *prop* = 0.11) (Figure 9) was slightly higher than that seen in white populations [2] or US black women [109] (6%–10% for both) but similar to that reported for Chinese women [108]. However, considerable misclassification of HER2 status may have occurred as few African studies used FISH (or CISH/SISH) to ascertain the true HER2 status of tumors with an equivocal IHC score of 2+.

It is noteworthy to highlight that although between-study differences in the proportion of ER+ disease reflect the ratios of the underlying receptor-specific incidence rates (assuming no bias is present), they cannot be used to infer anything about the differences in incidence rates. The proportion of ER+ disease represents the ratio of the number of women who developed ER+ disease in a given population over a certain time period (thus, reflecting the underlying incidence rate of ER+ disease) by the total number of women who develop any type of breast cancer in the same population during the same time period (reflecting the incidence rate of ER+ and ER− disease combined). Thus, differences in the proportion of ER+ disease among women with breast cancer could arise from two populations with (i) the exact same incidence rates of ER− disease, but different incidence rates of ER+ disease, or (ii) equal incidence rates of ER+ disease, but different rates of ER− disease, or (iii) any combinations of these two. Case-only studies are unable to disentangle these different alternatives. Consequently, the findings from this review cannot be used to infer differences in the underlying incidence rates of receptor-specific disease across populations, e.g., between North and sub-Saharan Africa.

### Strengths and Limitations

Major strengths of this review are the very comprehensive and inclusive search strategy (with inclusion of African-specific journals, the use of broad search terms rather than specific keywords, and the decision not to impose any language restrictions), the large number of eligible studies (comprising >17,000 women with breast cancer), and the use of well-established methodologies to provide an unbiased synthesis of the published evidence. The study had several weaknesses too. Firstly, the systematic review includes data from all countries in North Africa except Western Sahara, but with a predominance of studies from Egypt and Tunisia (Table 3). The proportion of sub-Saharan countries represented in the review was much smaller—only nine (i.e., South Africa, Nigeria, Senegal, Mali, Ghana, Uganda, Kenya, Tanzania, and Madagascar) out of 49 countries, albeit together these countries represent 46% of the total African female population [1]. Furthermore, no receptor status testing is performed in many of the countries not represented in the review. Secondly, the representativeness of the case series was not only compromised by the poor design of many of the participating studies, particularly those based on convenience samples, but also by the limited access to appropriate diagnostic and treatment facilities experienced by most indigenous African women affected with breast cancer. For instance, in many countries, receptor status testing in public hospital attendees is only available to those who can afford it. Thirdly, it is also possible that women with breast cancer may have contributed to more than one study. When multiple papers from the same study were identified, only the one with the most information on receptor status was included in the review. However, it was often impossible to ascertain potential overlaps in study populations, particularly among studies conducted within the same institution. This was a particular issue for Egyptian and Tunisian studies published in the early years, most of which provided a poor description of how their study populations were recruited, but sensitivity analyses including only studies in each institution whose recruitment dates did not overlap yielded similar estimates to those reported here. Fourthly, there was no suggestion that small study bias affected the results for receptor status in sub-Saharan Africa, but for North African studies, the smaller studies tended to have lower-than-average ER+ and PR+ proportions and higher-than-average HER2+ proportions. If this small study bias is real, the true ER+ and PR+ proportions would be higher and the HER2+ lower than the pooled estimates reported here. Finally, real geographical or temporal differences in the frequency of breast cancer subtypes may have been obscured by the lack of standardisation in pre-analytical and analytical procedures across studies.

### Implications

Large well-designed studies, incorporating standardised high-quality procedures for receptor testing, are required to accurately quantify the distribution of the various breast cancer subtypes across Africa. In the meantime, this systematic review provides the strongest evidence yet that the distribution of receptor-defined subtypes is not dramatically different to that found in Western populations given their younger age structure and late presentation. The availability of receptor testing should be a priority in Africa, especially for young women with early stage disease where the potential to improve survival and reduce years of life-lost is greatest. In the absence of such testing, it would be appropriate to presume that the majority of tumors are ER+.

The findings have important implications for both research needs and public health in Africa. In addition to the need for high-quality characterisation of receptor-status, etiologic studies on breast cancer in the continent need to be conducted separately by subtype, to gain a better insight into risk factors for each. For the rare subtypes, such as triple negatives, this will require collaborative efforts to provide sufficient numbers of cases. In terms of public health implications, despite relatively low incidence rates, African women have mortality rates from breast cancer that are as high as in high incidence countries [1]. If more aggressive breast tumors predominated, the potential to improve survival rates would be curtailed using current therapies. However, the present synthesis suggests that this is not the case, and that two-thirds of women with breast cancer have a less aggressive disease form for which targeted endocrine treatments have been shown to produce good survival rates. Tamoxifen [4], in particular, may provide an effective therapeutic option because of its low cost and ease of administration. Improving prognosis for such cancers will also hinge on the ability to diagnose and commence treatment at earlier stages of the disease, which is needed across many African countries as several hospitals have over 70% of breast cancer patients being diagnosed at stage III/IV. With a majority of ER+ tumors, this less-aggressive disease is also consistent with relatively long (6–18 months) symptomatic periods reported by women prior to diagnosis. This is a time-window during which efforts to encourage earlier presentation and faster referral through health systems to treatment centres can be focussed.

## Supporting Information

Figure S1 **Proportion of PR+ disease by study design, North and sub-Saharan Africa.** IBC, inflammatory breast cancer; LABC, non-IBC locally advanced breast cancer; N-IBC, non-inflammatory breast cancer. *These studies did not provide separate ER and PR estimates; only an HR estimate for tumors that were ER+ or PR+ [33] or ER+ and/or PR+ ([29]; [43]; [52]; [63]; [64]; [71]).
(PDF)

Figure S2 **Proportion of HER2+ disease by study design, North and sub-Saharan Africa.** IBC, inflammatory breast cancer; LABC, non-IBC locally advanced breast cancer; N-IBC, non-inflammatory breast cancer.
(PDF)

Figure S3 **Proportion of PR+ disease by year of diagnosis, North and sub-Saharan Africa.** IBC, inflammatory breast cancer; LABC, non-IBC locally advanced breast cancer; N-IBC, non-inflammatory breast cancer. *These studies did not provide separate ER and PR estimates; only an HR estimate for tumors that were ER+ or PR+ [33] or ER+ and/or PR+ ([29]; [43]; [52]; [63]; [64]; [71]).
(PDF)

Figure S4 **Proportion of HER2+ disease by year of diagnosis, North and sub-Saharan Africa.** IBC, inflammatory breast cancer; LABC, non-IBC locally advanced breast cancer; N-IBC, non-inflammatory breast cancer.
(PDF)

Figure S5 **Proportion of ER+ disease by tumor grade for the 12 studies that provided grade-specific estimates.** *Grade 1 tumors (*n* = 17) were excluded; **grade 1 tumors (*n* = 5) were excluded.
(PDF)

Figure S6 **Proportion of PR+ disease by tumor grade, North and sub-Saharan Africa.** IBC, inflammatory breast cancer; LABC, non-IBC locally advanced breast cancer; N-IBC, non-inflammatory breast cancer. *These studies did not provide separate ER and PR estimates; only an HR estimate for tumors that were ER+ or PR+ [33] or ER+ and/or PR+ ([43]; [52]; 2013 [63]; [64]; [71]).
(PDF)

Figure S7 **Proportion of HER2+ disease by tumor grade, North and sub-Saharan Africa.** IBC, inflammatory breast cancer; LABC, non-IBC locally advanced breast cancer; N-IBC, non-inflammatory breast cancer.
(PDF)

Figure S8 **Proportion of PR+ disease by timing of receptor testing, North and sub-Saharan Africa.** IBC, inflammatory breast cancer; LABC, non-IBC locally advanced breast cancer. *These studies did not provide separate ER and PR estimates; only an HR estimate for tumors that were ER+ or PR+ [33] or ER+ and/or PR+ ([29]; [43]; [52]; 2013 [63]; [64]; [71]).
(PDF)

Figure S9 **Proportion of HER2+ disease by timing of receptor testing, North and sub-Saharan Africa.** IBC, inflammatory breast cancer; LABC, non-IBC locally advanced breast cancer.
(PDF)

Figure S10 **Proportion of PR+ disease by sub-region within North and sub-Saharan Africa.** IBC, inflammatory breast cancer; LABC, non-IBC locally advanced breast cancer. North-Western Africa: Morocco, Algeria, and Tunisia; North-Eastern Africa: Egypt, Sudan, and Libya; Eastern Africa: Kenya, Uganda, Tanzania, and Madagascar; Western Africa: Ghana, Mali, Nigeria, and Senegal); Sothern Africa: South Africa. *These studies provided only a combined HR estimate for tumors that were either ER+ or PR+ [33] or ER+ and/or PR+ ([29]; [43]; [52]; 2013 [63]; [64]; [71]). **Lower limit of 95% confidence interval for I^2^ statistic truncated at 0.
(PDF)

Figure S11 **Proportion of HER2+ disease by sub-region within North and sub-Saharan Africa.** IBC, inflammatory breast cancer; LABC, non-IBC locally advanced breast cancer. North-Western Africa: Morocco, Algeria, and Tunisia; North-Eastern Africa: Egypt, Sudan, and Libya; Eastern Africa: Kenya, Uganda, Tanzania, and Madagascar; Western Africa: Ghana, Mali, Nigeria, and Senegal; Sothern Africa: South Africa. *Lower limit of 95% confidence interval for I^2^ statistic truncated at 0.
(PDF)

Figure S12 **Funnel plots (with pseudo 95% confidence limits) for published ER+, PR+, and HER2+ studies, North and sub-Saharan Africa.**
(PDF)

Alternative Language Abstract S1 **French and Portuguese translations of the title and abstract by VM and IdSS, respectively.**
(DOCX)

Text S1 **PRISMA checklist of items to include when reporting a systematic review.**
(DOC)

Text S2 **Protocol of the systematic review.**
(DOCX)

Text S3 **Terms used in the literature search.**
(DOCX)

## Abbreviations

CISH: chromogenic in situ hybridization
ER: estrogen receptor
FISH: fluorescent in situ hybridization
FFPE: formalin-fixed paraffin-embedded
HER2: human epidermal growth factor-2
HR: hormone receptor
IHC: immunohistochemistry
PR: progesterone receptor
prop: proportion of receptor-positive tumors
SISH: silver in situ hybridization
