# Supplementary figures and images for: Receptor-Defined Subtypes of Breast Cancer in Indigenous Populations in Africa: A Systematic Review and Meta-Analysis
Source: PLoS Med. 2014 Sep 9;11(9):e1001720. doi: 10.1371/journal.pmed.1001720 (PMC4159229; doi:10.1371/journal.pmed.1001720)

## North Africa

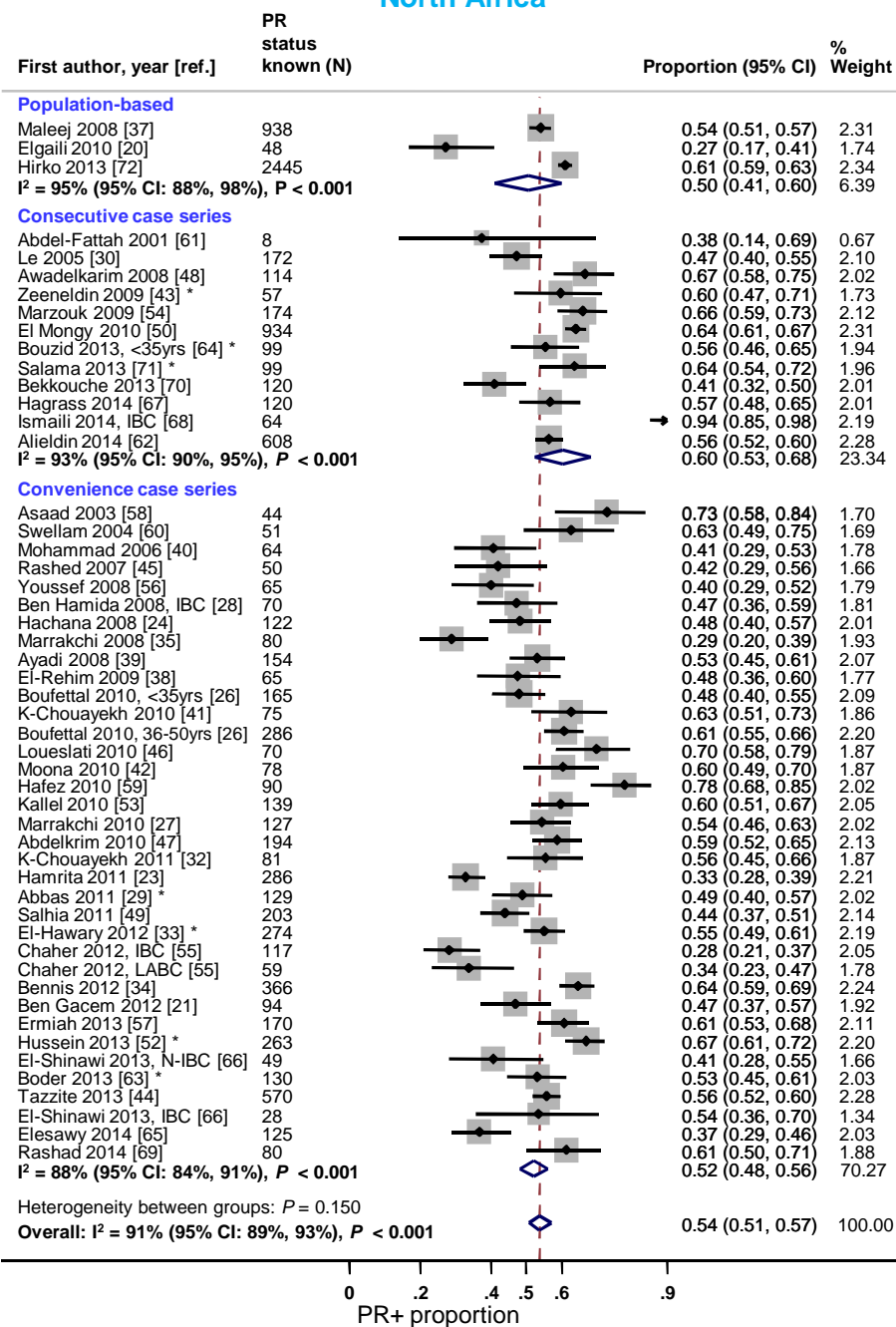

## Sub-Saharan Africa

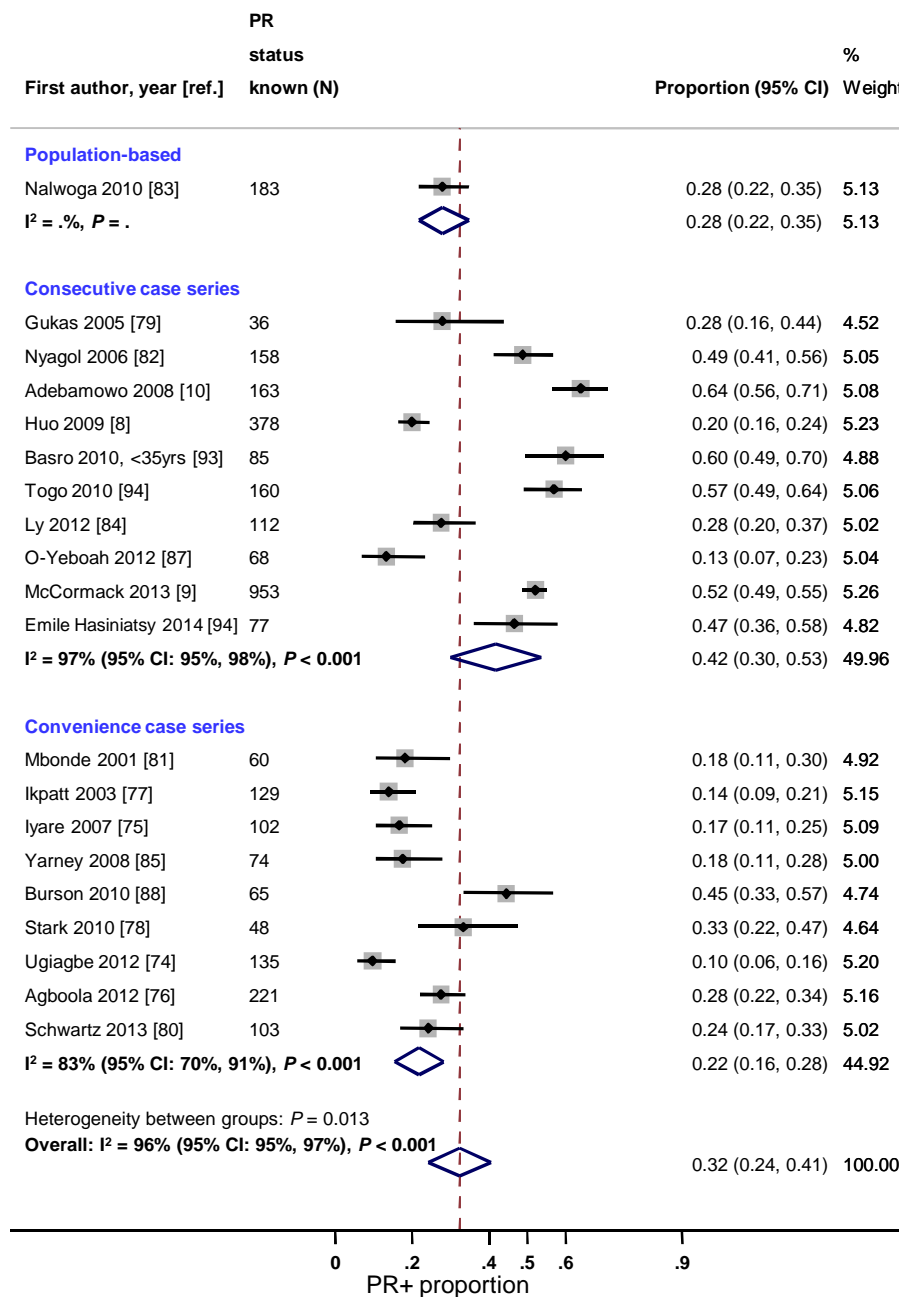

Supplement: Figure S1 — Proportion of PR+ disease by study design, North and sub-Saharan Africa. IBC, inflammatory breast cancer; LABC, non-IBC locally advanced breast cancer; N-IBC, non-inflammatory breast cancer. *These studies did not provide separate ER and PR estimates; only an HR estimate for tumors that were ER+ or PR+ [33] or ER+ and/or PR+ ([29]; [43]; [52]; [63]; [64]; [71]). (PDF) [file pmed.1001720.s001.pdf]

## North Africa

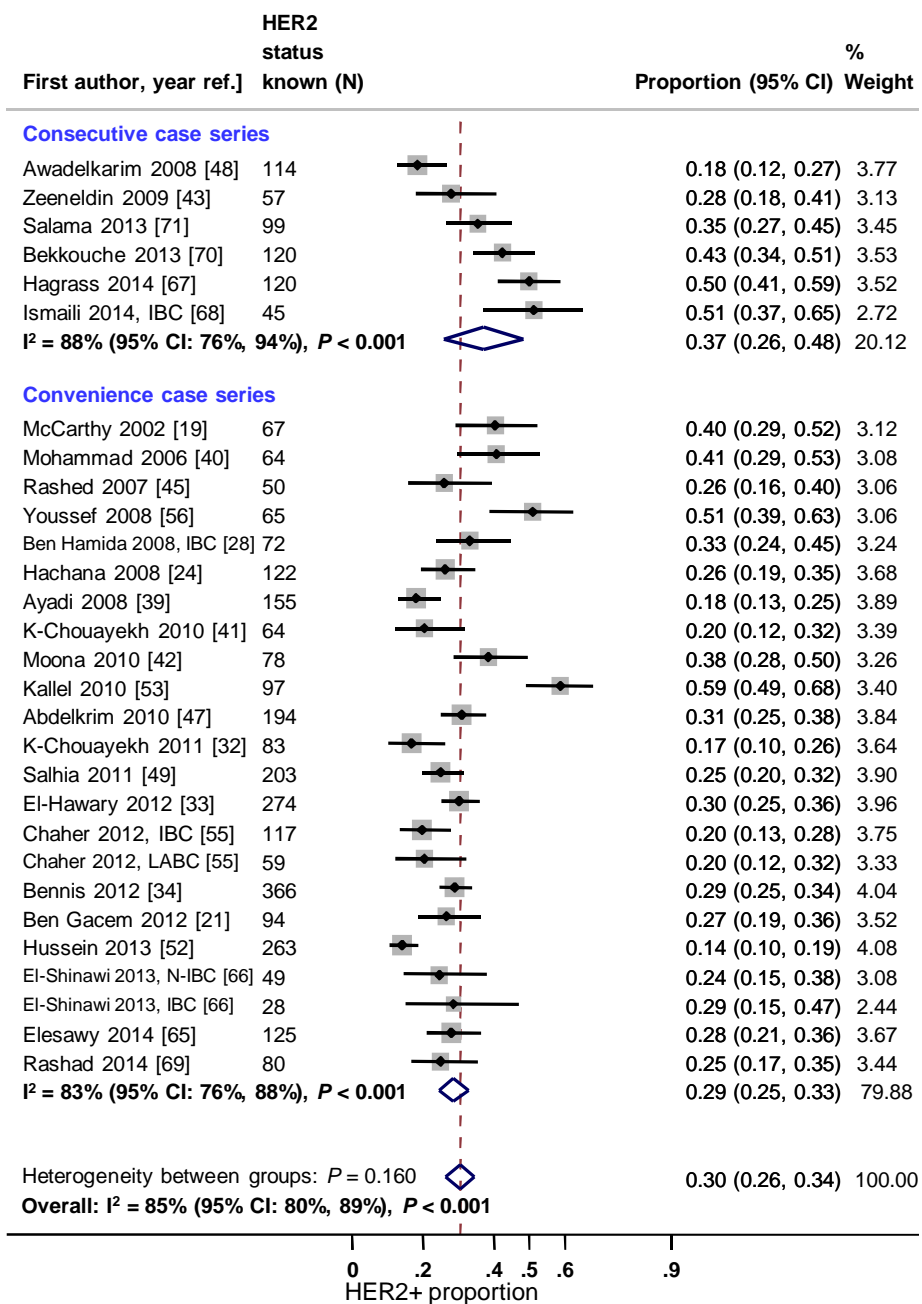

## Sub-Saharan Africa

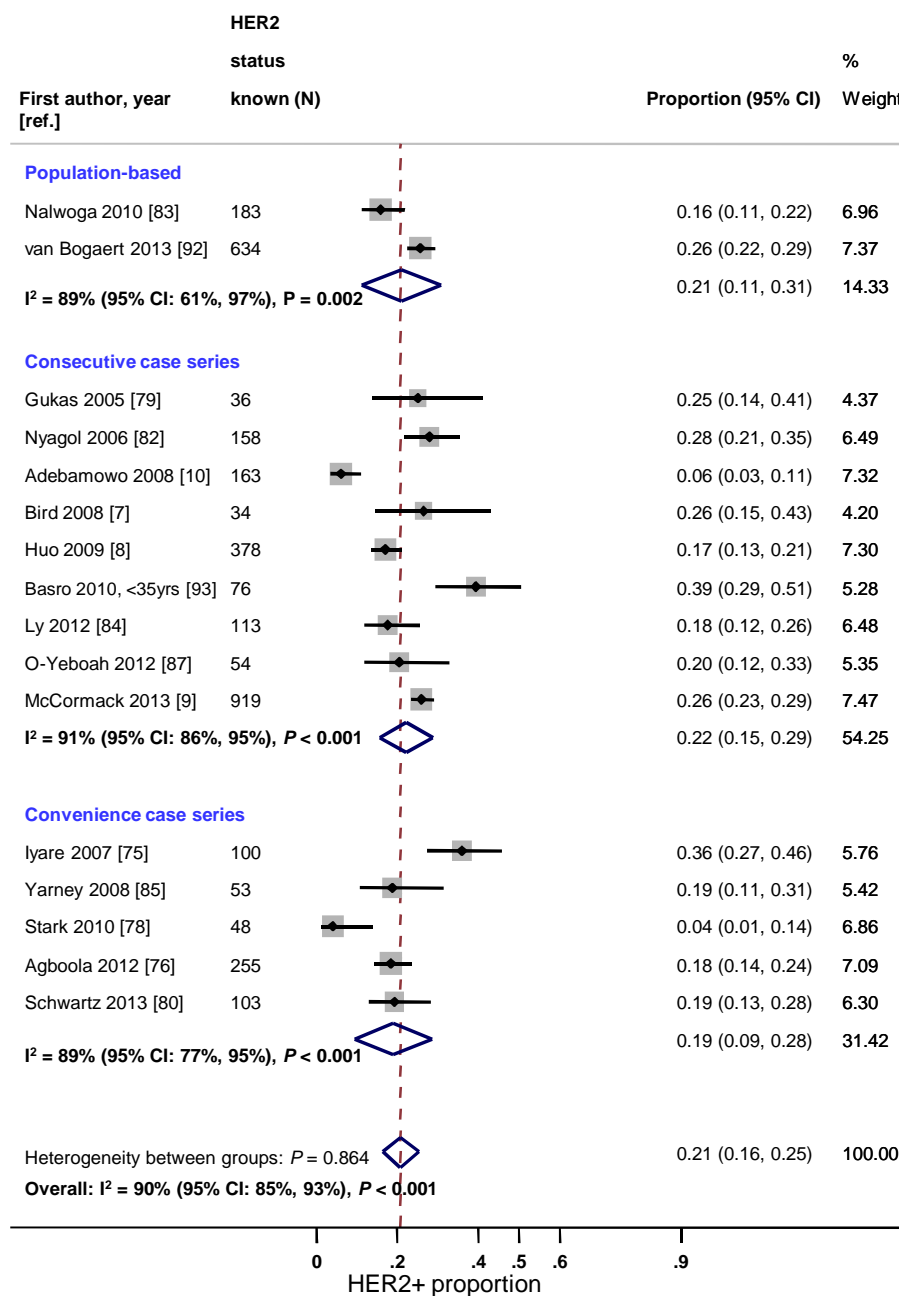

Supplement: Figure S2 — Proportion of HER2+ disease by study design, North and sub-Saharan Africa. IBC, inflammatory breast cancer; LABC, non-IBC locally advanced breast cancer; N-IBC, non-inflammatory breast cancer. (PDF) [file pmed.1001720.s002.pdf]

## North Africa

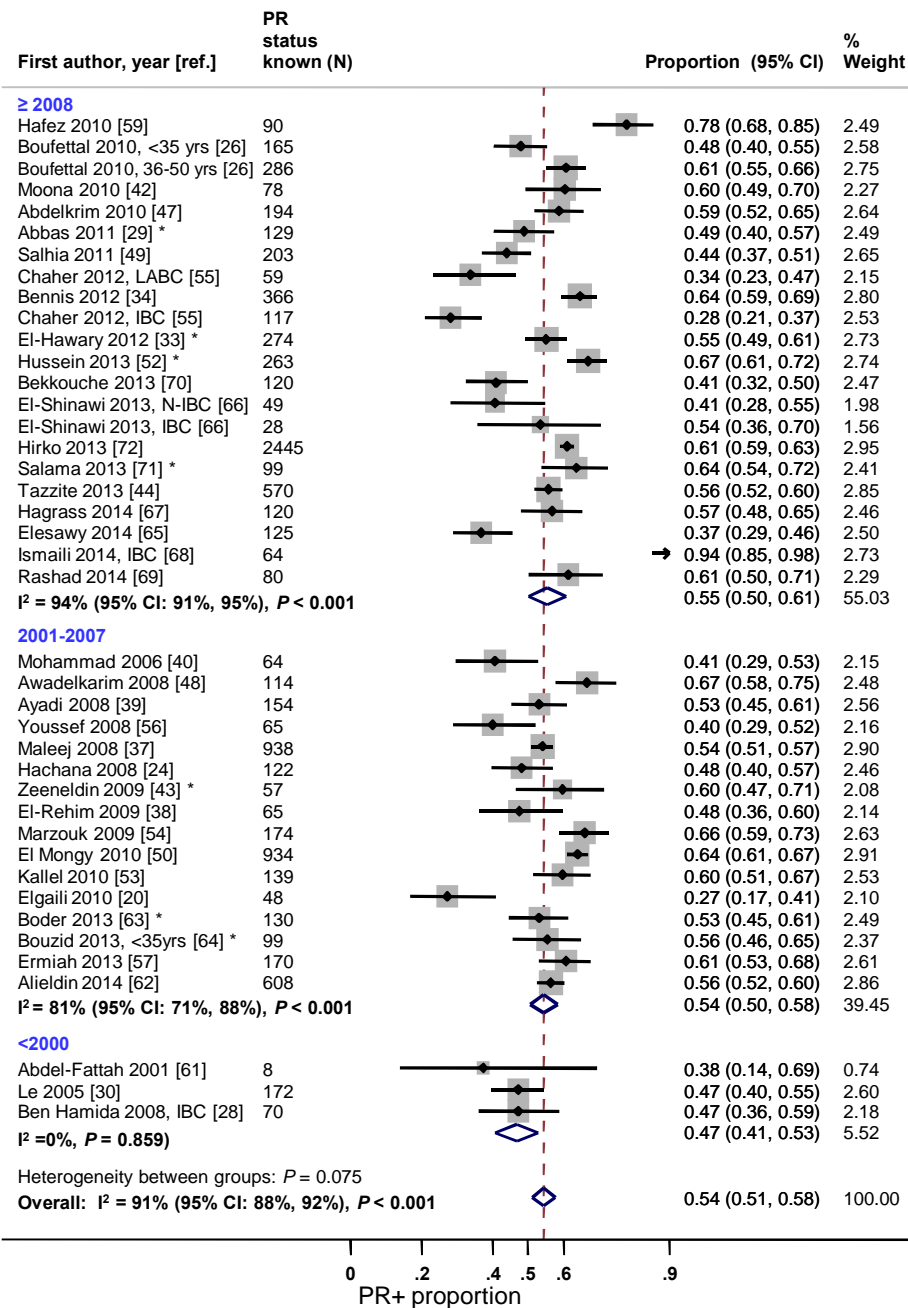

## Sub-Saharan Africa

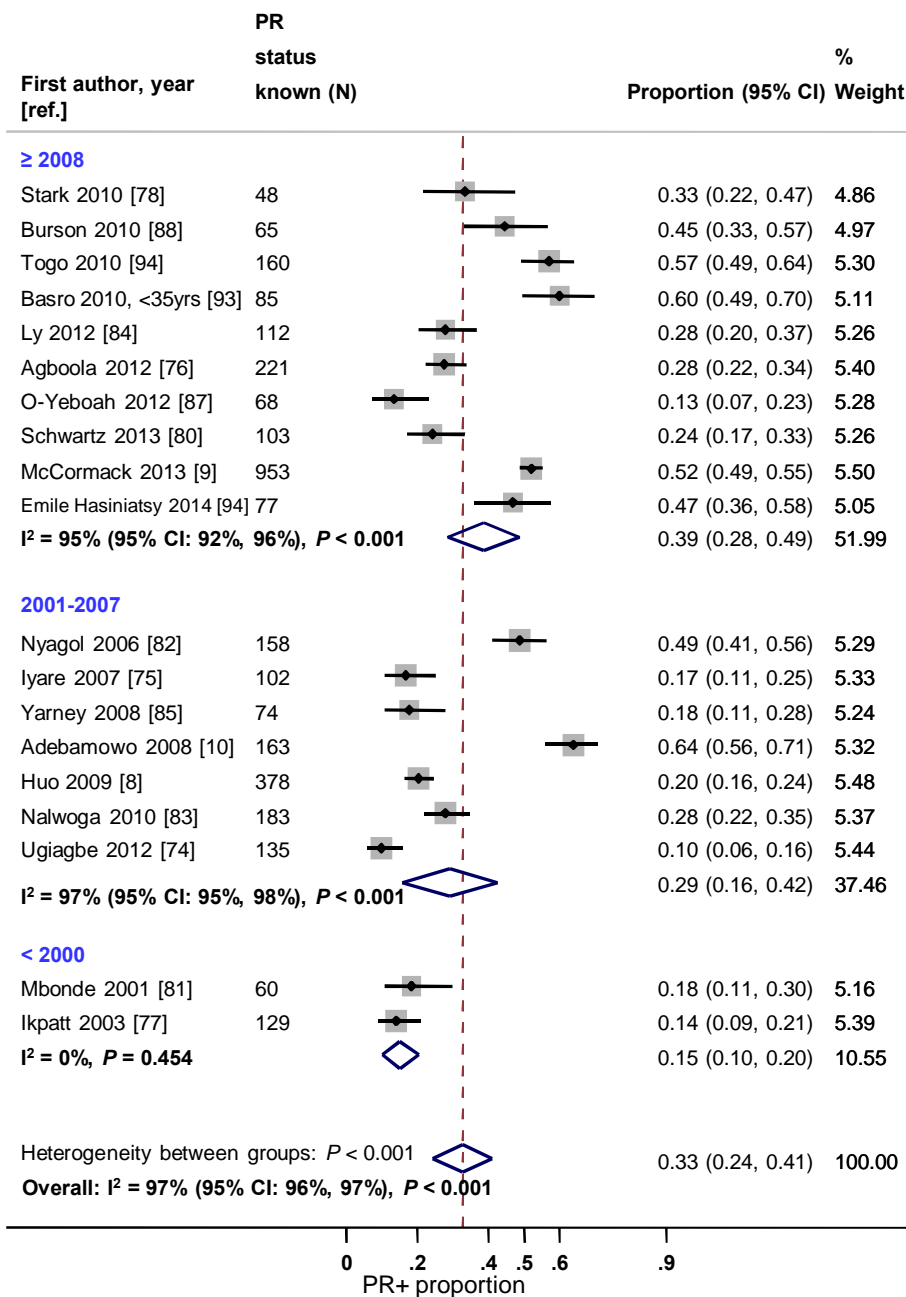

Supplement: Figure S3 — Proportion of PR+ disease by year of diagnosis, North and sub-Saharan Africa. IBC, inflammatory breast cancer; LABC, non-IBC locally advanced breast cancer; N-IBC, non-inflammatory breast cancer. *These studies did not provide separate ER and PR estimates; only an HR estimate for tumors that were ER+ or PR+ [33] or ER+ and/or PR+ ([29]; [43]; [52]; [63]; [64]; [71]). (PDF) [file pmed.1001720.s003.pdf]

## North Africa

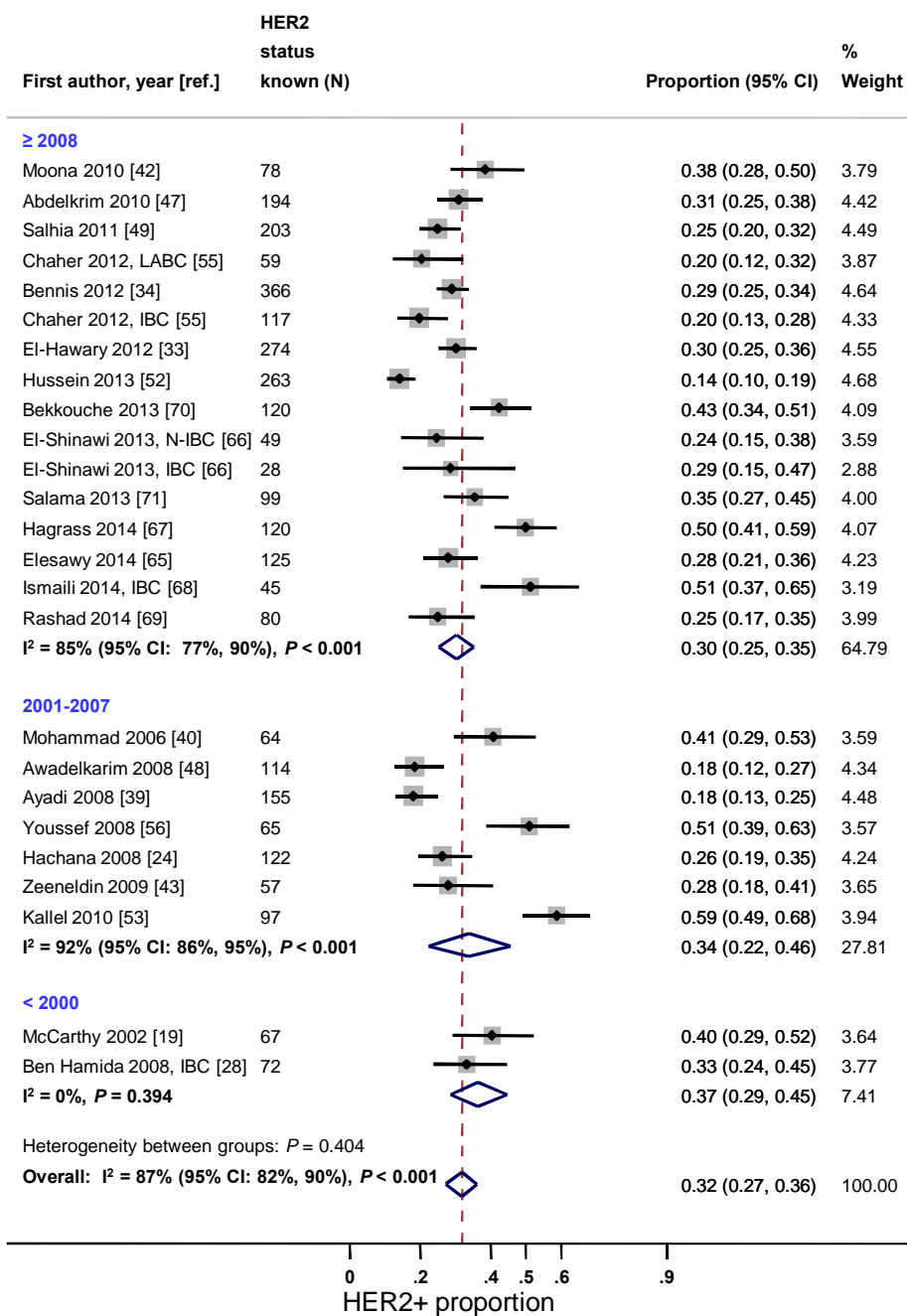

## Sub-Saharan Africa

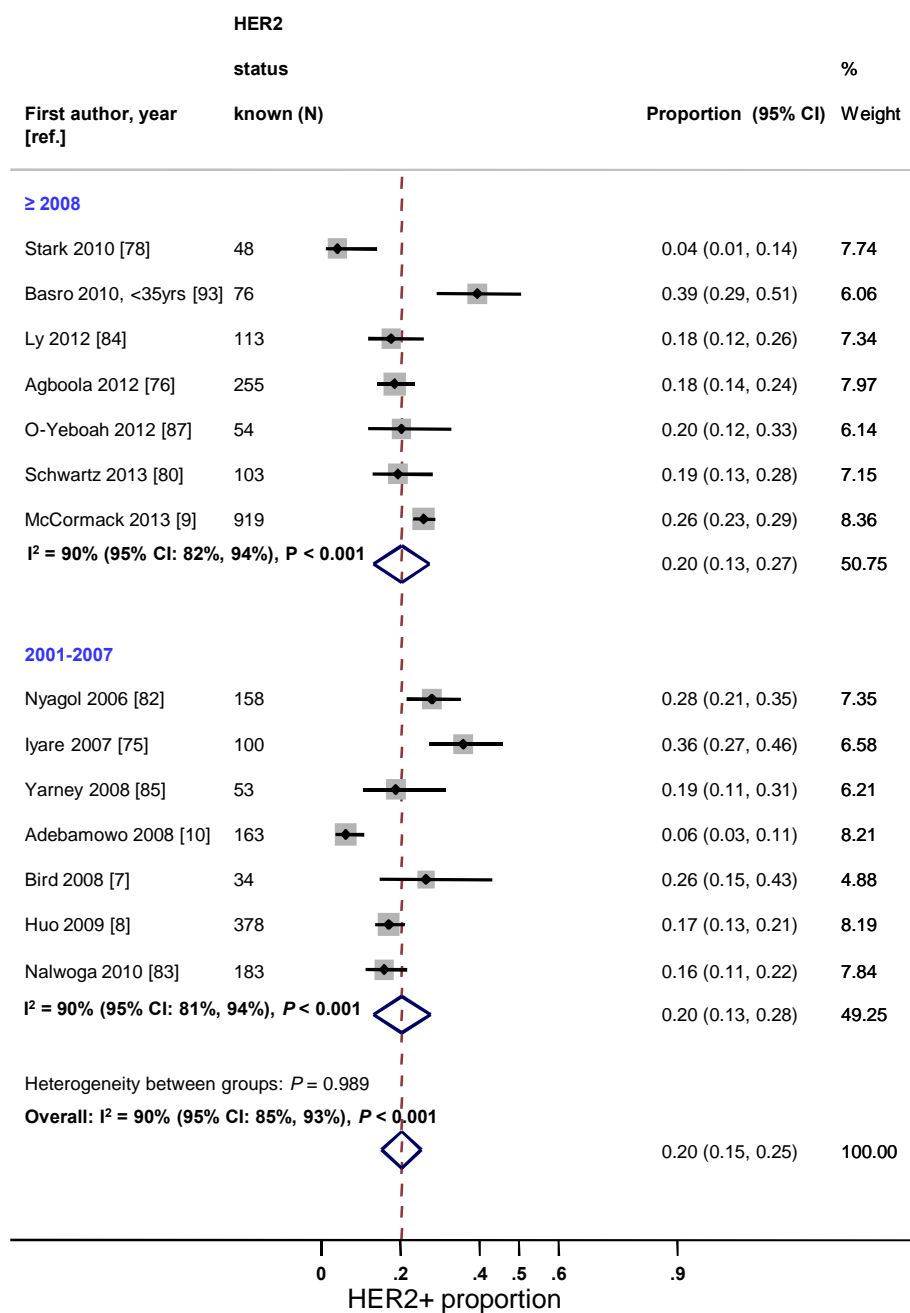

Supplement: Figure S4 — Proportion of HER2+ disease by year of diagnosis, North and sub-Saharan Africa. IBC, inflammatory breast cancer; LABC, non-IBC locally advanced breast cancer; N-IBC, non-inflammatory breast cancer. (PDF) [file pmed.1001720.s004.pdf]

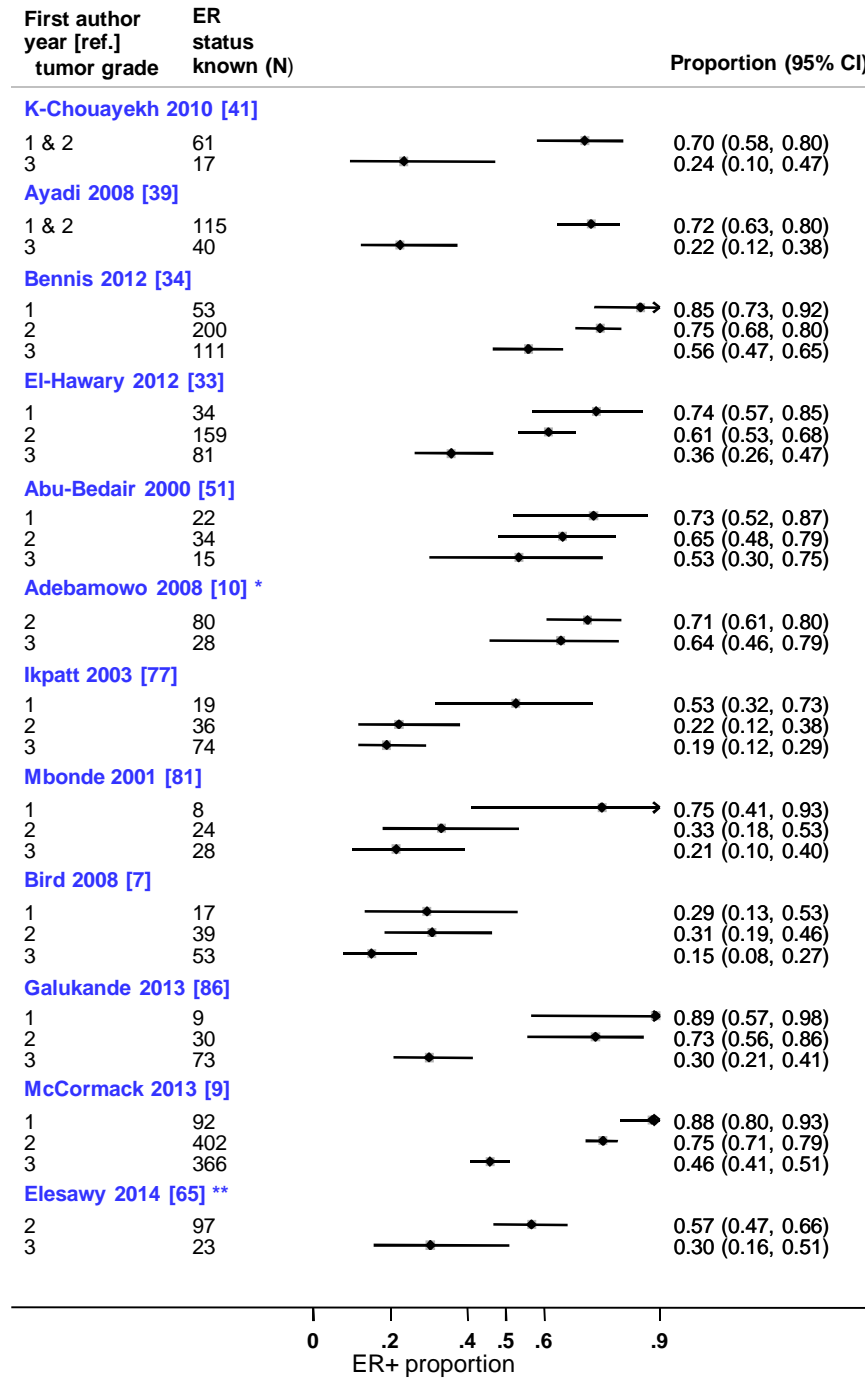

Supplement: Figure S5 — Proportion of ER+ disease by tumor grade for the 12 studies that provided grade-specific estimates. *Grade 1 tumors (n = 17) were excluded; **grade 1 tumors (n = 5) were excluded. (PDF) [file pmed.1001720.s005.pdf]

## North Africa

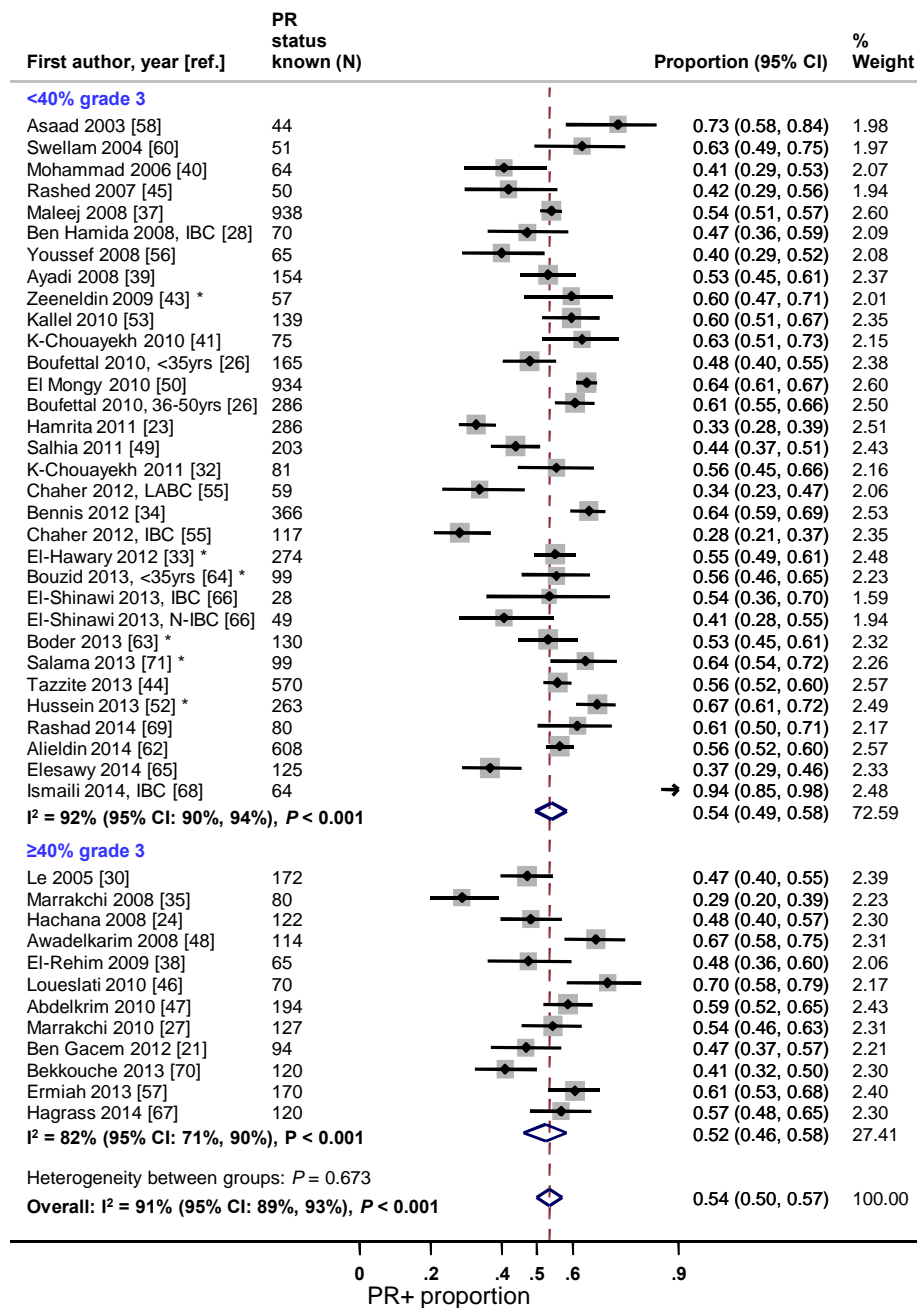

## Sub-Saharan Africa

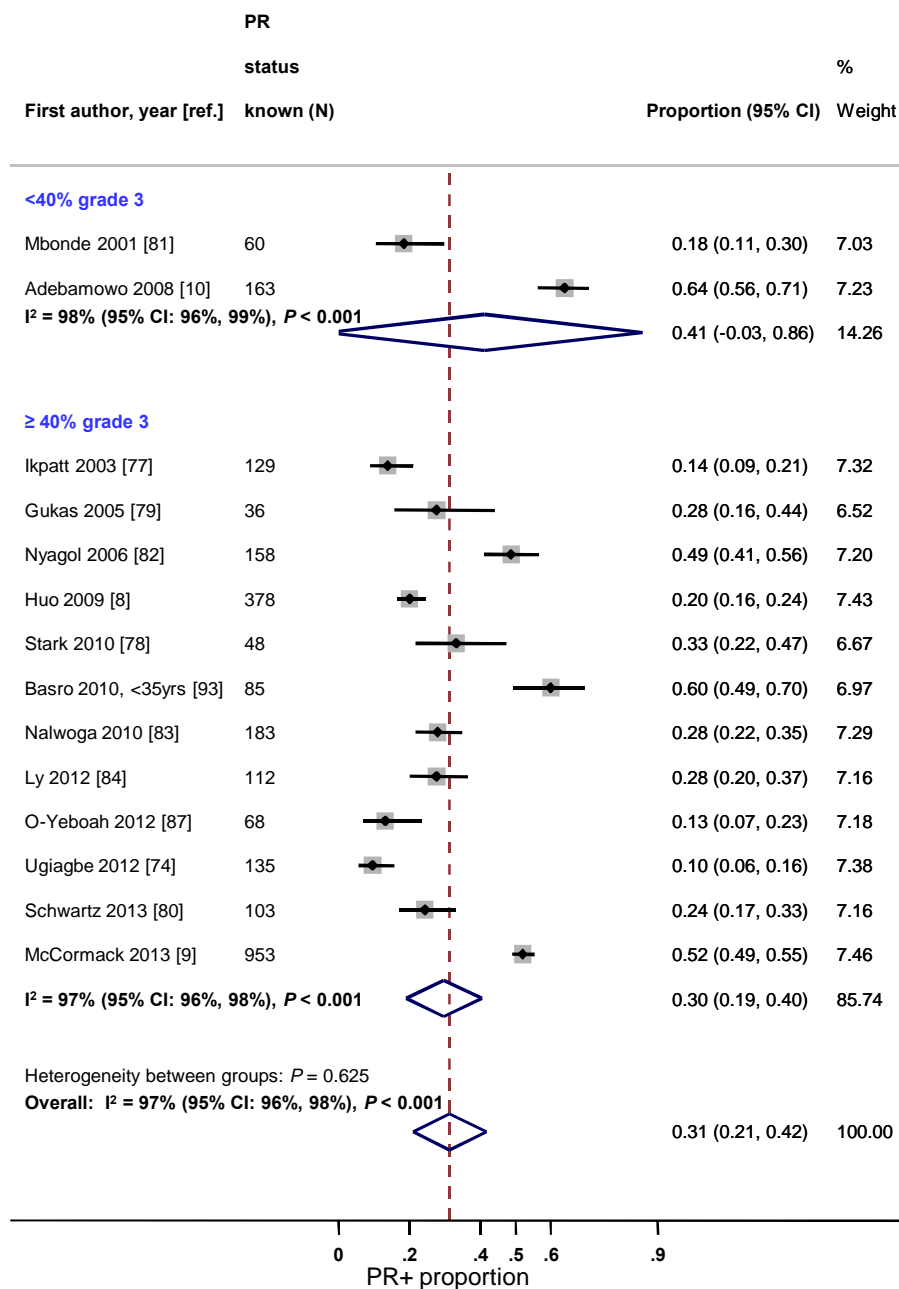

Supplement: Figure S6 — Proportion of PR+ disease by tumor grade, North and sub-Saharan Africa. IBC, inflammatory breast cancer; LABC, non-IBC locally advanced breast cancer; N-IBC, non-inflammatory breast cancer. *These studies did not provide separate ER and PR estimates; only an HR estimate for tumors that were ER+ or PR+ [33] or ER+ and/or PR+ ([43]; [52]; 2013 [63]; [64]; [71]). (PDF) [file pmed.1001720.s006.pdf]

## North Africa

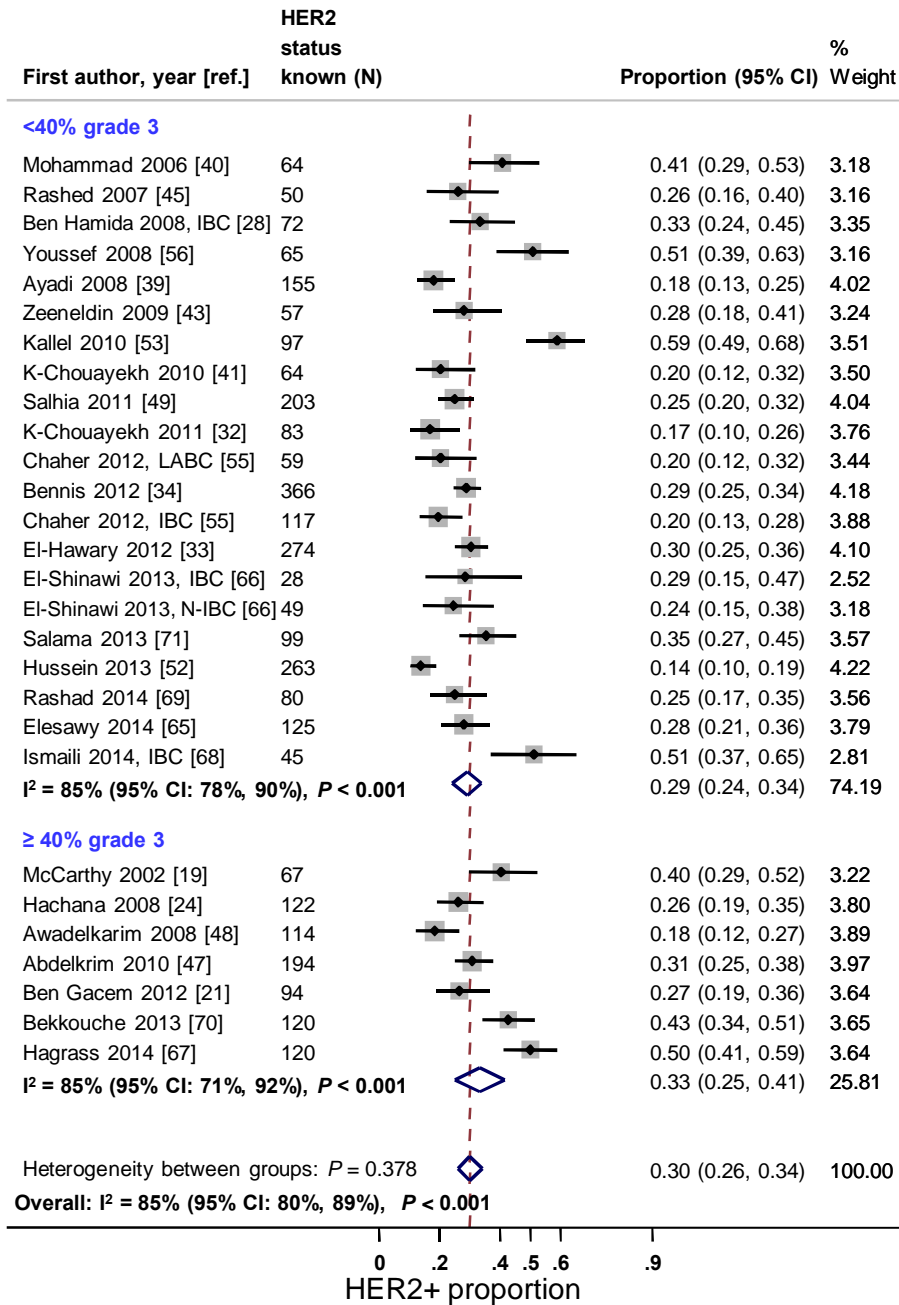

## Sub-Saharan Africa

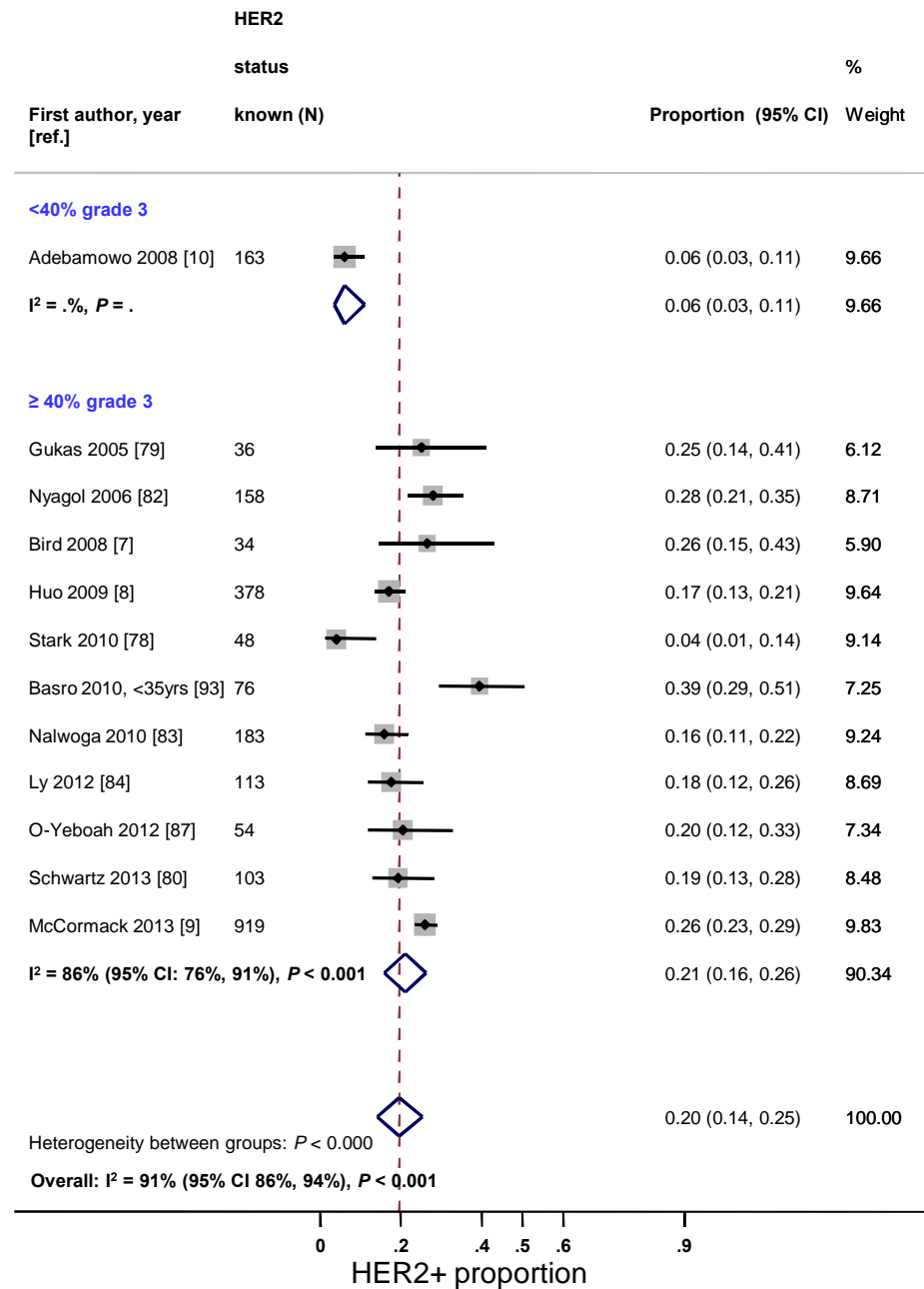

Supplement: Figure S7 — Proportion of HER2+ disease by tumor grade, North and sub-Saharan Africa. IBC, inflammatory breast cancer; LABC, non-IBC locally advanced breast cancer; N-IBC, non-inflammatory breast cancer. (PDF) [file pmed.1001720.s007.pdf]

## North Africa

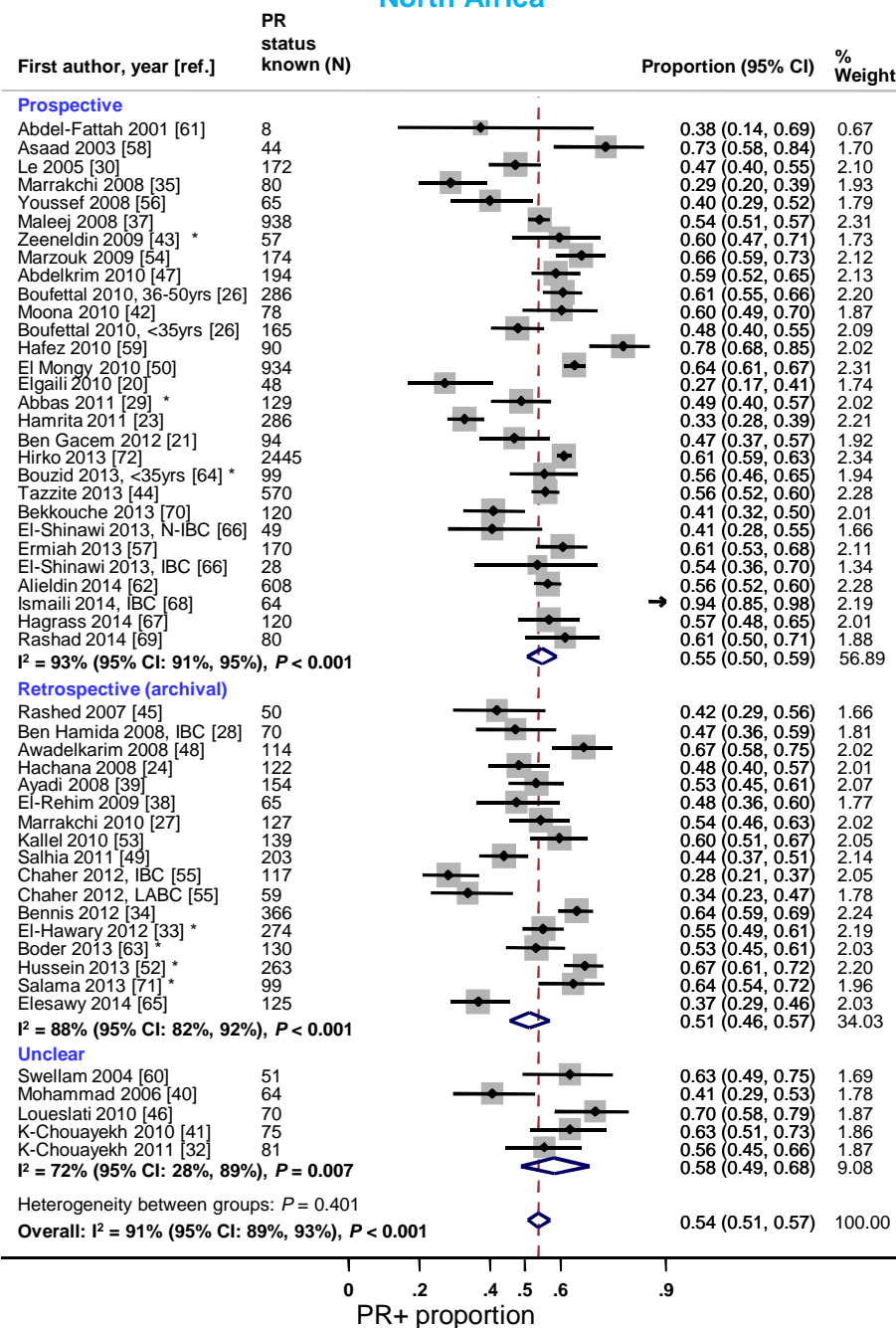

## Sub-Saharan Africa

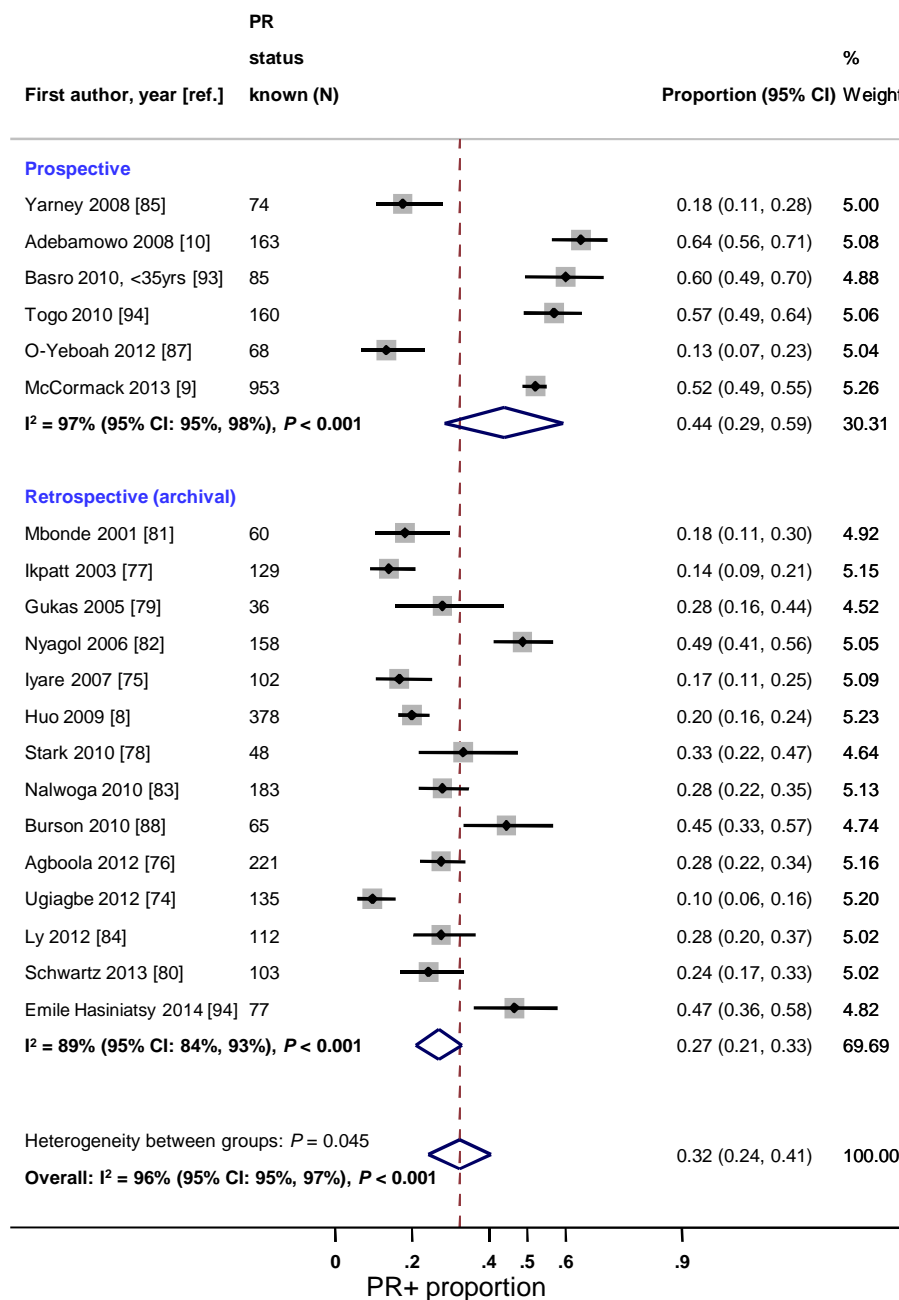

Supplement: Figure S8 — Proportion of PR+ disease by timing of receptor testing, North and sub-Saharan Africa. IBC, inflammatory breast cancer; LABC, non-IBC locally advanced breast cancer. *These studies did not provide separate ER and PR estimates; only an HR estimate for tumors that were ER+ or PR+ [33] or ER+ and/or PR+ ([29]; [43]; [52]; 2013 [63]; [64]; [71]). (PDF) [file pmed.1001720.s008.pdf]

## North Africa

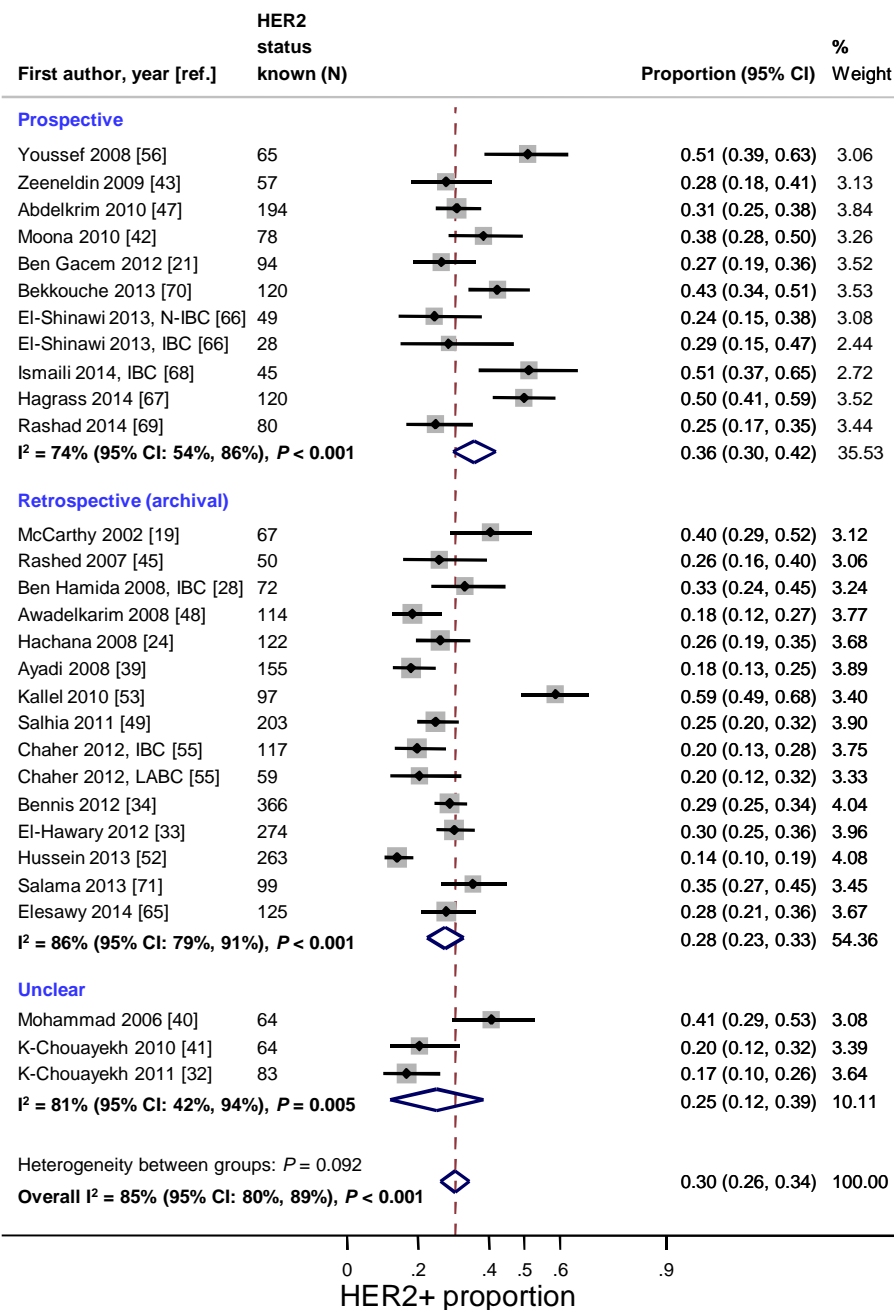

## Sub-Saharan Africa

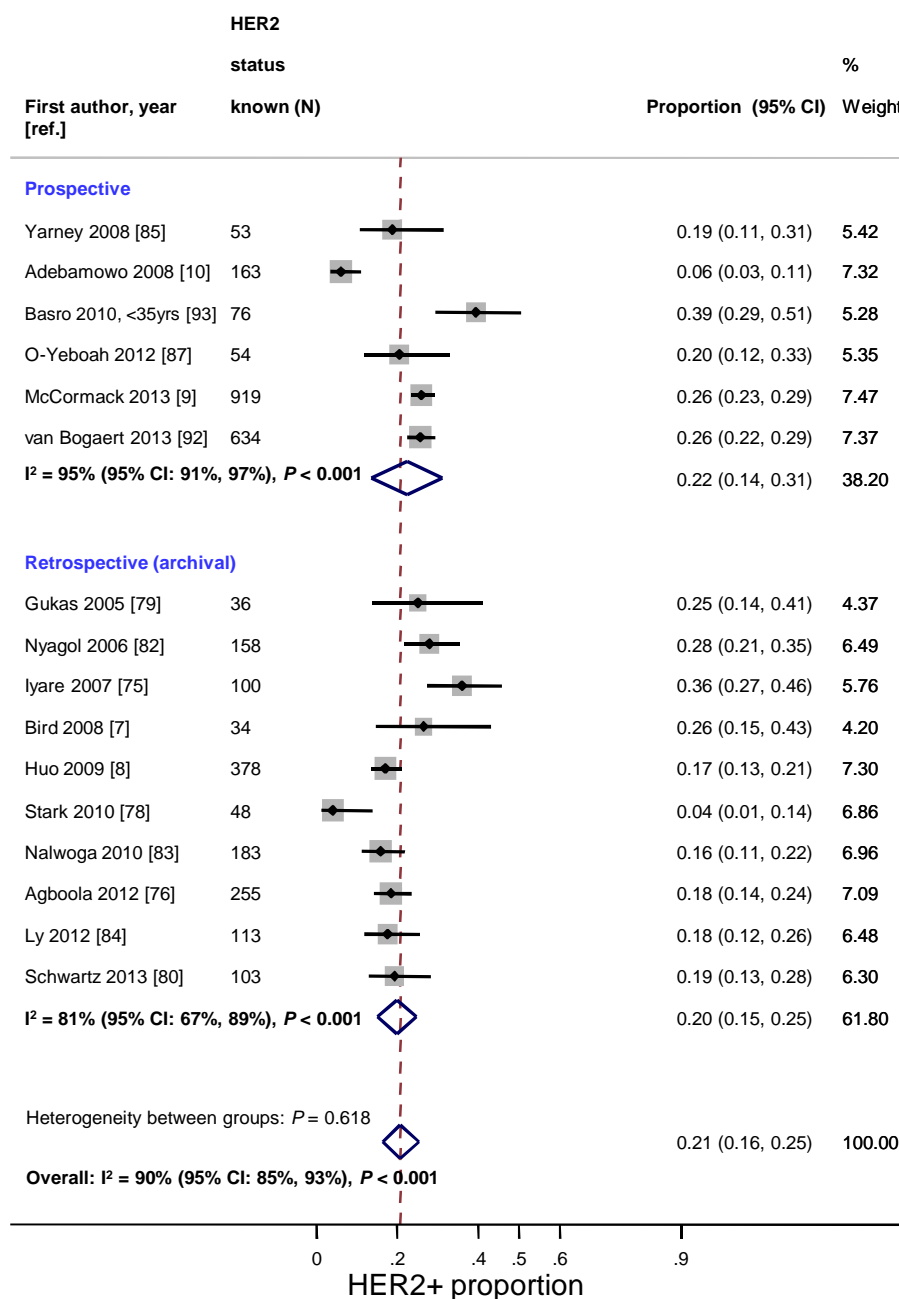

Supplement: Figure S9 — Proportion of HER2+ disease by timing of receptor testing, North and sub-Saharan Africa. IBC, inflammatory breast cancer; LABC, non-IBC locally advanced breast cancer. (PDF) [file pmed.1001720.s009.pdf]

## North Africa

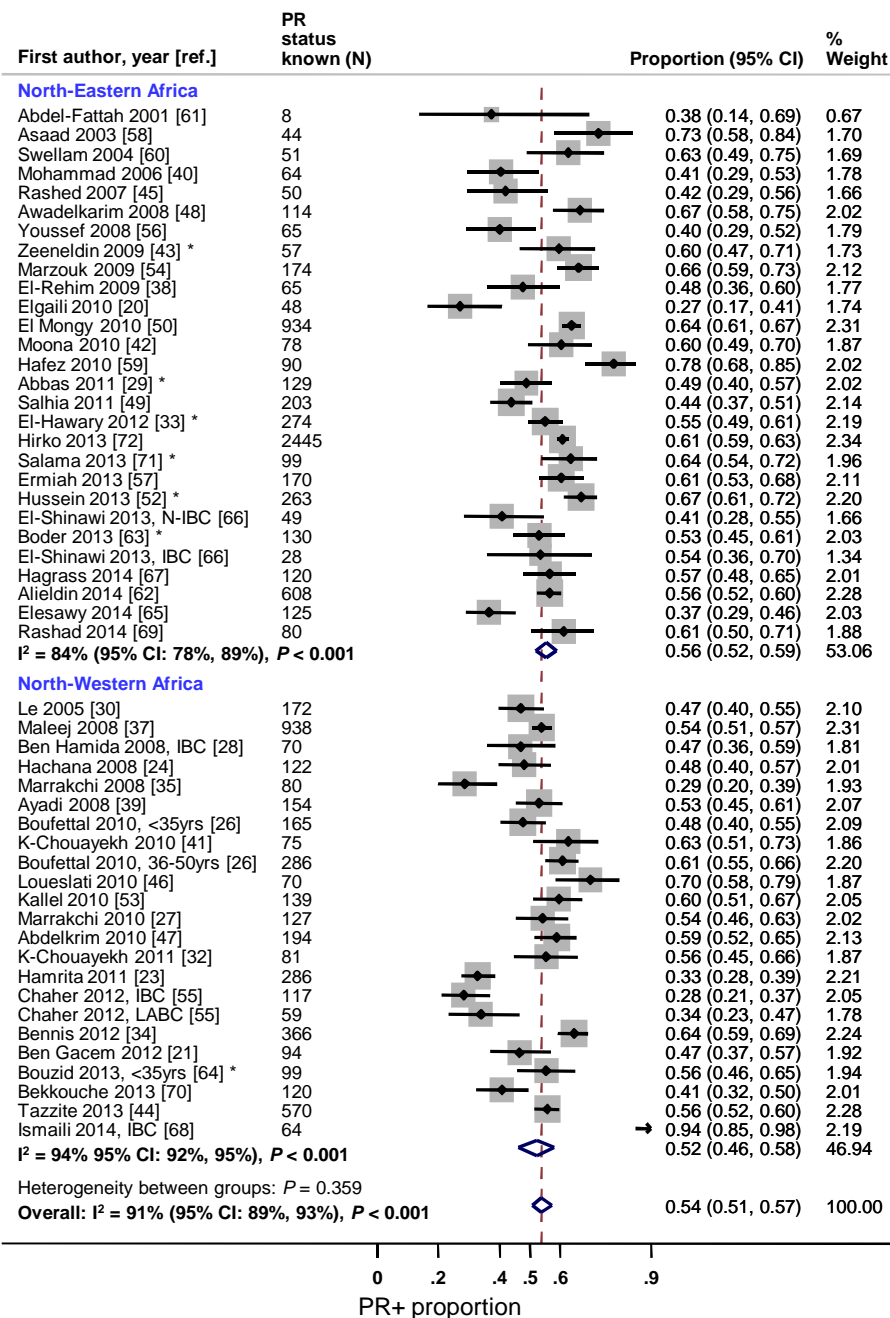

## Sub-Saharan Africa

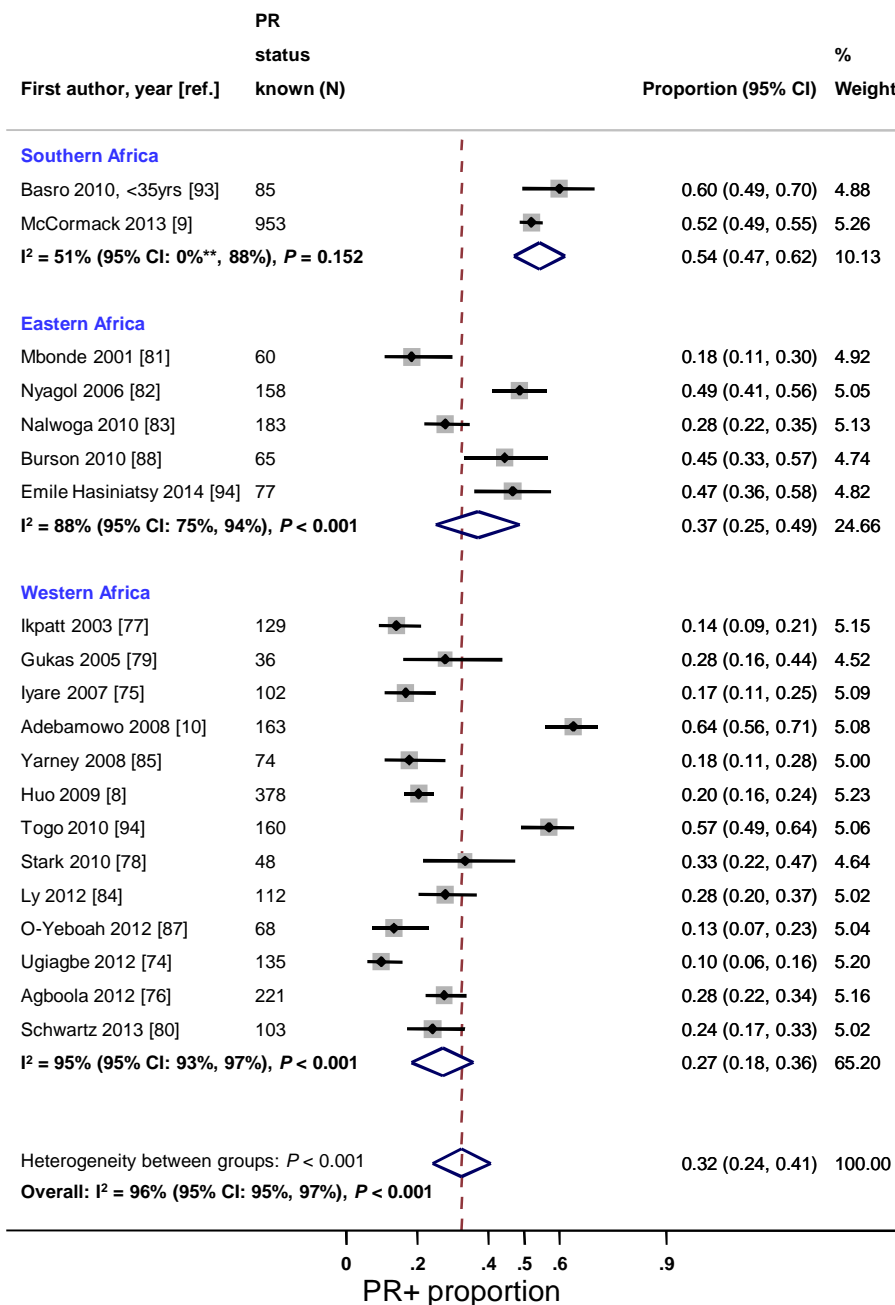

Supplement: Figure S10 — Proportion of PR+ disease by sub-region within North and sub-Saharan Africa. IBC, inflammatory breast cancer; LABC, non-IBC locally advanced breast cancer. North-Western Africa: Morocco, Algeria, and Tunisia; North-Eastern Africa: Egypt, Sudan, and Libya; Eastern Africa: Kenya, Uganda, Tanzania, and Madagascar; Western Africa: Ghana, Mali, Nigeria, and Senegal); Sothern Africa: South Africa. *These studies provided only a combined HR estimate for tumors that were either ER+ or PR+ [33] or ER+ and/or PR+ ([29]; [43]; [52]; 2013 [63]; [64]; [71]). **Lower limit of 95% confidence interval for I2 statistic truncated at 0. (PDF) [file pmed.1001720.s010.pdf]

## North Africa

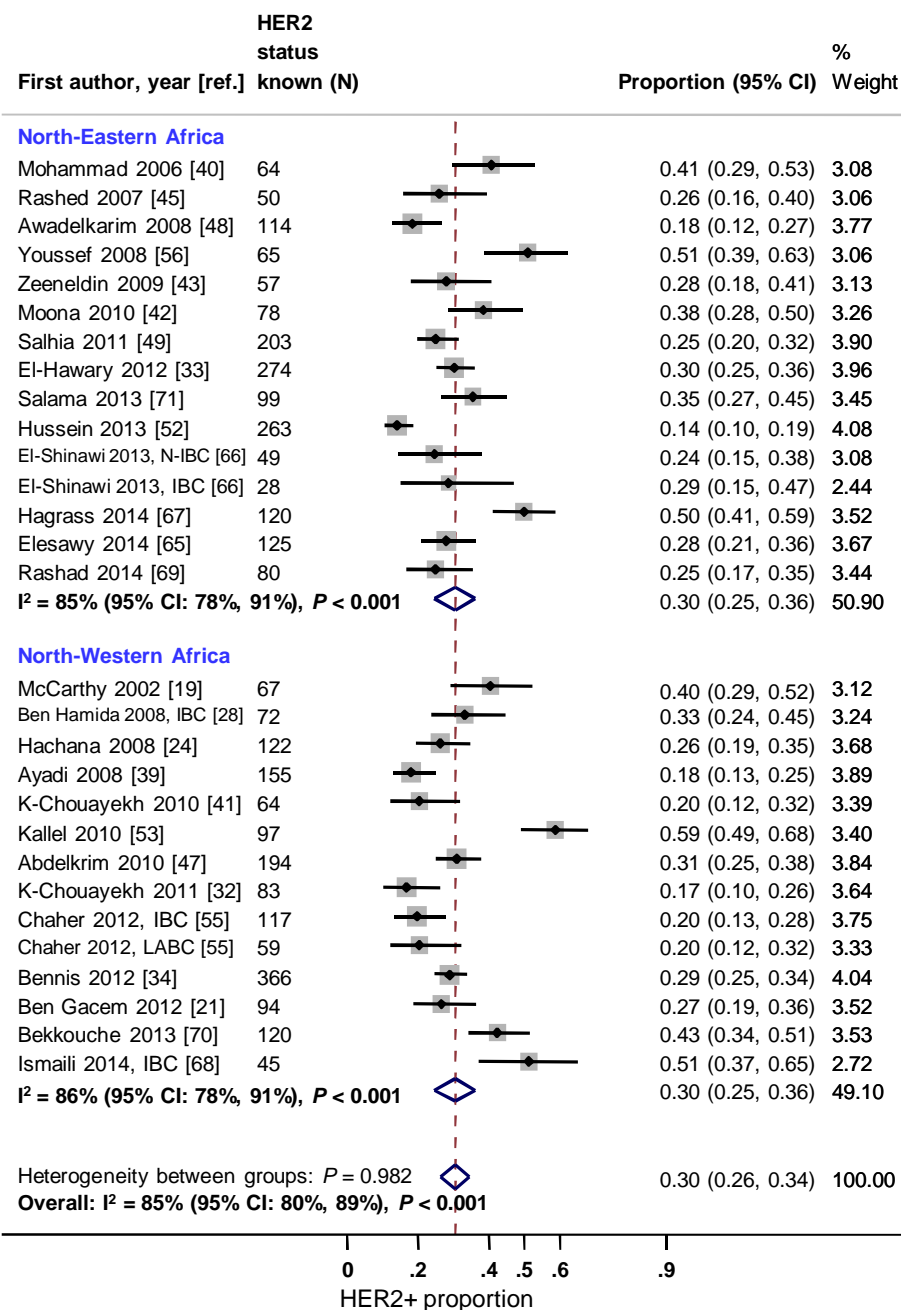

## Sub-Saharan Africa

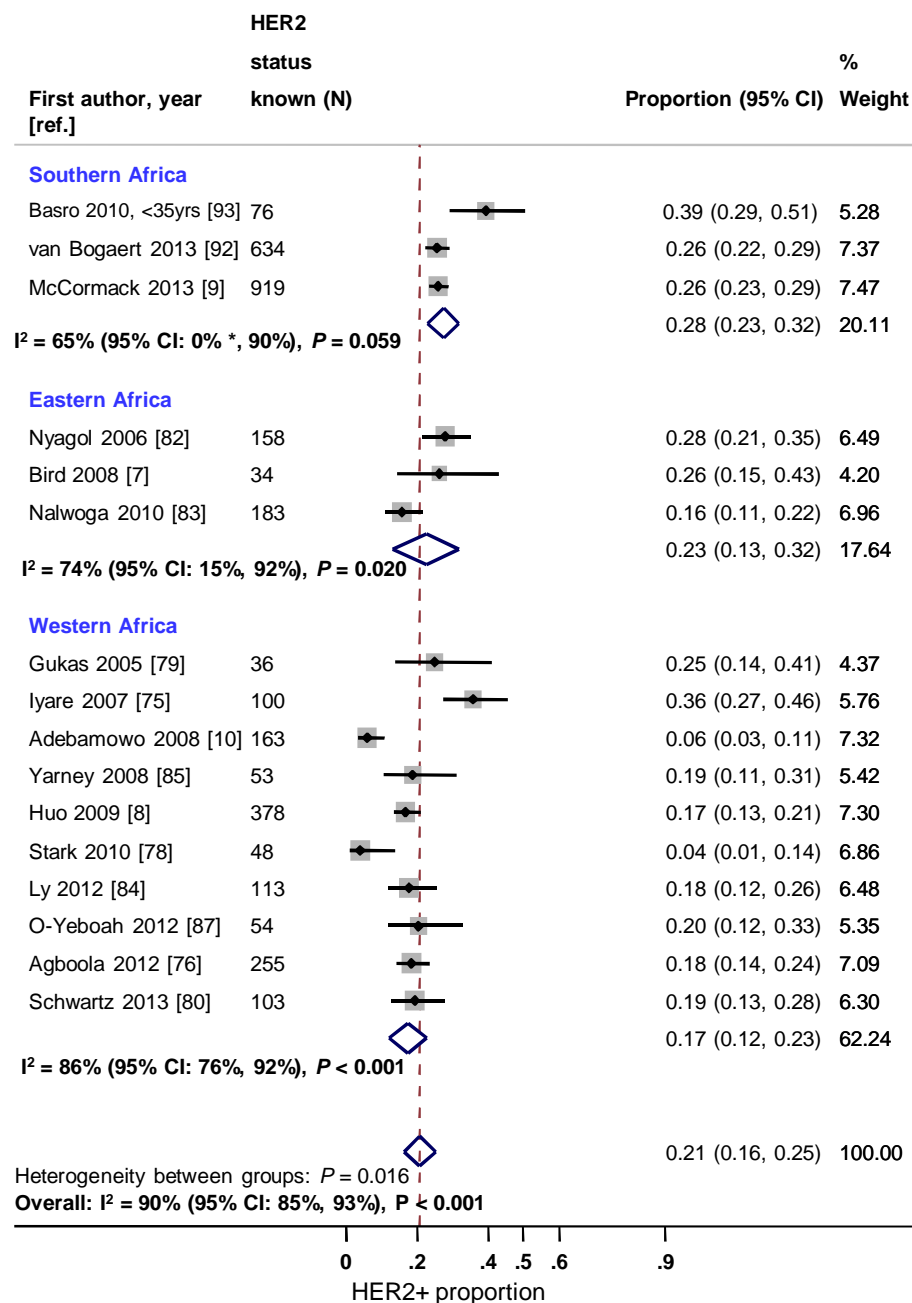

Supplement: Figure S11 — Proportion of HER2+ disease by sub-region within North and sub-Saharan Africa. IBC, inflammatory breast cancer; LABC, non-IBC locally advanced breast cancer. North-Western Africa: Morocco, Algeria, and Tunisia; North-Eastern Africa: Egypt, Sudan, and Libya; Eastern Africa: Kenya, Uganda, Tanzania, and Madagascar; Western Africa: Ghana, Mali, Nigeria, and Senegal; Sothern Africa: South Africa. *Lower limit of 95% confidence interval for I2 statistic truncated at 0. (PDF) [file pmed.1001720.s011.pdf]
