# Supplementary material for: Receptor-Defined Subtypes of Breast Cancer in Indigenous Populations in Africa: A Systematic Review and Meta-Analysis
Source: PLoS Med. 2014 Sep 9;11(9):e1001720. doi: 10.1371/journal.pmed.1001720 (PMC4159229; doi:10.1371/journal.pmed.1001720)
Supplement: Figure S12 — Funnel plots (with pseudo 95% confidence limits) for published ER+, PR+, and HER2+ studies, North and sub-Saharan Africa. (PDF) [file pmed.1001720.s012.pdf]

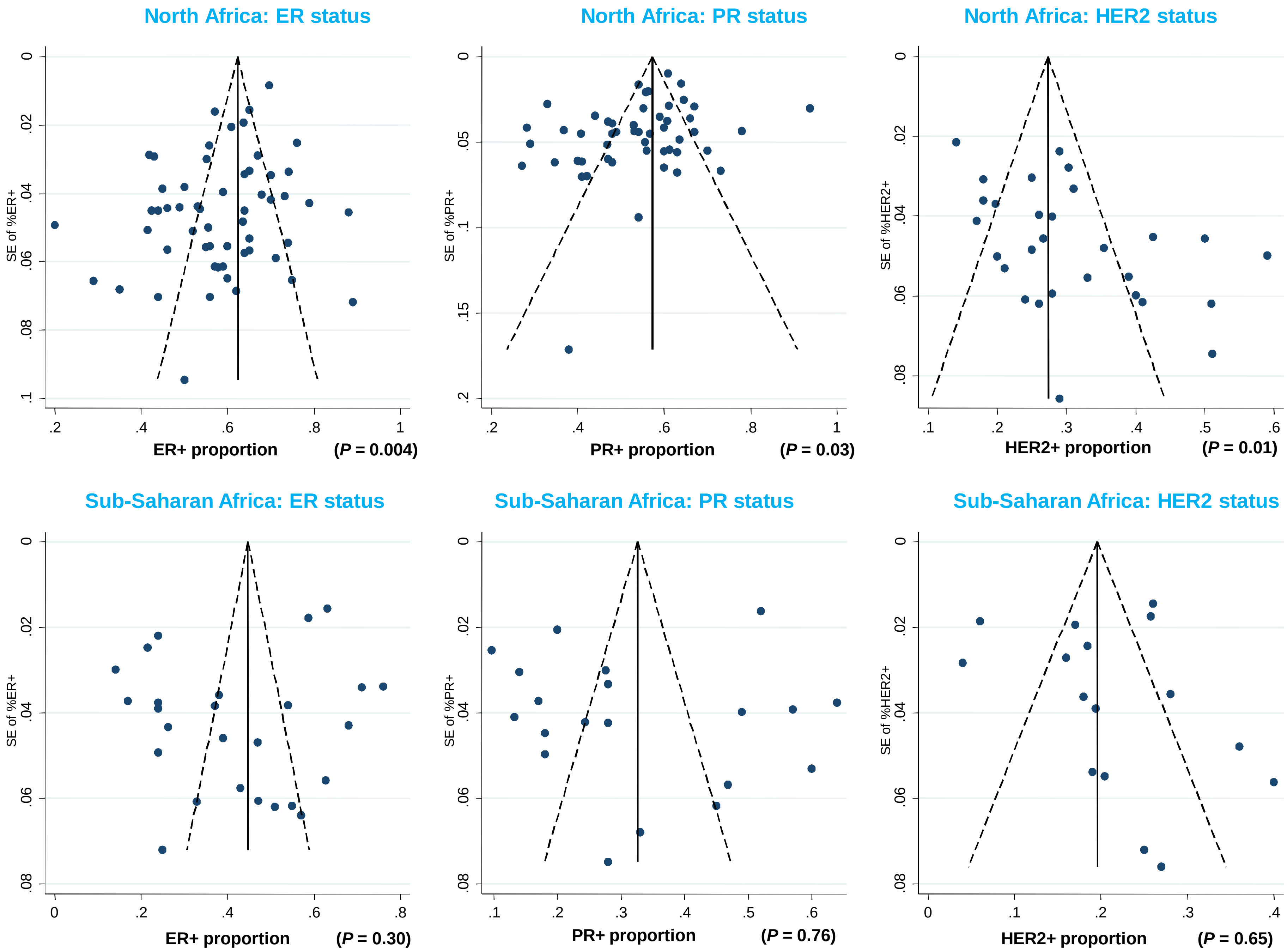

North Africa: ER status
SE of %ER+
ER+ proportion
(P = 0.004)
North Africa: PR status
SE of %PR+
PR+ proportion
(P = 0.03)
North Africa: HER2 status
SE of %HER2+
HER2+ proportion
(P = 0.01)
Sub-Saharan Africa: ER status
SE of %ER+
ER+ proportion
(P = 0.30)
Sub-Saharan Africa: PR status
SE of %PR+
PR+ proportion
(P = 0.76)
Sub-Saharan Africa: HER2 status
SE of %HER2+
HER2+ proportion
(P = 0.65)
