# Supplementary material for: Receptor-Defined Subtypes of Breast Cancer in Indigenous Populations in Africa: A Systematic Review and Meta-Analysis
Source: PLoS Med. 2014 Sep 9;11(9):e1001720. doi: 10.1371/journal.pmed.1001720 (PMC4159229; doi:10.1371/journal.pmed.1001720)
Supplement: Alternative Language Abstract S1 — French and Portuguese translations of the title and abstract by VM and IdSS, respectively. (DOCX) [file pmed.1001720.s013.docx]

**FRENCH TRANSLATION OF THE TITLE AND ABSTRACT**

**Récepteurs et cancer du sein dans les populations d’Afrique: revue systématique et méta-analyse**

**Résumé**

**Contexte:** Le cancer du sein est le cancer féminin le plus fréquent en Afrique. Le type de cancer, défini par l’analyse des récepteurs hormonaux a un rôle déterminant dans le choix des traitements et leurs résultats, mais en Afrique il existe une grande incertitude concernant la fréquence des tumeurs négatives aux récepteurs des oestrogènes de mauvais pronostic. Nous avons analysé systématiquement les publications rapportant la fréquence des types de cancer du sein classés selon le statut des récepteurs dans les populations Africaines.

**Méthodes et Résultats:** Toutes les études publiées entre le 1er janvier 1980 et le 15 avril 2014 ont été recherchées sur Medline, Embase et Global Health. Les proportions de tumeurs positives au récepteur des œstrogènes (ER+), au récepteur de la progestérone (PR+) et au récepteur-2 du facteur de croissance épidermique humain (HER2+) ont été extraites et les intervalles de confiance à 95% ont été calculés. Les estimations globales ont été calculées par méta-analyses à effet randomisé. 54 études d’Afrique du Nord (n=12284 femmes avec un cancer du sein) et 26 études d’Afrique Sub-Saharienne (n=4737) étaient éligibles. Dans les deux régions, les études présentaient une hétérogénéité importante des estimations des ER+ (I²> 90%), avec la majorité des proportions entre 0,40 et 0,80 en Afrique du Nord et entre 0,20 et 0,70 en Afrique Sub-Saharienne. De même, une grande hétérogénéité a été observée pour PR+ et HER2+ (I²> 80%, dans tous les cas). Des analyses de méta-régression ont montré que la pourcentage de tumeurs ER+ était inférieure de 10% (4%, 17%) dans les études utilisant des fragments tumoraux archivés par rapport aux échantillons recueillis prospectivement, et inférieure de 9% (2%, 17%) dans les études ayant un taux de tumeurs de grade 3 ≥ 40% versus <40%. Pour les échantillons recueillis prospectivement, les proportions globales des tumeurs ER+ et triple négatives étaient 0,59 (0,56, 0,62) et 0,21 (0,17, 0,25), respectivement. Les limites de cette méta-analyse incluent l’absence de procédures standardisées dans les différentes études; la faible qualité méthodologique de nombreuses études en termes de représentativité de leurs séries de cas et de qualité du recueil des données, de la fixation, et des tests des récepteurs; et la possibilité que les femmes atteintes d'un cancer du sein aient pu participer à plus d'une étude.

**Conclusions:** Les données publiées des études les plus appropriées, menées à partir d’échantillons recueillis prospectivement sont concordantes : la majorité des cancers du sein en Afrique sont ER+. En l'absence de type dominant dans le continent, la mise à disposition des tests de récepteur doit être une priorité, surtout pour les femmes jeunes atteintes de la maladie à un stade précoce chez qui des modalités de traitement de leur type de tumeur adaptées au statut spécifique du récepteur ont le plus grand potentiel de réduire les années de vie perdues.

**PORTUGUESE TRANSLATION OF THE TITLE AND ABSTRACT**

**Receptores hormonais do cancro da mama em populações Africanas: revisão sistemática e meta-análise**

**Resumo**

**Contexto**: O cancro da mama é o cancro mais frequente nas mulheres africanas. Neste tipo de cancro a análise dos receptores hormonais é um factor essencial na avaliação do prognóstico (tumores com receptores de estrogénios negativos têm pior prognóstico) e na decisão terapêutica. Naquela região do globo não está definida com precisão a frequência de tumores com receptores de estrogénios negativos. Realizámos uma análise sistemática de todas as publicações relativas à frequência de tipos hormonais de cancro da mama em populações Africanas.

**Métodos e Resultados**: Foram feitas pesquisas na Medline, Embase e Global Health de todos os artigos publicados entre 1 de Janeiro 1980 e 15 Abril 2014. Foi extraída de cada publicação informação sobre a proporção de tumores positivos para os receptores de estrogénio (ER+), receptores de progesterona (PR+) e receptor-2 do factor de crescimento epidérmico humano (HER2+) e calculados os intervalos de confiança de 95%. As estimativas combinadas foram calculadas por meta-análise de efeitos aleatórios. Um total de 54 estudos realizados na África do Norte (n=12284 mulheres com cancro da mama) e 26 realizados na África Subsaariana (n=4737) foram identificados como elegíveis. Nas duas regiões, os estudos demonstraram uma grande heterogeneidade na proporção de tumores ER+ (I²> 90%), com a grande maioria das estimativas entre 0,40 e 0,80 no Norte de África e entre 0,20 e 0,70 na África Subsaariana. Observou-se igualmente uma grande heterogeneidade na proporção de tumores PR+ e HER2+ (I²> 80%, em todos os casos). A meta-regressão mostrou que a proporção de tumores ER+ era 10% (4%, 17%) mais baixa nos estudos baseados em amostras tumorais históricas (armazenadas em arquivo) do que em estudos baseados em amostras colhidas prospectivamente, e 9% (2%, 17%) mais baixa em estudos em que a percentagem de tumores de grau 3 era ≥40% versus <40%. Entre os estudos baseados em amostras colhidas prospectivamente, as proporções combinadas de tumores ER+ e tumores triplo-negativos eram 0,59 (0,56, 0,62) e 0,21 (0,17, 0,25), respectivamente. As limitações desta meta-análise incluem: a falta de estandardização nos procedimentos usados pelos vários estudos; a fraca qualidade metodológica de muitos estudos em termos da representatividade das suas séries de casos de cancro da mama; a qualidade deficiente dos procedimentos usados na colheita, fixação e teste hormonal das amostras tumorais; e a possibilidade de algumas mulheres com cancro da mama terem contribuído para mais de um estudo.

**Conclusões**: Os dados publicados pelos estudos mais fidedignos, os baseados em amostras colhidas prospectivamente, apontam para que a maioria dos cancros da mama em África seja ER+. Como não há predominância de nenhum tipo hormonal no continente africano a disponibilização de testes para receptores hormonais deve ser considerada uma prioridade, particularmente entre mulheres jovens que se apresentam num estadio precoce da doença, já que é neste grupo de doentes que o tratamento específico para o tipo hormonal tem o maior potencial para reduzir o número de anos de vida perdidos.
