# Supplementary material for: Receptor-Defined Subtypes of Breast Cancer in Indigenous Populations in Africa: A Systematic Review and Meta-Analysis
Source: PLoS Med. 2014 Sep 9;11(9):e1001720. doi: 10.1371/journal.pmed.1001720 (PMC4159229; doi:10.1371/journal.pmed.1001720)
Supplement: Text S2 — Protocol of the systematic review. (DOCX) [file pmed.1001720.s015.docx]

**Text S2: Protocol of the Systematic Review**

(Final version, May 2013)

**Title:**

Receptor-defined subtypes of breast cancer in native populations in Africa: a systematic review

**Review team:**

Amanda Eng, Valerie McCormack and Isabel dos Santos Silva

**Background:**

Breast cancer is the most common female malignancy in Africa [1]. Breast cancer incidence rates are lower in Africa than in the rest of the world, but mortality rates in certain African countries (e.g. Nigeria, Egypt, Ethiopia) are among the highest worldwide [1] due to the relatively poor survival from the disease in the continent.

Breast cancer subtypes can be identified on the basis of the tumour estrogen (ER), progesterone (PR) and human epidermal growth factor-2 (HER2) receptor status. These receptor-defined subtypes are not only an inherent feature of the epidemiology of this malignancy [[2](#_ENREF_2)] but they are also clinically relevant as they are major determinants of treatment options, disease outcomes and survival [[3](#_ENREF_3)]. ER+ tumours have a better prognosis and are more receptive to hormonal treatment [[4](#_ENREF_4)]. In Caucasian women, ER-positive (ER+) tumours predominate, with over 70% of breast tumours in US-born White women being ER+ [[5](#_ENREF_5)]. The proportion of ER+ tumours is lower among US-born Black women (about 60%) [[5](#_ENREF_5),[6](#_ENREF_6)], but the extent to which this is also reflected in Africa is not known. Some studies [[7](#_ENREF_7),[8](#_ENREF_8)] have reported a markedly higher proportion of ER-negative (ER-) or basal-like breast cancers in native populations in Africa, which may contribute to the poor survival from this malignancy, but others suggest that the relative frequency of the different subtypes in the continent may not differ substantially to that seen elsewhere [[9](#_ENREF_9)].

**Objectives:**

The main objective of this systematic review is to establish the frequency of breast cancer receptor-defined subtypes in native populations in Africa.

Specific aims are:

1. To estimate the prevalence of ER+, PR+ and HER2+ breast cancer tumours in the continent.
2. To estimate the prevalence of combined subtypes: luminal A (ER+ and/or PR+; HER2-), luminal B (ER+ and/or PR+; HER2+), HER2+ enriched (ER-; PR-; HER2+) and triple negative (ER-; PR-; HER2-) cases.
3. To identify sources of between-study heterogeneity in the prevalence of different types of receptor-defined subtypes.

**Search strategy:**

We plan to identify and review all published peer-reviewed studies that meet the eligibility criteria described below.

*Eligibility criteria*

Studies will be eligible if (all conditions need to be met):

- Setting: native African populations
- Type of studies: original report (primary data collection) including a population-based, consecutive or convenience series of newly diagnosed breast cancer cases. The series may or may not have been assembled as part of a case series, case-control, cohort or intervention study.
- Date: article published between 1^st^ January 1980 and 31^st^ August 2013
- Language: no restrictions were imposed (to ensure that any article published in English, French, Spanish, Portuguese or Afrikaans will be identified)
- Size: studies comprising a total of at least 50 breast cancer cases

*Exclusion criteria:*

Studies will be excluded if they focus exclusively on:

- non-humans
- male breast cancer ^a^
- African-American women
- non-Black African women (e.g. White or Coloured women in South Africa)
- metastatic breast cancer
- pregnant women
- specific treatment groups (where tumour receptor status testing was done prior to start of treatment)
- specific receptor-defined subtype(s) (e.g. triple negative cases)

Studies will also be excluded if:

- the total number of breast cancer cases included was <50
- reviews, conference abstracts and proceedings, and general discussion papers

^a^ Studies that included both male and female breast cancer cases recruited over a given period of time will not be excluded even if data are not presented separately by gender as the total number of male cases is likely to be rather small.

*Search databases:*

The following electronic databases will be searched:

- Medline
- Embase
- Global Health
- African Journals Online (AJO)
- Breast Health Global Initiative – INCTR Breast Cancer Control Library

The results and dates of each search will be recorded.

*Search terms:*

The initial search will be conducted using specific keywords to identify relevant papers. However, we anticipate that receptor status will not be the focus of many potentially eligible papers (and, hence, not a keyword) as these findings may simply be reported under patients’ characteristics. Thus, to minimise ascertainment bias the search will be broadened to ‘breast cancer’ in ‘Africa’.

*Hand searches:*

Reference lists of all included studies will be cross-checked to identify other potentially relevant studies. In addition, reference lists of reviews, conference papers and discussions articles – which will be ineligible for the review - will be searched and cross-checked.

**Title and abstract screening**

Literature searches of the electronic databases listed above will be conducted and the resulting citations will be downloaded to EndNote software, where duplicate citations will be removed. Any additional citations identified through hand-searches will be added to this database.

The titles and abstracts from this initial database will be screened by one reviewer and classified using the eligibility and exclusion criteria described above as:

- Yes, full paper to be retrieved and screened
- No, exclude
- Unclear

A sample of the abstracts will also be independently screened by a second reviewer. The reason for exclusion of papers from the review will be documented.

**Full-text screening**

The full-text article for all references classified as “Yes” or “Unclear” from the abstract screen will be retrieved and screened by one author to confirm reporting of receptor status. Any exclusion of articles from the review will be documented.

**Data abstraction**

The data extraction from each eligible paper will be carried out independently by two reviewers. A computerised data extraction form will be developed in Microsoft Access. This form will be pre-tested prior to its implementation (all three reviewers will independently extract data from 5 randomly selected studies) and their data compared. Any ambiguities in the data extraction form will be discussed and the form amended accordingly.

Data will be extracted on the following variables:

- Study identifiers: ID, author(s), year of publication
- Characteristics of the study and source of cases: country, study design (e.g. population-based, case series based on consecutive cases diagnosed over a defined time period, or collection based on convenience (opportunistic) samples), eligibility criteria, source of cases (e.g. hospital/clinic or cancer registry), study period and sample size;
- Demographic and reproductive-related variables (e.g. ethnicity, age and menopausal status at diagnosis, age at menarche, age at first birth, parity);
- Tumor characteristics (e.g. tumor morphology and grade; stage at presentation);
- Collection and storage conditions of the tumour specimens (e.g. fresh-frozen, formalin-fixed paraffin-embedded (FFPE) blocks);
- Receptor testing (e.g. timing of testing: retrospective based on archival samples or at the time of diagnosis); type of assay and positivity criteria used);
- Number of cases with available receptor status information, and number of positive and negative cases, as classified in the original article regardless of the criteria used to define positivity, for ER (ER+/ER-), PR (PR+/PR-) and HER2 (HER2+/HER2-). Where available, data will also be collected for combined subtypes: luminal A (ER+ and/or PR+; HER2-), luminal B (ER+ and/or PR+; HER2+), HER2+-enriched (ER-; PR-; HER2+) and triple negative (ER-; PR-; HER2-).

If there are multiple eligible papers from the same study only the one based on the largest sample size will be selected for inclusion in the systematic review.

Relevant data from each eligible study will be extracted independently by two reviewers. Each of them will complete the pre-tested standardised data extraction form in Microsoft Assess. The two resulting databases will be compared to identify discrepancies - these will be discussed and resolved by consensus.

**Assessment of Study Quality**

The two reviewers will independently use the data extracted from each study to assess their quality using a specifically-developed standardised quality assessment form. This assessment form will be developed to capture three domains:

- Potential for selection bias (e.g. study design)
- Potential for information bias, i.e. misclassification of receptor status (e.g. timing of tissue sample collection, specimen storage conditions, timing of receptor status testing, type of assay, positivity criteria)
- Availability of data on key variables (e.g. age at time of diagnosis, tumor grade and stage)

Quality scores will be calculated separately for each one of the three receptors as the type of assay and positivity criteria used will be receptor-specific.

A list of items for each one of the three domains will be developed. For each item, papers will be allocated a score ranging from 0 (if it does not meet the criteria or if the information provided is unclear) to a maximum to be defined (e.g. 1, 2 or 4, depending on the specific item). More weight will be given to items in the selection bias and misclassification domains. The overall quality of the study will be expressed as the sum of its item-specific scores. The higher the score the higher the methodological quality of the study, that is the lower the risk that its findings may have been affected by bias.

**Data analysis**

The extracted data will be analysed in STATA (Statistical Software version 12 (StataCorp, Texas).

Basic descriptive analyses will be conducted to summarise information about the study population (e.g. by region, country, type of study), timing of receptor testing, type of samples, etc.

Analyses will be stratified by region – North Africa and Sub-Saharan Africa - to reflect differences in the ethnic composition of their populations

For each receptor, the reported proportion of receptor-positive breast cancers (*prop*) will be calculated as (number of receptor-positive tumours)/(*n*=number of tumours with known receptor status). Standard errors (SE) and 95% confidence intervals (CI) for *prop* will be estimated as √*prop(1-prop)/n* and *prop±1.96xSE(prop)*, respectively

Pooled proportion estimates will be calculated using random effects models. To examine potential sources of heterogeneity, study-specific estimates will be stratified according to relevant clinical factors (e.g. age, year and menopausal status at diagnosis; tumour stage and grade) and methodologically relevant variables (e.g. study design; timing of receptor testing; specimen storage conditions; study quality). Between-study heterogeneity will be formally assessed using I^2^ and the *P*-value for heterogeneity (Cochrane’s Q statistic) [10]. Summary estimates from sub-groups will be formally compared using meta-regression. The findings will be tabulated and/or displayed graphically using forest plots.

Depending on the characteristics of the studies included in the review, and on the data reported, we will also attempt to compare the frequency of different receptor-defined subtypes of breast cancer between Black African and Western White and Black women (e.g. based on data from multi-country studies) and between African and non-African women in Africa (e.g. for studies reporting on different ethnic groups within South Africa).

Small study bias will be assessed via funnel plots and the Egger funnel plot asymmetry test [11].

**References**

1. International Agency for Research on Cancer. GLOBOCAN 2008. <http://globocaniarcfr/>.

2. Yang XR, Chang-Claude J, Goode EL, Couch FJ, Nevanlinna H, et al. (2011) Associations of breast cancer risk factors with tumor subtypes: a pooled analysis from the Breast Cancer Association Consortium studies. J Natl Cancer Inst 103: 250-263.

3. Blows FM, Driver KE, Schmidt MK, Broeks A, van Leeuwen FE, et al. (2010) Subtyping of breast cancer by immunohistochemistry to investigate a relationship between subtype and short and long term survival: a collaborative analysis of data for 10,159 cases from 12 studies. PLoS Med 7: e1000279.

4. Davies C, Godwin J, Gray R, Clarke M, Cutter D, et al. (2011) Relevance of breast cancer hormone receptors and other factors to the efficacy of adjuvant tamoxifen: patient-level meta-analysis of randomised trials. Lancet 378: 771-784.

5. Jemal A, Fedewa SA (2012) Is the prevalence of ER-negative breast cancer in the US higher among Africa-born than US-born black women? Breast Cancer Res Treat 135: 867-873.

6. Surveillance Epidemiology and End Results (SEER) Program ([www.seer.cancer.gov](http://www.seer.cancer.gov)) SEER*Stat Database: Incidence, SEER 17 Regs Research Data + Hurricane Katrina Impacted Louisiana Cases, Nov 2010 Sub (1973–2008 varying), Linked To County Attributes. 2010. Total U.S., 1969–2009 Counties, National Cancer Institute, DCCPS, Surveillance Research Program, Cancer Statistics Branch released April 2011 (updated 10/28/2011). Ref Type: Report

7. Bird PA, Hill AG, Houssami N (2008) Poor hormone receptor expression in East African breast cancer: evidence of a biologically different disease? Ann Surg Oncol 15: 1983-1988.

8. Huo D, Ikpatt F, Khramtsov A, Dangou JM, Nanda R, et al. (2009) Population differences in breast cancer: Survey in indigenous african women reveals over-representation of triple-negative breast cancer. J Clin Oncology 27 (27): 4515-4521.

9. Adebamowo CA, Famooto A, Ogundiran TO, Aniagwu T, Nkwodimmah C, et al. (2008) Immunohistochemical and molecular subtypes of breast cancer in Nigeria. Breast Cancer Res Treat 110: 183-188.

10. Higgins JP, Thompson SG, Deeks JJ, Altman DG (2003) Measuring inconsistency in meta-analyses. Br Med J 327: 557-560.

11. Egger M, Davey Smith G, Schneider M, Minder C (1997) Bias in meta-analysis detected by a simple, graphical test. Br Med J 315: 629-634.
