# Supplementary material for: Receptor-Defined Subtypes of Breast Cancer in Indigenous Populations in Africa: A Systematic Review and Meta-Analysis
Source: PLoS Med. 2014 Sep 9;11(9):e1001720. doi: 10.1371/journal.pmed.1001720 (PMC4159229; doi:10.1371/journal.pmed.1001720)
Supplement: Text S3 — Terms used in the literature search. (DOCX) [file pmed.1001720.s016.docx]

**Text S3. Terms used in the literature search**

*Search terms used:*

Exp breast neoplasms/ (MeSH)

**OR**

(breast or mammary) adj2 (cancer* or neopl* or tumour or tumor or carcin* or malig*)

**AND**

Exp Africa/ (MeSH)

**OR**

Africa OR Morocco OR Algeria OR Libya OR Egypt* OR Chad OR Sudan OR Ethiopia OR Somalia OR Angola OR Niger* OR Maurit* OR Congo OR Kenya* OR Tanzania OR Mozambique OR Zambia OR Zimbabwe OR Botswana OR South Africa OR Namibia OR Mali OR Tunisia* OR Gabon OR Sahara* OR Ghana OR Djibouti OR Rwanda* OR Malawi OR Swaziland OR Lesotho OR Cameroon OR Senegal OR Liberia OR Sub-Sahara*

**Limits**: Humans, 1980-Current

**African Journals Online**

Keyword: breast (cancer OR neoplasm* OR carcinoma* OR tumour* OR tumor*)
